# Differential annotation of converted metabolites (DAC-Met): Exploration of Maoto (Ma-huang-tang)-derived metabolites in plasma using high-resolution mass spectrometry

**DOI:** 10.1007/s11306-020-01681-3

**Published:** 2020-04-25

**Authors:** Katsuya Ohbuchi, Nozomu Sakurai, Hiroyuki Kitagawa, Masaru Sato, Hideyuki Suzuki, Hirotaka Kushida, Akinori Nishi, Masahiro Yamamoto, Kazuhiro Hanazaki, Masanori Arita

**Affiliations:** 1 Tsumura Kampo Research Laboratories, Tsumura & CO, Ibaraki, 300-1192 Japan; 2 National Institute of Genetics, Mishima, Shizuoka 411-8540 Japan; 3 Kazusa DNA Research Institute, Kisarazu, Chiba 292-0818 Japan; 4 Department of Surgery, Kochi Medical School, Kochi University, Kochi, 783-8505 Japan; 5 RIKEN Center for Sustainable Resource Science, Yokohama, 230-0045 Japan

**Keywords:** Metabolomics, Traditional herbal medicine, Kampo medicine, Natural products, Metabolite annotation

## Abstract

**Introduction:**

Traditional herbal medicine (THM) contains a vast number of natural compounds with varying degrees of pharmacological activity. To elucidate the mode of action, comprehensive metabolite profiling in the plasma before and after administration of THM is essential.

**Objective:**

The aim of this study was to explore and identify/annotate converted metabolites after administration of THM in humans.

**Methods:**

We performed untargeted metabolome analysis of human plasma collected before and after administration of maoto (ma-huang-tang), a traditional Japanese Kampo medicine. Maoto-derived metabolites were then selected and annotated following the DAC-Met strategy, which is an annotation method that uses mass differences of major metabolic reactions among the detected peaks and a differential network analysis.

**Results:**

About 80% of maoto-derived components were found to be converted forms. Following DAC-Met, the structures of 15 previously unidentified metabolites were determined, and five of these were later confirmed with authentic standards. Using published literature, we also reconstructed the metabolic pathway of maoto components in humans. A kinetic time-course analysis revealed their diverse kinetic profiles.

**Conclusion:**

The results demonstrated that time-resolved comprehensive metabolite profiling in plasma using the DAC-Met strategy is highly useful for elucidating the complex nature of THM.

**Electronic supplementary material:**

The online version of this article (10.1007/s11306-020-01681-3) contains supplementary material, which is available to authorized users.

## Introduction

Natural compounds have received considerable attention as potential drug leads in pharmaceutical development. They represent a diverse and biologically active group of chemicals (Harvey et al. 2015), some of which could be highly promising as new drug candidates. Investigation of traditional herbal medicines (THMs) is of interest because they are widely used worldwide; in Japan, they are referred to as Kampo medicine and manufactured in compliance with Japanese good manufacturing practice (GMP) defined by Japanese law to ensure quality control (Kono et al. 2015). More than 80% of medical doctors in Japan prescribe Kampo medicine for various symptoms from flu to climacteric disorders and indefinite complaints, which cannot be sufficiently treated using conventional Western medical therapies alone (Grayson 2011). Similarly, THMs are popular in many Asian countries such as China, Korea, and Indonesia. Elucidating their mode of action contributes not only to the wider application of THMs but also to novel therapies for the treatment of unmet medical needs.

THMs contain a large number of natural compounds with diverse activities; they act on various sites in the body and the cumulative results of complex interactions are manifested as pharmacological effects on the body. Important metabolic process includes various conjugation reactions affecting the delivery of the active components to target sites (Kaneko et al. 2017), and hydrolysis by the microbiome is essential for exerting their pharmacological effects (Ikarashi and Mizoguchi 2016; Oh and Kim 2016; Yamamoto et al. 2000). To understand the complex nature of THM metabolism, time-resolved comprehensive compound profiling is essential, where the profiling covers not only the native components of THMs but also their metabolites converted by microbiome and metabolic enzymes in the liver and gastrointestinal tissue. The profiling, therefore, needs to include the THM itself as well as plasma samples pre- and post- administration with multiple timepoints.

We present a new strategy, DAC-Met (differential annotation of converted metabolites), to explore THM-derived metabolites in plasma using high-resolution mass spectrometry. As a case study, we chose ‘maoto’ (ma-huang-tang), a Kampo medicine decoction consisting of only four ingredients (ephedra leaf, apricot kernel, cinnamon bark, and licorice root), widely prescribed for febrile symptoms in Asian countries (Nabeshima et al. 2012). Human plasma samples were collected before and after administration of maoto at seven timepoints (Kitagawa et al. 2019a), and were analysed using an LC-Orbitrap mass spectrometer. In this untargeted analysis, we characterized maoto-derived conjugation products based on their mass difference, stable isotopic peaks, and mass chromatograms acquired with tandem mass spectrometry (MS/MS) from among the detected peaks, and we identified previously unknown derivatives. The strategy and methodology employed herein can contribute to a better understanding of the pharmacokinetics of natural compounds in plasma and extend the applicability of untargeted metabolomics to THM.

## Materials and methods

### Clinical study

The trial was conducted at the Kochi Medical School in September 2016. The study protocol was described previously (Kitagawa et al. 2019a). Four male subjects were enrolled in this study. Maoto was orally administered at a dose of 7.5 g. Blood samples were collected before and 0.25, 0.5, 1, 2, 4, and 8 h after administration of maoto. Plasma samples were then prepared and stored at or below − 70 °C prior to analysis.

### Maoto extract

Maoto is an extracted mixture of Ephedrae Herba (ephedra), Armeniacae Semen (apricot), Cinnamomi Cortex (cinnamon), and Glycyrrhizae Radix (licorice) combined at a ratio of 10:10:8:3 (32.3%:32.3%:25.8%:9.6%). Briefly, maoto is made by mixing the herbs, followed by extraction using hot water, and finally the extract is made into a powder. Guidelines for quality management, such as the species of these constitutive herbs, are defined in the Japanese Pharmacopeia. The dry powdered extracts of maoto (Lot No. 341115500), its constituent herbs, ephedra (Lot No. 2160027070; *Ephedra sinica* Stapf, *Ephedra intermedia* Schrenk et C.A. Meyer or *Ephedra equisetina* Bunge), cinnamon (Lot No.2160027080; *Cinnamomum cassia* Blume), apricot (Lot No.2160027060; *Prunus armeniaca* Linne, or *Prunus sibirica* Linne), licorice (Lot No.2160027090; *Glycyrrhiza uralensis* Fisher or *Glycyrrhiza glabra* Linne) produced by spray-drying were supplied by Tsumura & Co. (Tokyo, Japan).

### In vivo study

Male Sprague–Dawley (SD) rats (age 7 weeks) were purchased from Japan SLC, Inc. (Shizuoka, Japan), and used for experiments at age 8 weeks following habituation. Rats were housed individually in a cage with paper chips and permitted free access to food and water. Room temperature was maintained at 23 °C with 60% relative humidity and a 12 h light–dark cycle (7:00–19:00). Rats were maintained and used for experiments according to the Guidelines for the Care and Use of Laboratory Animals of Tsumura & Co. All experimental procedures were carried with the approval of the Laboratory Animal Committee of Tsumura & Co.

Each of the constituent herbs of maoto were dissolved in distilled water (0.2 g/mL) and orally administered at a dose of 10 mL/kg to rats fasted for 16 h (n = 1). Rats were anesthetized with isoflurane before blood sampling, and whole blood was withdrawn through the abdominal inferior vena cava with dipotassium EDTA at 1 h after administration. Plasma was obtained by centrifugation at 1,000 × g for 15 min at 4 °C and stored at or below -70 °C until use.

### Untargeted metabolome analysis

Plasma sample (100 µL) or maoto extract powder (1 mg) were extracted with 300 µL of methanol and 1 mL of 75% MeOH (aq), respectively. For untargeted metabolome analysis, LC–MS was performed using an Agilent 1200 HPLC system (Agilent Technologies, Santa Clara, CA) coupled to an LTQ Orbitrap XL-MS system (Thermo Fisher Scientific, Inc., San Jose, CA), equipped with an electrospray source operating in either positive- or negative-ion modes. The spray voltage and capillary temperature were 4 kV and 300 °C, respectively. The analysis consisted of 2 scan events. Scan event 1 was a full mass type (Analyzer, FTMS; Resolution, 60,000). Scan event 2 was an MS/MS type (analyzer, Ion Trap MS; act type, collision-induced dissociation; normalized collision energy, 35.0%). An aliquot of the extracted sample (5 µL) was injected into a TSK gel ODS-100 V reversed-phase column (column size, 3.0 × 50 mm; particle size, 5.0 µm; Tosoh Corp., Tokyo, Japan). The column temperature was set at 40 °C. Mobile phases A (0.1% formic acid) and B (acetonitrile with 0.1% formic acid) were used with a gradient of 3% to 97% B from 0 to 15 min, 97% B from 15 to 20 min, 97% to 3% B from 20 to 20.1 min, and 3% B for 4.9 min before the next injection, at a flow rate of 400 µL/min. The data were acquired with Xcalibur software version 2.0.7 (Thermo Fisher Scientific, Inc.). The same procedures were performed using 75% (v/v) methanol as a negative control (mock).

### Data analysis

The mass chromatogram data in the Xcalibur’s raw file were converted to mzXML format using ProteoWizard software (Chambers et al. 2012) and processed with PowerGetBatch (Sakurai and Shibata 2017) software for peak detection, characterization**,** and alignment. The metabolite peaks that were detected in at least 2 samples and not detected in the mock samples were selected and used for further data processing. The detailed parameters for PowerGetBatch are referred to in Supplementary Material 6.

**Fig. 1:**
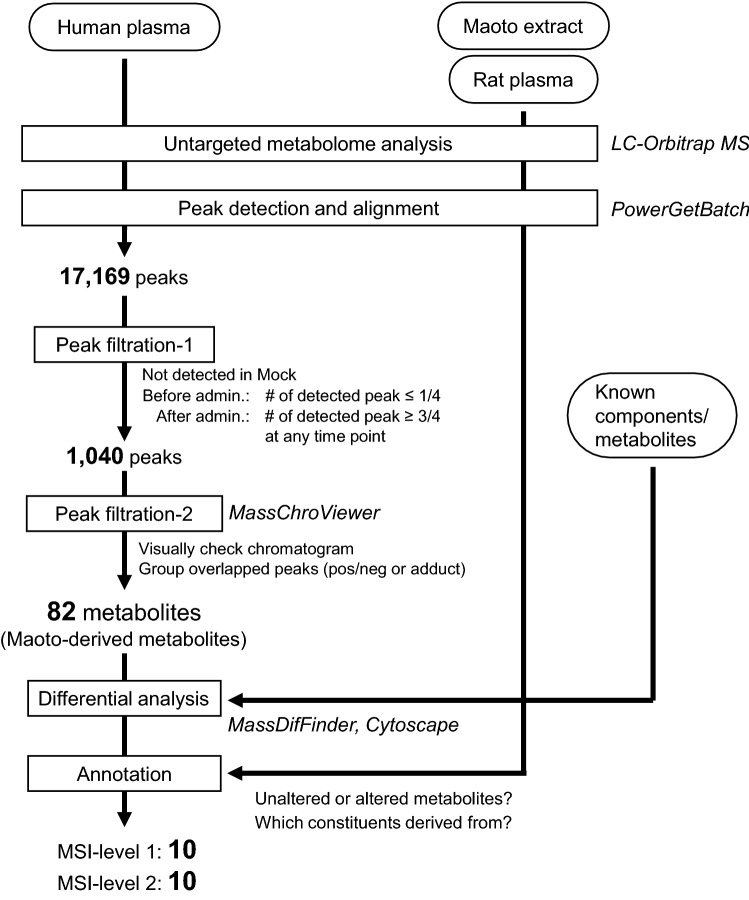
Overall analytical scheme for extraction and annotation of maoto-derived compounds. Plasma from the test subjects was analysed using LC-Orbitrap MS. Peak detection was performed with PowerGetBatch software. The maoto-derived compounds were explored by two-step peak filtration procedures. Differential analysis for exploration of conjugates was then performed using MassDifFinder and the results were visualized with Cytoscape software. Maoto-derived peaks were annotated by integrating the results for other samples; i.e., maoto extract and plasma collected from rats that were administered the constitutive herbs. Consequently, 20 maoto-derived metabolites were annotated (MSI-level 1 and 2) following the DAC-Met strategy

Peak filtration was performed systematically according to the number of good peaks as shown in Fig. 1. Filtered peaks were then visually checked using MassChroViewer (Sakurai and Shibata 2017) to confirm that the selected peaks were dependent on the administration of maoto (Supplementary Table 1). The metabolite annotation for the detected peaks was performed by searching compound databases or predicting elemental compositions using MFSearcher tool (Sakurai et al. 2018) based on the estimated *m*/*z* value and adduct ion. UC2 search mode of MFSearcher was applied for database searching with a given mass tolerance of 5 ppm**,** and the following compound databases were used: KNApSAcK (Afendi et al. 2012), KEGG (Kanehisa et al. 2019), HMDB (Wishart et al. 2018), LIPID MAPS (Fahy et al. 2009), and flavonoid database at the web-site https://metabolomics.jp. A 4-ppm mass tolerance and the ExactMassDB-HR2 database were adopted for prediction of elemental compositions. For identification and annotation in this study, identification confidence levels (MSI levels (Schymanski et al. 2014)) were applied to identified or annotated peaks. Regarding identification using authentic chemical standards, MS chromatograms and their comparison to those in plasma samples are represented in Supplementary Material 1. All tested standard compounds are listed in Supplementary Material 2.

Conjugates from maoto-derived metabolites were explored using MassDifFinder (https://www.kazusa.or.jp/komics/software/MassDifFinder), which checks the mass difference. MassDifFinder was developed with Java SE Development Kit (JDK 8, Oracle). The parameters in the setting file are shown in Fig. 3. The result of mass difference analysis was visualized using Cytoscape software (Morris et al. 2012).

Changes in the peak-intensity of maoto-derived metabolites were analysed by heatmapping. Missing values were replaced with the half-minimum value of the non-missing value in the same peak. After imputation, the values were normalized by auto scaling: the mean value of each peak area was subtracted from each individual area of the respective peak and divided by the standard deviation of the mean for the peak. Hierarchical clustering analysis was performed based on the Euclidean distance coefficient and Wards method using MetaboAnalyst (https://metaboanalyst.ca/) (Chong et al. 2018).

## Results

### Exploration and annotation of maoto-derived compounds

The overall analytical scheme consisted of multiple steps as shown in Fig. 1. First, untargeted metabolome analysis was performed using LC-Orbitrap MS in positive and negative ion modes. In addition to human plasma samples before and after administration of maoto, rat plasma samples after administration of maoto constituents and maoto alone were also measured to assist with annotation of the differential analysis. Candidate peaks were then detected and aligned across all samples to make a peak table using PowerGetBatch software (Sakurai and Shibata 2017). The number of detected peaks was 12,902 for human plasma samples and 5318 for maoto alone, with an overlap of 1051 peaks (total 17,169 peaks). Among them, 1040 peaks in the plasma were selected as maoto-derived candidates as they appeared only after administration in more than half of all samples at some timepoint, and never appeared in the mock samples. After removing peaks with ion counts less than 1000, the remaining peaks were manually checked from their chromatogram using MassChroViewer software (Sakurai and Shibata 2017), and 87 peaks were selected as reliable metabolite peaks (Supplementary Material 3). Five peaks originated from the same metabolite as other peaks (difference of ion modes or adducts, or in-source fragments [shown below]), and the total number of maoto-derived metabolites that underwent the annotation procedure was 82.

### Comparison with the known maoto-derived metabolites

**Fig. 2:**
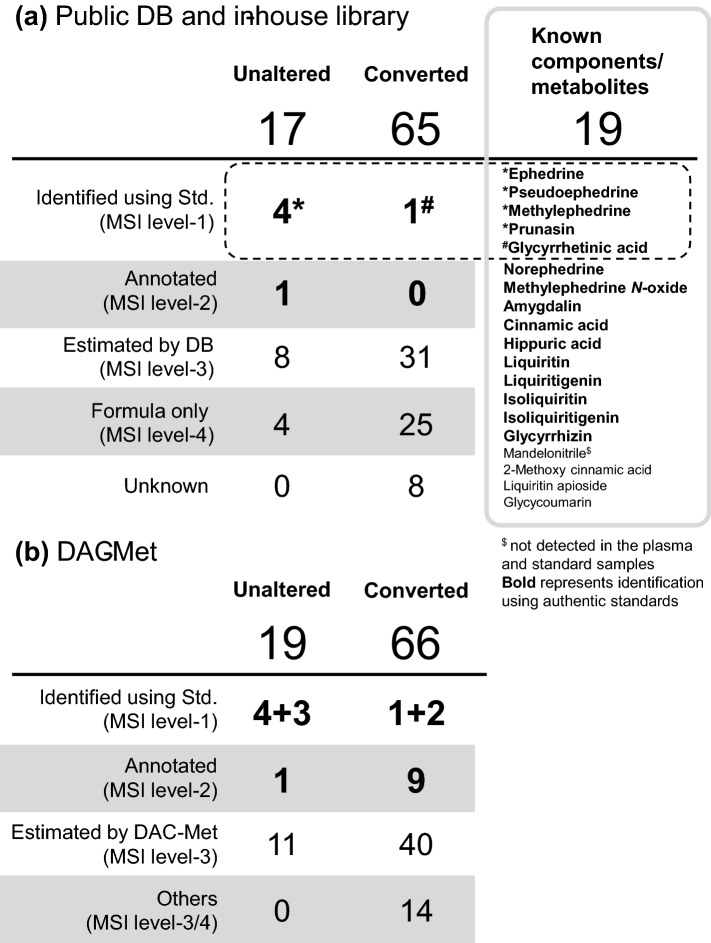
Breakdown of maoto-derived compounds and known components and metabolites of maoto. About 80% of the resultant metabolites were detected in plasma only and not in the maoto extract, which indicates that they were converted compounds metabolized in vivo. **a** The results of annotation using a public database search and in-house library. Five maoto-derived metabolites were identified as known maoto components (*, ^#^). Although about half of the unknown candidates were estimated by searching public databases, they were not the known components listed in the figure. Fifteen known maoto metabolites (represented in bold) were detected in plasma or maoto extract. **b** Summary of annotation according to the DAC-Met strategy. An additional 5 metabolites were identified using chemical standards and nine were annotated as conjugates based on differential analysis. About two-thirds of converted metabolites were estimated as conjugates of the detected peaks

Previously, 19 metabolites have been reported in maoto extract and/or in the plasma after maoto-administration using optimized targeted analyses (Kitagawa et al. 2019a; Nishi et al. 2017) (Fig. 2a, Supplementary Material 3). Five compounds (ephedrine, pseudoephedrine, methylephedrine, prunasin, and glycyrrhetinic acid [GA]) were also observed in our selected peaks (dotted line in Fig. 2a) and were confirmed using the authentic chemical standards according to retention time (RT) and accurate mass (MSI level-1, Supplementary Table 2). Most of the remaining 14 compounds were detected in maoto extract or rat plasma, but not in human plasma due to their low concentration (i.e., rats were administered a higher dose than humans; see Methods). Among these, 10 were identified using authentic standards (represented in bold in Fig. 2a). Hippuric acid, the metabolic product of cinnamic acid and ephedrine (Baba et al. 1986; Chen et al. 2009), was detected in human plasma pre- and post-administration, and it was not chosen as a maoto-derived metabolite. Mandelonitrile was not detectable in our analysis platform due to its insufficient ionization rather than its concentration (confirmed using the authentic standard).

### Standard annotation of 82 metabolites

Among the selected 82 metabolites, 17 were detected both in the plasma and maoto extract (Fig. 2a and Supplementary Material 3), indicating that they were absorbed into the blood without any metabolic modification. These included ephedrine, pseudoephedrine, methylephedrine, and prunasin (MSI level-1). Nine metabolite structures were estimated by database search using accurate mass and published literature as a reference (MSI level-2 and 3). Among them, N4651/N4653 was annotated as *p*-hydroxybenzylmalonic acid (*p*-HBMA), since it was abundant in licorice root (Li et al. 2016) and the peak was also detected in maoto extract and plasma from licorice-administered rats (Supplementary Table 1). The remaining 4 metabolites did not have a match in the database, although their chemical formulas could be assigned (MSI level-4).

One of the 65 candidates detected only in the plasma was glycyrrhetinic acid (MSI level-1). As many as 31 metabolite structures were estimated by the database and literature search (MSI level-3). Elemental composition was estimated for 25 compounds (MSI level-4), and 8 remained unknown. Thus, metabolized structures were difficult to identify in a standard annotation process using authentic chemical standards and database search.

### Network analysis of conjugated compounds

**Fig. 3:**
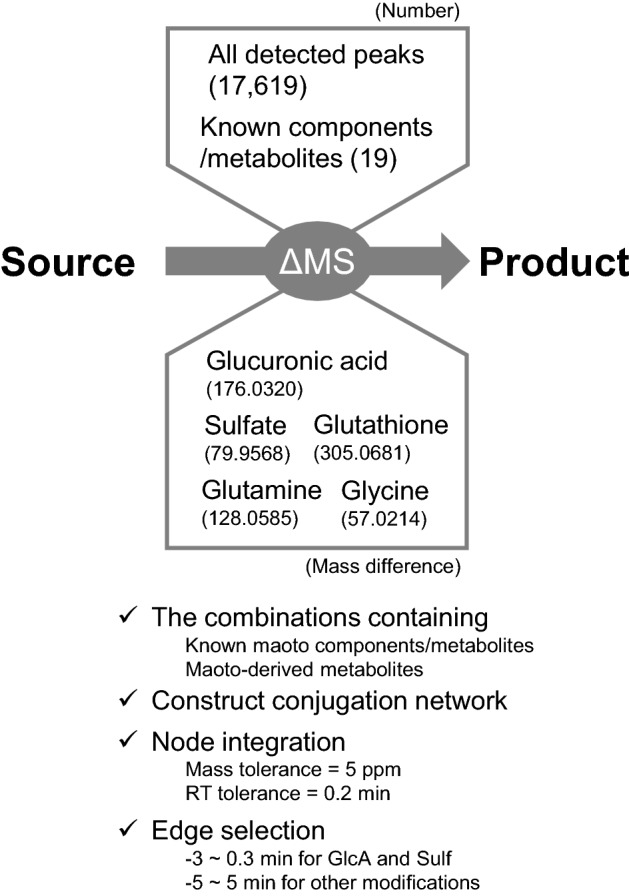
Differential analysis using MassDifFinder and Cytoscape software. MassDifFinder makes it possible to extract combinations of query peaks whose mass difference matches with the specified mass. Five conjugation reactions were specified, and the combination of sources and products including the maoto-derived metabolites or the known maoto components were extracted and visualized using Cytoscape software. The constructed network was refined according to mass and retention time differences

**Fig. 4:**
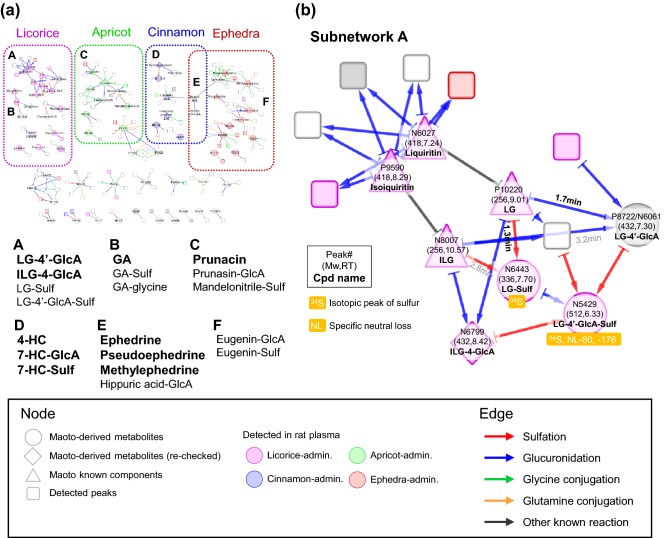
Conjugation network from differential analysis. **a** The conjugation network was constructed from differential analysis and known modifications. Nodes and edges represent metabolites and modifications, respectively. The herbal origin of nodes is colored corresponding to licorice, apricot, cinnamon, and ephedra according to the rat plasma analysis. Subnetworks were grouped based on herbal origin (dotted line). Four and nine conjugates in the subnetworks, labelled A-F, were identified (represented by bold, MSI level-1) and estimated (MSI level-2). **b** Support information for the annotation in subnetwork A. Peak number, deionized molecular weight (Mw), RT**,** and compound (Cpd) name are presented on the node. The detection of ^34^S peak and specific neutral loss are also included. The RT differences mentioned in Results section are described

To improve annotation for the maoto-derived metabolites, we focused on the mass difference arising from 5 major conjugation reactions with glucuronic acid, sulfate, glutamine, glycine, and glutathione using MassDifFinder software (Fig. 3) and represented the relationship as a conjugation network (Fig. 4a and higher-resolution figure in Supplementary Material 4). First, we extracted MS peak features that had the specific mass difference from maoto-derived metabolites and known metabolites (tolerance = 5 ppm) shown in Fig. 3. Peaks with close RT (tolerance = 0.2 min) were merged except for the peaks from ephedrine and psedoephedrine. Metabolite and peak pairs that showed specific RT differences were then highlighted: between − 3 and 0.2 min for glucuronidation and sulfation (in the reverse-phase LC column, the addition of glucuronate or sulfate generally shortens the RT), and -5 and 5 min for other conjugations. Known modifications such as hydrolysis of glucose were added (“Other known reaction” in Fig. 4a). Finally, the herbal origin of nodes (i.e., metabolites and peaks) was superimposed on the network by considering the measurement results of plasma from rats that were treated with the individual constitutive herbs of maoto (colored in Fig. 4 corresponding to licorice, apricot, cinnamon, and ephedra). Through the network representation, we were able to estimate 53 metabolites in human plasma as conjugates or source compounds of other detected peaks.

We rechecked the chromatogram of the adjacent nodes of the maoto-derived metabolites in the conjugation network and identified 3 additional peaks as maoto-derived metabolites (shown by diamonds in Fig. 4, Supplementary Material 3).

### Re-annotation of transformed products and estimation of the metabolic pathways

For each subnetwork from A to F in Fig. 4a, we re-annotated metabolic conjugates. Herbal origin estimated by the rat plasma data and ^34^S peak and the specific neutral losses were important support for this re-annotation as follows (Fig. 4b, Supplementary Material 4). We showcased our methodology by annotating the maoto-derived metabolites in subnetwork A (Fig. 4b).

**Fig. 5:**
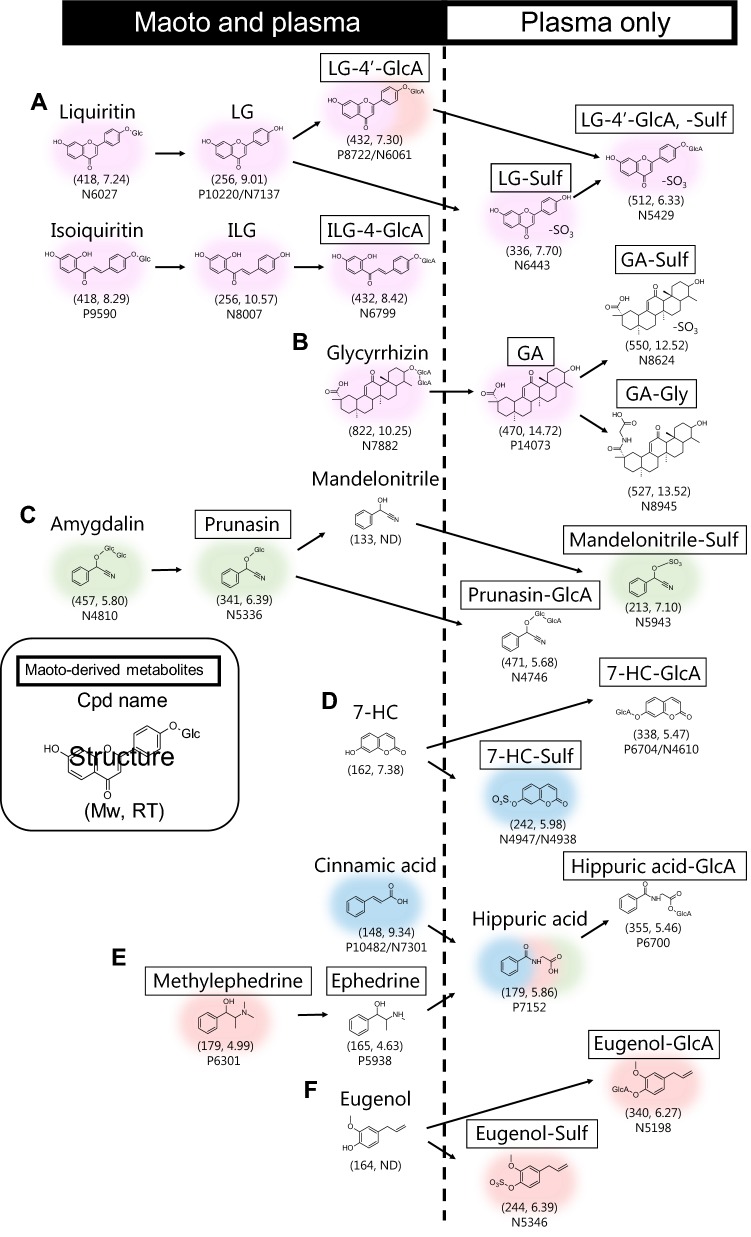
Expected metabolic pathway of maoto components. The expected metabolic pathway of maoto components was constructed based on the results of the differential analysis. Deionized mass (nominal) and retention time are represented in parentheses. Maoto-derived metabolites are surrounded by frames. Background colors represent the herbal origin. The compounds are divided into metabolites detected both in the maoto and plasma and those detected in the plasma only

Subnetwork A from licorice contained nodes whose deionized masses were 418, 256, 336, 432 and 512 Da (liquiritin, isoliquiritin, liquiritigenin (LG), isoliquiritigenin (ILG), P8722/N6061, N6443, N6799, and N5429, respectively in Fig. 4b). The compounds with mass of 418 and 256 Da in licorice were known licorice components (liquiritin, isoliquiritin, LG, and ILG). The compounds with mass 336, 432, and 512 Da were predicted as their sulfate, glucuronide, and both, respectively. Indeed, the spectra of sulfates were associated with ^34^S peaks and the specific neutral loss of sulfate (labelled with ^34^S and NL-80 in Fig. 4b). In general, conjugation of glucuronate or sulfate results in shorter RT. For example, sulfation and glucuronidation of 7-hydroxycoumarin (7-HC) shortened its RT by about 1.4 and 1.9 min, respectively (Fig. 5). The RT differences of mass 336 Da (N6443) and 432 Da (P8722/N6061) peaks from the LG peak were 1.3 and 1.7 min, respectively, whereas the differences from the ILG peak were 2.8 and 3.2 min, respectively (Fig. 4b). From this time difference, P8722/N6061 and N6443 were estimated as LG glucuronide and sulfate. N6799, another candidate with mass 432 Da, was estimated as ILG glucuronide. The accurate structure and MS/MS chromatogram of P8722/N6061 and N6799 were confirmed as LG-4′-glucuronide and ILG-4-glucuronide (MSI level-1, Supplementary Material 2, MS/MS chromatogram of P8722 and N6061 in Supplementary Material 1) with our chemical synthesis of 5 glucuronidation products of LG and ILG (LG-4′-glucuronide, LG-7-glucuronide, ILG-2-glucuronide, ILG-4-glucuronide, and ILG-4′-glucuronide) (Matsumoto et al. 2015). The geometric positions of sulfate remained unknown because sulfates were extremely difficult to chemically synthesize; the identification level of N6443 (LG sulfate) and N5429 (LG-4′-glucuronide sulfate) remained MSI level-2. We also annotated the candidates in the other subnetworks and found metabolites from GA, prunasin, HC, hippuric acid and eugenin (described in Supplementary Material 4). The result of annotation following DAC-Met is summarized in Fig. 2b. Combining literature knowledge and the rat results, we reconstructed the metabolic pathway of maoto components (Fig. 5). The pathway demonstrated that many maoto-derived metabolites in plasma existed as metabolized forms.

### Confirmation of re-annotation results through the kinetic time-course

**Fig. 6:**
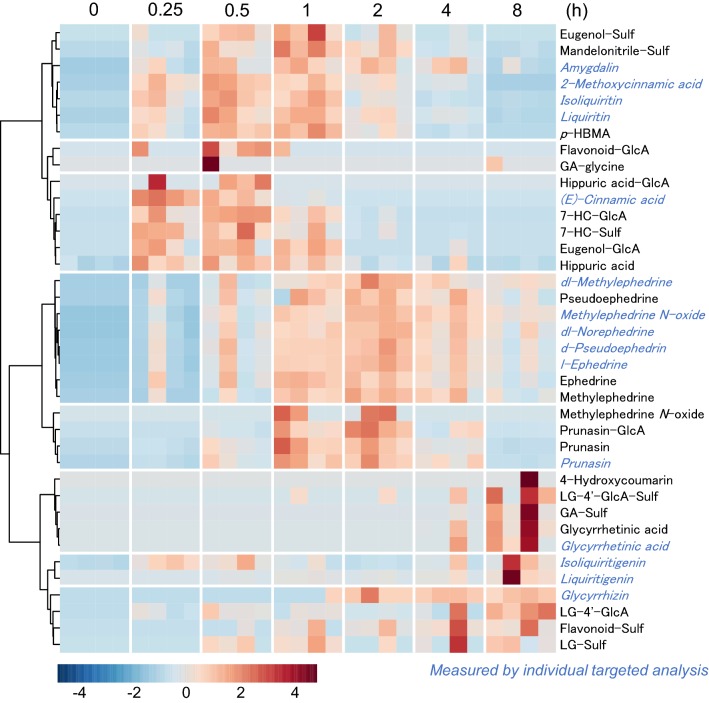
Kinetic time-course of maoto components and metabolites. The kinetic time course of maoto components and metabolites are represented by a heatmap. Regarding the blue-colored components, plasma concentration was used from a previous report (Kitagawa et al. 2019b). The intensities of maoto-derived metabolites and concentrations of maoto components were auto-scaled across all experimental samples

Clustering analysis of the kinetic time-course of metabolites can further improve the reliability of annotation. The time-course of four subjects was visualized as a heatmap using the normalized peak area (see Methods) and concentration of maoto components from a preceding research (Kitagawa et al. 2019b). The time-course of maoto components in the untargeted analysis matched well with those in the targeted quantitative analysis (Fig. 6 and the heat map consisting of mean values in Supplementary Material 5). The glucuronides, sulfates**,** and their source metabolites clustered well except for the microbiome metabolic products: de-glycosylated products of glycyrrhizin and (iso) liquiritin appeared several hours later than their original forms in the plasma. The heatmap also delineated large differences in treatment responses among individual subjects and maoto components. For example, the absorption of ephedrine analogues in 1 subject was faster than that in other subjects.

## Discussion

Herein, we presented the novel DAC-Met strategy as a means for exploring THM-derived metabolites in human plasma. They are mostly in metabolized forms that are difficult to identify using public databases only. In addition, metabolized forms are difficult to chemically synthesize. Therefore, an improved methodology of annotation for such metabolites is necessary. In the DAC-Met strategy, we focused on mass difference, RT shift, rat study data**,** and kinetic time-course to improve annotation. In the maoto study, we identified 10 compounds including 4 conjugates by comparison with standard compounds (MSI level-1), and estimated 10 compounds (MSI level-2) from untargeted analysis. Finally, we were able to construct the metabolic pathway of maoto components in humans.

Japanese Kampo medicine is prescribed according to the Kampo diagnosis, which emphasizes patient-based flexible formulations corresponding to the patient’s symptoms (Yu et al. 2006). The converted metabolites in plasma would reflect the host’s response predicted by the Kampo diagnosis. Since the concept of the diagnosis is not fully elucidated, it has been presumed to estimate the host’s ability to absorb and metabolize Kampo compounds, which leads to identifying unknown biomarkers reflecting the reactivity to a specific Kampo medicine. In this study, kinetic profiles of ephedrine derivatives, and glycyrrhetinic acid derivatives clearly differed among the 4 subjects. If this difference correlates with the efficacy of maoto, it suggests that such biomarkers would be useful for making the Kampo diagnosis. Therefore, it is important to explore the converted metabolites in plasma after administration of Kampo medicines and further identify the relationship between converted metabolites and phenotypes of the Kampo medicine using additional specimens.

Time-course analysis revealed another aspect of the pharmacology of maoto. Maoto-derived compounds exhibited diverse kinetic profiles in plasma from 15 min to 8 h after administration. We previously reported that maoto administration influences plasma amino acids and lipid mediators (Kitagawa et al. 2019a). Among them, docosahexaenoic acid, eicosapentaenoic acid, and arachidonic acid were increased 15 min after administration of maoto, and essential amino acids were decreased 1 h after administration. These time-dependent effects might be correlated with the kinetics of maoto-derived metabolites, which can enable the identification of responsible components. Many metabolites originating from licorice appeared 4–8 h after administration. These metabolites likely contribute to the long-lasting efficacy of maoto.

Converted metabolites are likely to be involved in the mode of action of maoto as active compounds. Liquiritin and isoliquiritin are converted to their aglycon, LG and ILG, via hydrolysis of glucose by the microbiome. LG and ILG are known to have various pharmacological effects including anti-inflammatory activity (Ramalingam et al. 2018). However, their plasma concentrations after administration of licorice-containing THM are too low to be effective (Kitagawa et al. 2019a). Conversely, the concentrations of IG and ILG glucuronide conjugates are much higher than those of the parent compounds (Matsumoto et al. 2015). It has been reported that flavonoid-glucuronide is activated as an aglycon by *β*-glucuronidase in tissue macrophages (Kaneko et al. 2017). Likewise, the metabolites in subnetwork A in Fig. 5 could be interconverted and contribute to the pharmacological effect of maoto. Eugenol has been reported to be have anti-inflammatory and free radical-scavenging activities (Fujisawa and Murakami 2016). Although eugenol is not detectable using LC–MS, its glucuronide and sulfate forms, the main conjugates of eugenol in humans (Fischer et al. 1990), were detected in this study. It is possible that eugenol conjugates would become active though the same process as observed for flavonoid-glucuronide.

Other annotated conjugates also contribute to understanding the effects of maoto administration. Glycyrrhetinic acid was expected to be converted to its sulfate. 18*β*-glycyrrhetyl-3-*O*-sulfate was detected in a patient who had taken a licorice-containing Kampo formula for 3 years**,** and its plasma concentration was higher than that of glycyrrhetinic acid (Ishiuchi et al. 2019). It was suggested that the 3-*O*-sulfate could be involved in the onset of hypokalemia, which is a major adverse effect of licorice-containing medicines.

In the DAC-Met strategy, there are several important factors for annotation. Firstly, RT and MS/MS information about known components are valuable. Since Kampo medicine is standardized according to Kampo GMP guidelines in Japan, it is worthwhile to identify and organize more components of Kampo medicine in a database. Additionally, the inclusion of data from different types of samples would be quite helpful. In this study, we used maoto extract and plasma from rats that were administered the constituent herbs. The results of the maoto extract analysis were important for expanding the differential analysis; it is possible in the network analysis to connect the conjugates to the maoto compounds, which could not be detected in human plasma in the unaltered form (e.g., 7-HC was detected in the maoto extract but not in human and rat plasma). Rat plasma was used for estimating the herbal origin. Since the plasma was collected 1-h post-administration, some slowly appearing metabolites such as glycyrrhetinic acid sulfate were not detectable. The profiling of plasma, therefore, needs to include multiple timepoints posttreatment. Additionally, overlaying other sample sets onto the network is also helpful for further annotation. For example, analysis of rodent plasma after administration of a single ingredient such as liquiritin or isoliquiritin would help to confirm the estimation subnetworks A.

The accuracy of peak picking should be further improved in the future to facilitate wider application of our strategy in other studies. We attempted to minimize subjective judgement from our strategy; however, manual checking of the chromatograms was necessary to filter the metabolite peaks that were clearly caused by maoto administration. Although PowerGetBatch software is advantageous for detecting low-intensity peaks (Sakurai and Shibata 2017), and we detected more than 12,000 peaks in plasma, the software was still incomplete for eliminating both false positive peaks in posttreatment samples and false negative peaks at trace levels in pretreatment samples. MassChroViewer tool was also used for rapid manual checking in this study, but further improvement of the accuracy of peak picking is required.

## Electronic supplementary material

Below is the link to the electronic supplementary material.

MS spectra of the authentic chemical standards and test samples. Supplementary file1 (PDF 455 kb)

Information about all tested chemical standard compounds. Supplementary file2 (XLSX 15 kb)

Information about maoto-derived metabolites and maoto known components/metabolites. Supplementary file3 (XLSX 46 kb)

Mass difference network and subnetwork B-F. Supplementary file4 (PDF 581 kb)

Kinetic time-course of maoto components and metabolites represented as mean values of 4 human individuals. Supplementary file5 (PDF 89 kb)

The parameters for PowerGetBatch. Supplementary file6 (ZIP 24 kb)
