# Supplementary material for: Differential annotation of converted metabolites (DAC-Met): Exploration of Maoto (Ma-huang-tang)-derived metabolites in plasma using high-resolution mass spectrometry
Source: Metabolomics. 2020 Apr 25;16(5):63. doi: 10.1007/s11306-020-01681-3 (PMC7183508; doi:10.1007/s11306-020-01681-3)
Supplement: Supplementary file 1 — MS spectra of the authentic chemical standards and test samples. Supplementary file1 (PDF 455 kb) [file 11306_2020_1681_MOESM1_ESM.pdf]

Article title:

Differential Annotation of Converted Metabolites (DAC-Met): Exploration of Maoto (Ma-huang-tang)-derived Metabolites in Plasma Using High-resolution Mass Spectrometry.

Journal name:
Metabolomics

Author names:
Katsuya Ohbuchi[1]*, Nozomu Sakurai[2,3], Hiroyuki Kitagawa[4], Masaru Sato[3], Hideyuki Suzuki[3], Hirotaka Kushida[1], Akinori Nishi[1], Masahiro Yamamoto[1], Kazuhiro Hanazaki[4], Masanori Arita[2,5]

Affiliation:
[1]*Tsumura Kampo Research Laboratories, Tsumura & CO., Ibaraki 300-1192, Japan*
[2]*National Institute of Genetics, Mishima, Shizuoka 411-8540, Japan*
[3]*Kazusa DNA Research Institute, Kisarazu, Chiba 292-0818, Japan*
[4]*Department of Surgery, Kochi Medical School, Kochi University, Kochi 783-8505, Japan*
[5]*RIKEN Center for Sustainable Resource Science, Yokohama 230-0045, Japan*

* Corresponding author.
E-mail: oobuchi_katsuya@mail.tsumura.co.jp (KO)

Supplementary Figure 1

The product ion tandem mass spectra of the authentic chemical standards and test samples (plasma or maoto extract). a) The 23 authentic chemical standards, b) comparison between MS spectra of the standard and test sample, and c) MS spectrum of N5924. N5924 had the specific neutral losses of glucuronide (176) and sulfate (80).

Ephedrine $[M+H]^+$

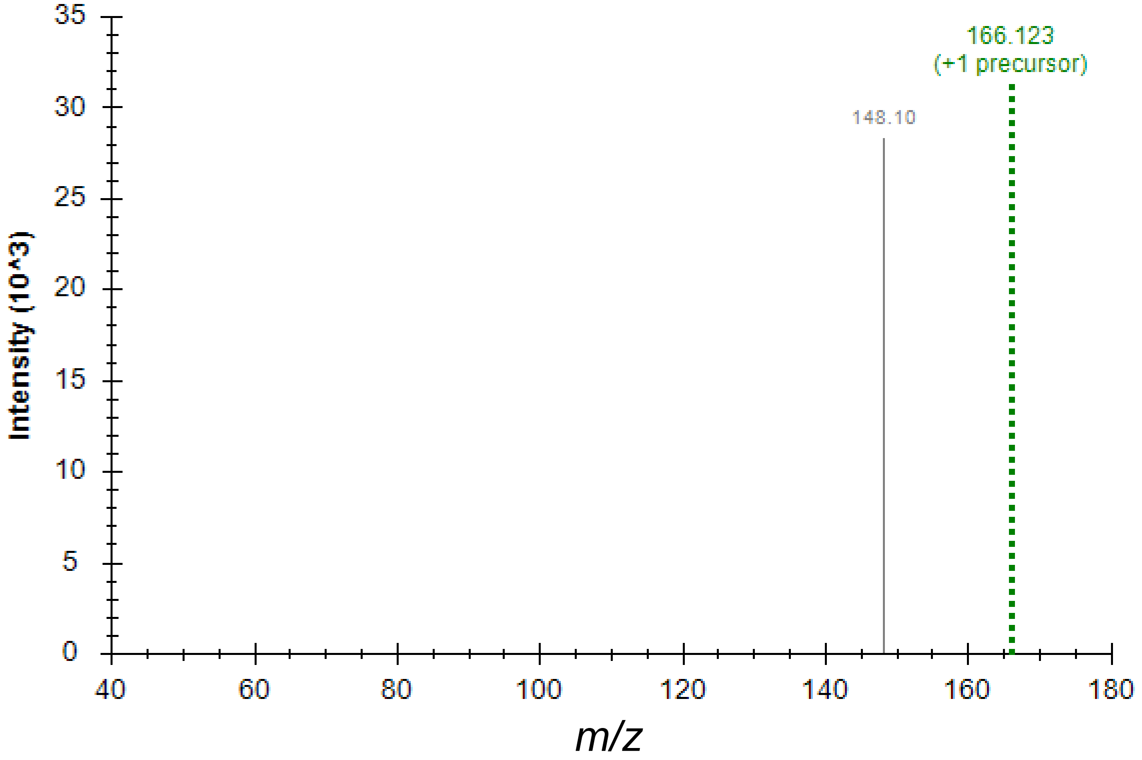

Norephedrine $[M+H]^+$

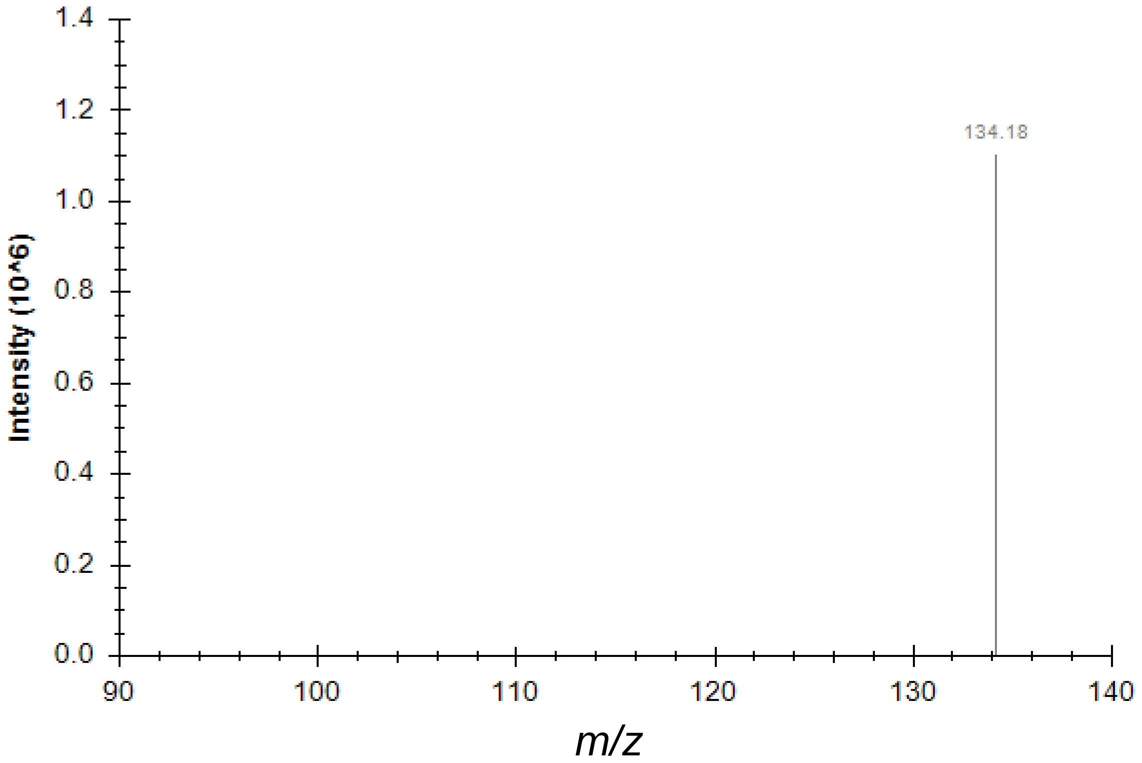

a) MS spectrum of the standard compounds

Pseudoephedrine [M+H]$^+$

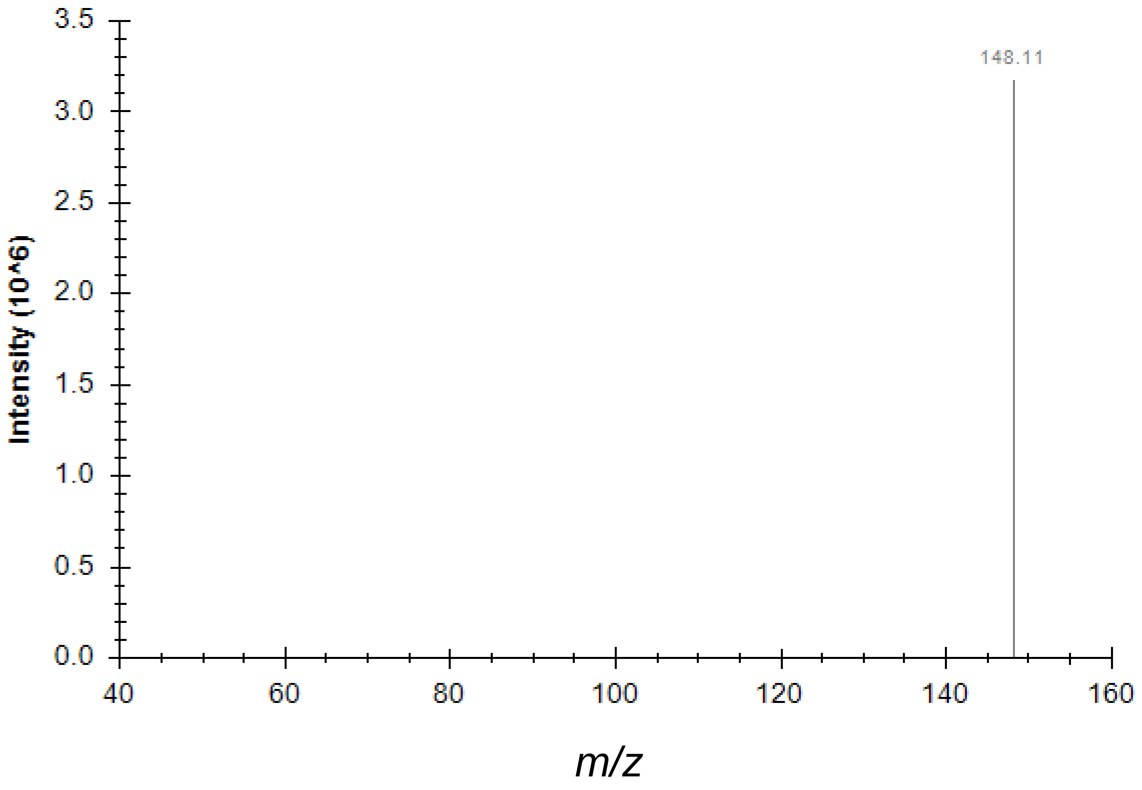

Methylephedrine [M+H]$^+$

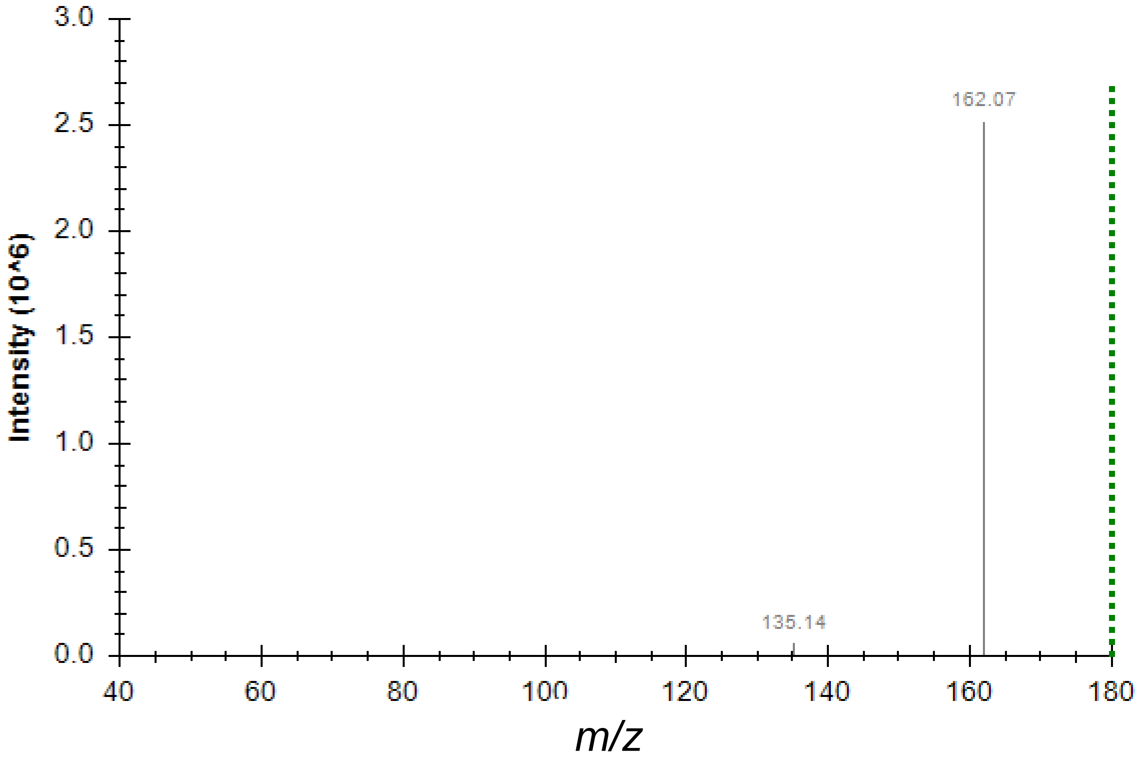

a) MS spectrum of the standard compounds

Amygdalin $[M+NH_4]^+$

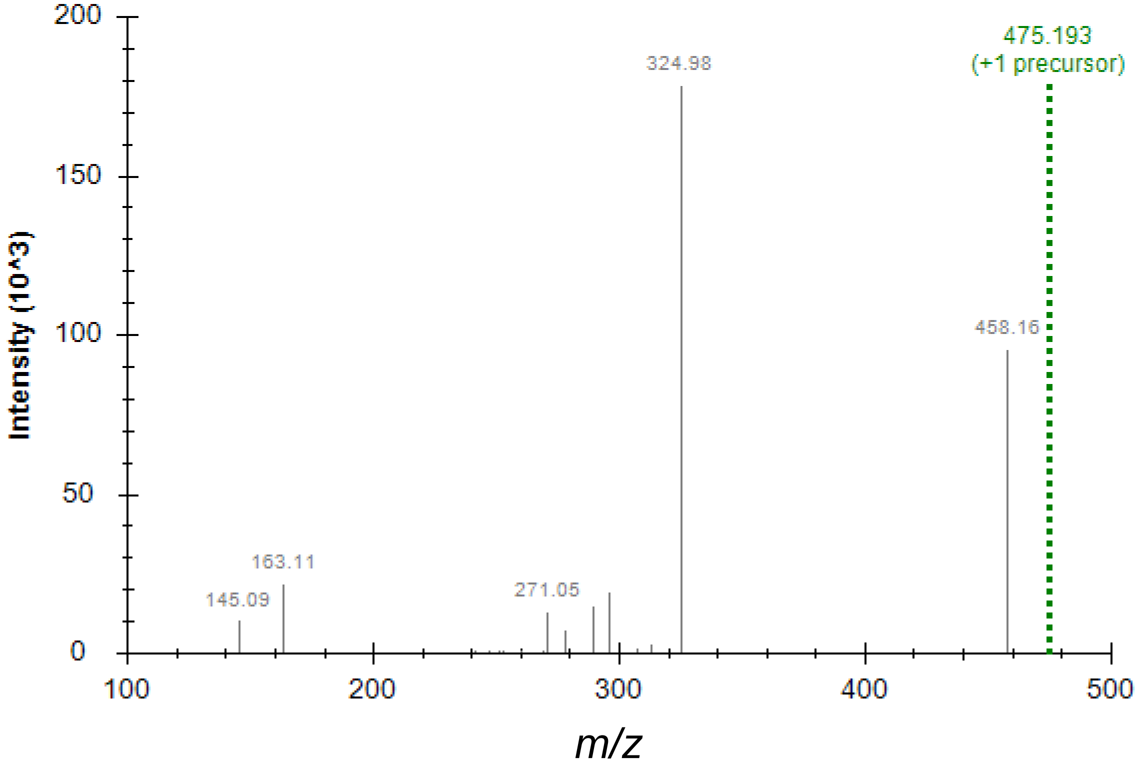

Amygdalin $[M+HCOO]^-$

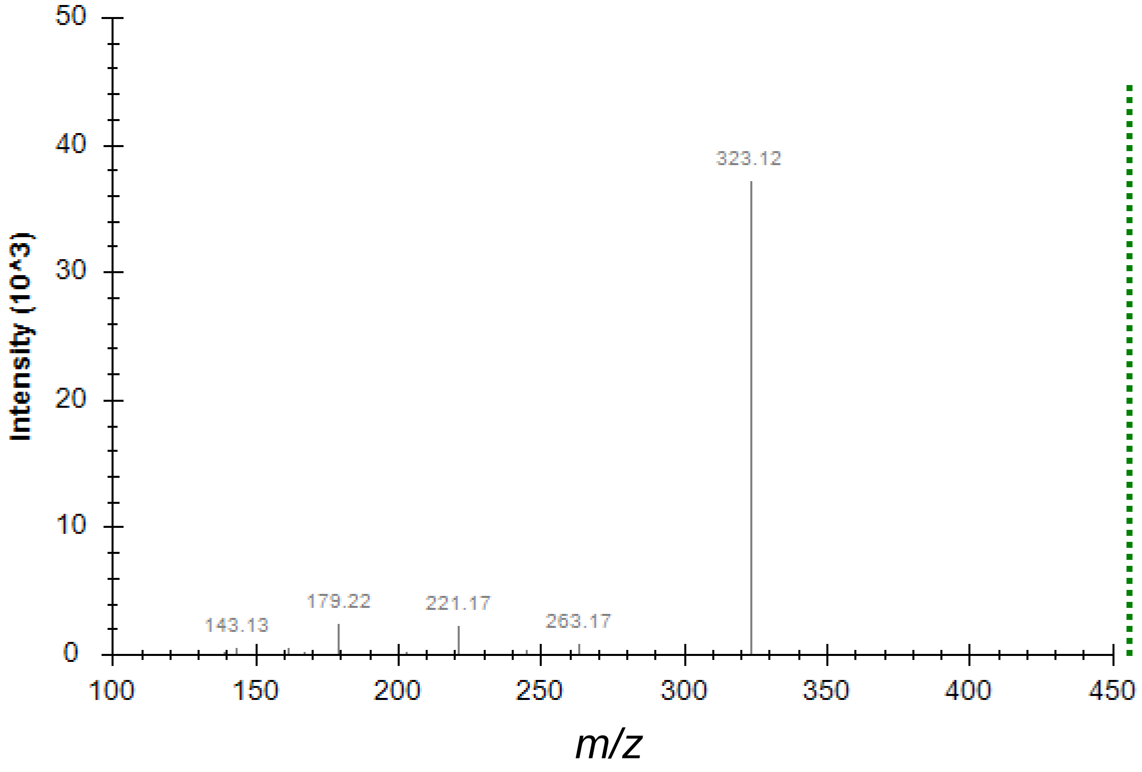

a) MS spectrum of the standard compounds

Prunasin [M+HCOO]$^-$

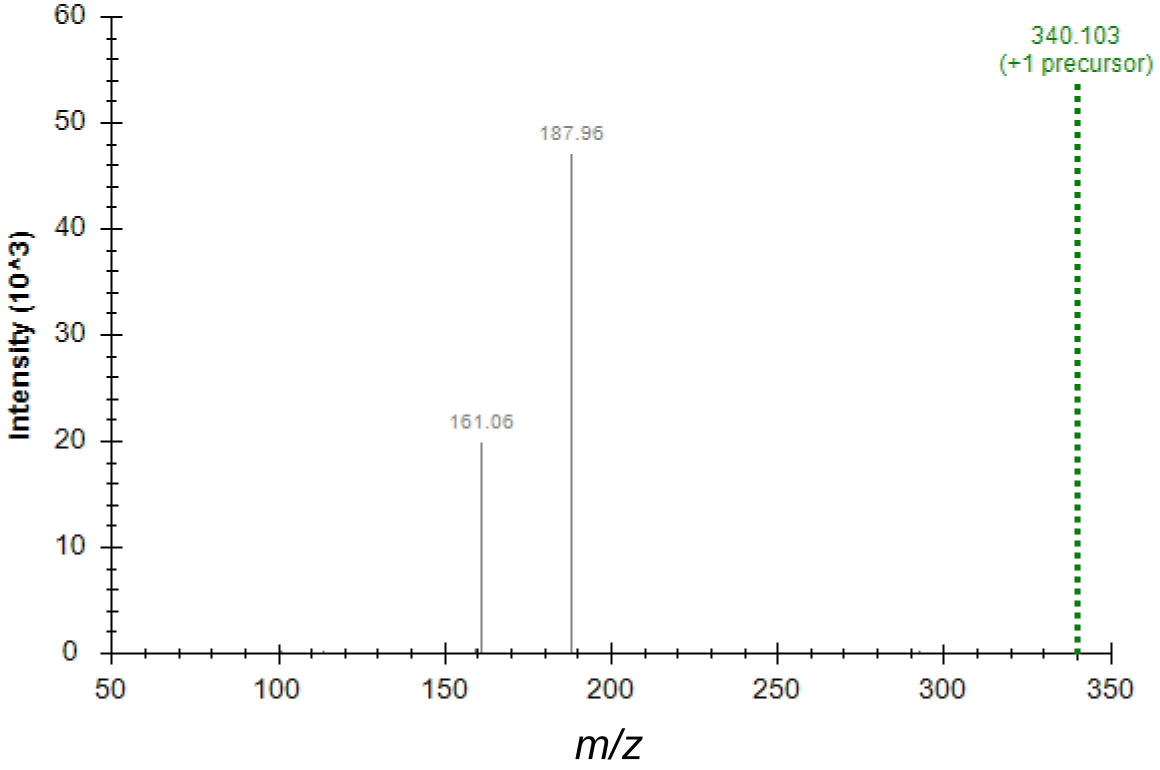

Liquiritin [M-H]$^-$

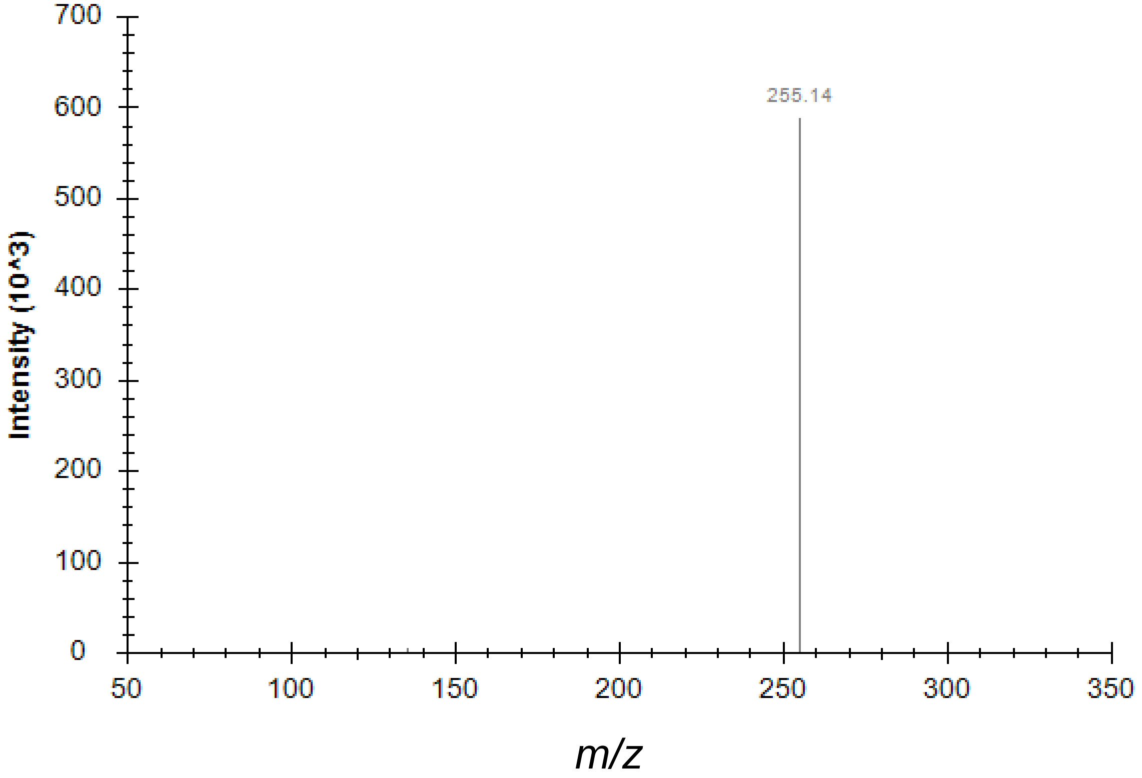

a) MS spectrum of the standard compounds

Isoliquiritin [M+H]$^+$

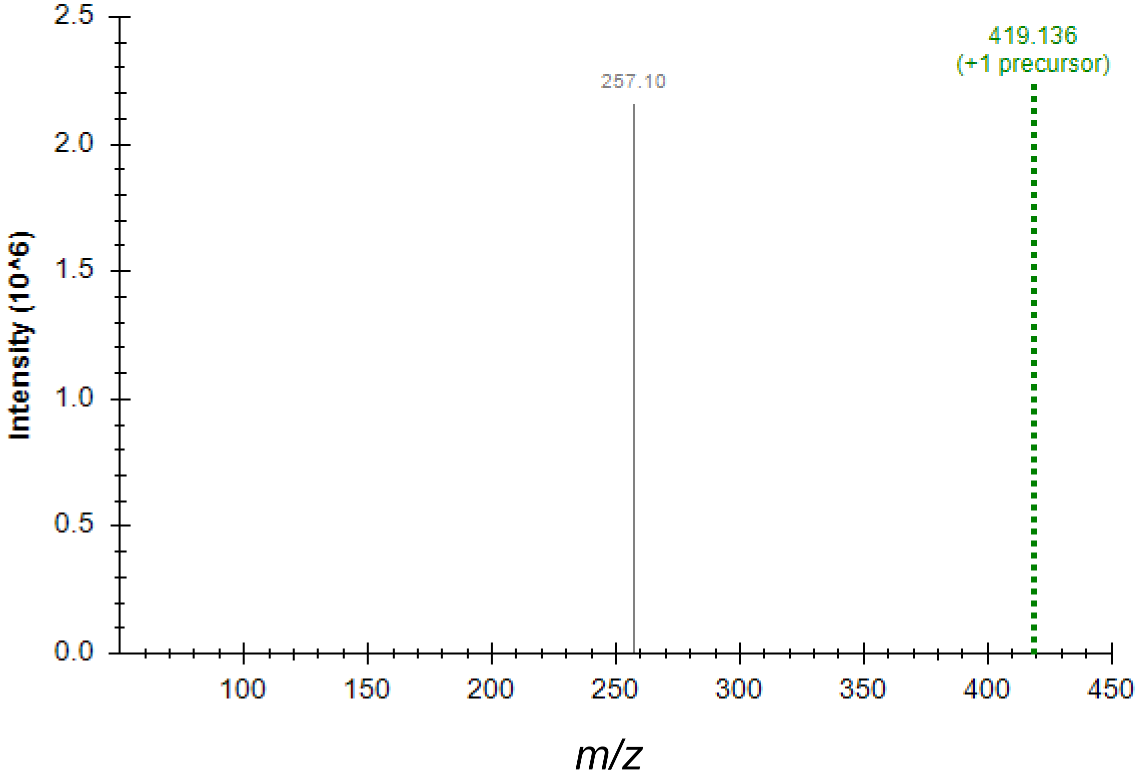

Isoliquiritin [M-H]$^-$

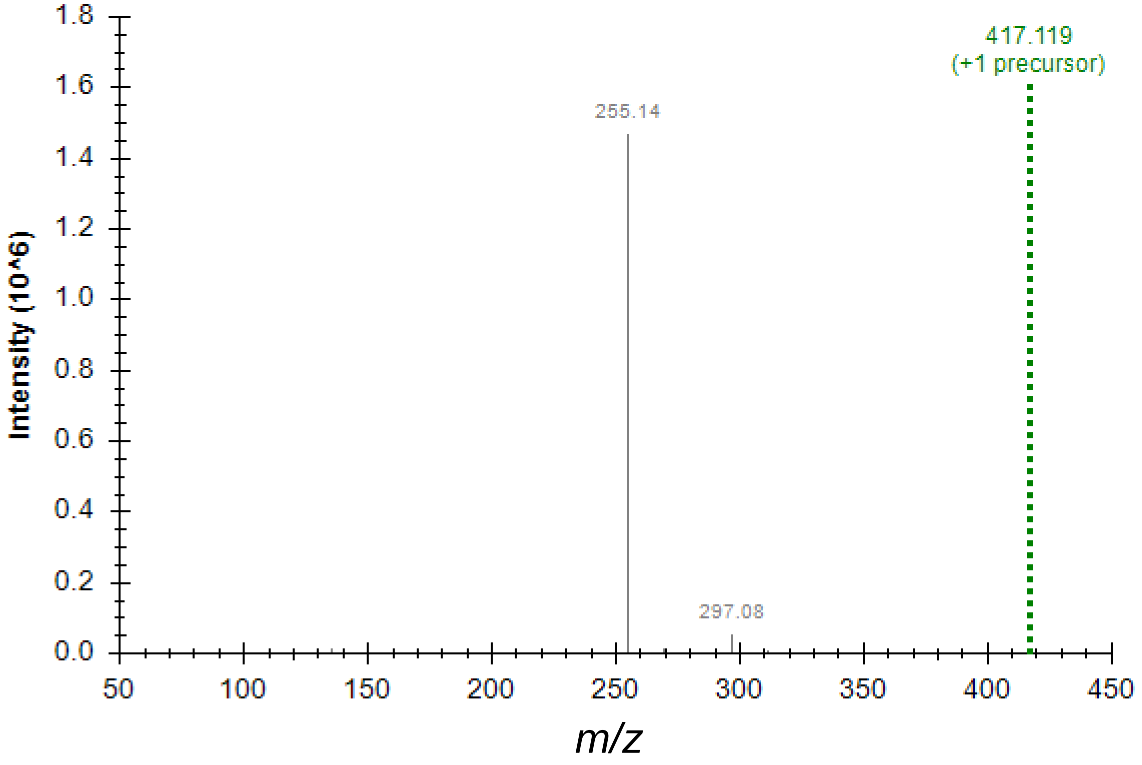

a) MS spectrum of the standard compoundsv

Liquiritigenin [M+H]$^+$

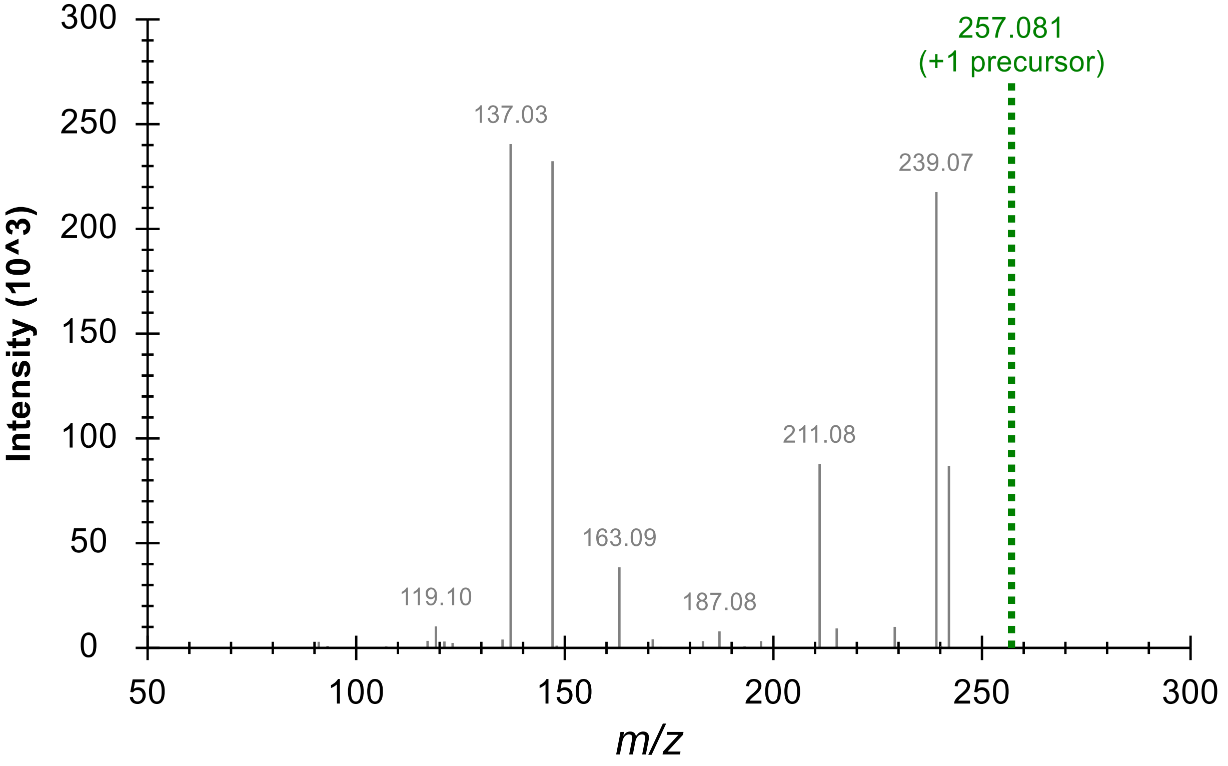

Liquiritigenin [M-H]$^-$

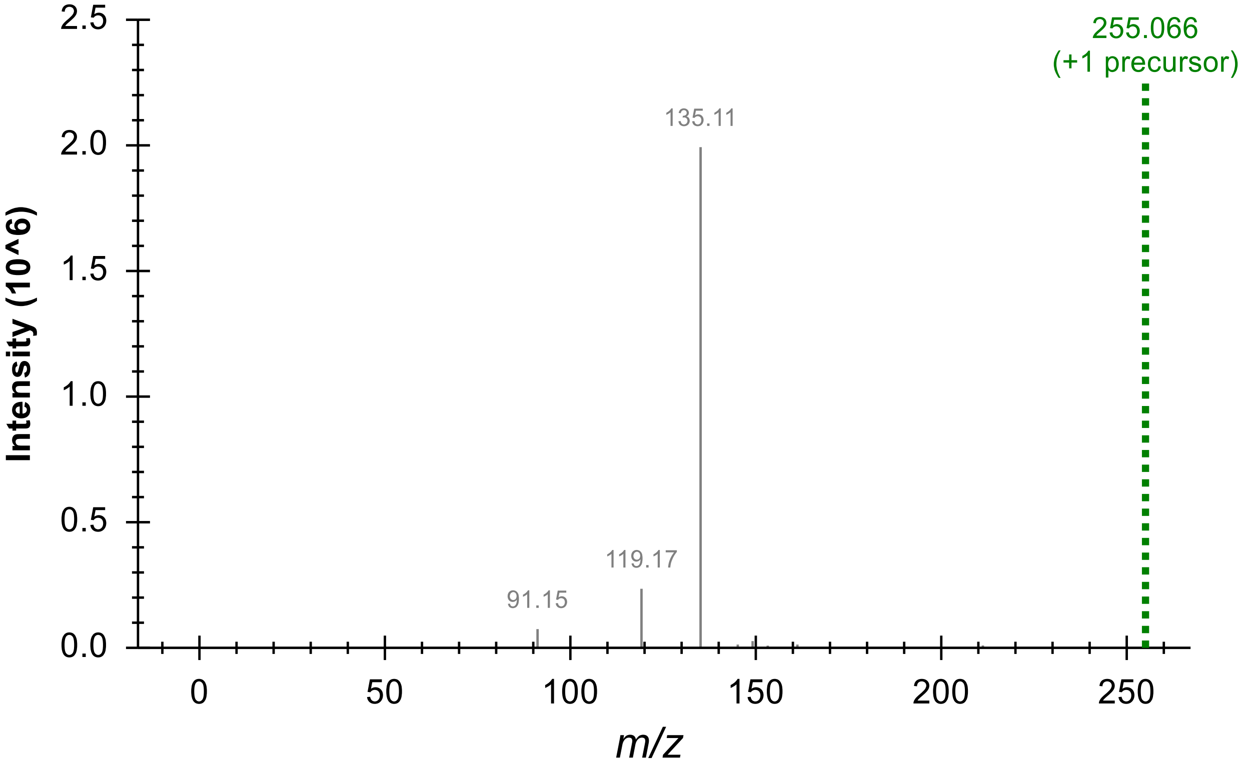

a) MS spectrum of the standard compounds

Isoliquiritigenin [M+H]+

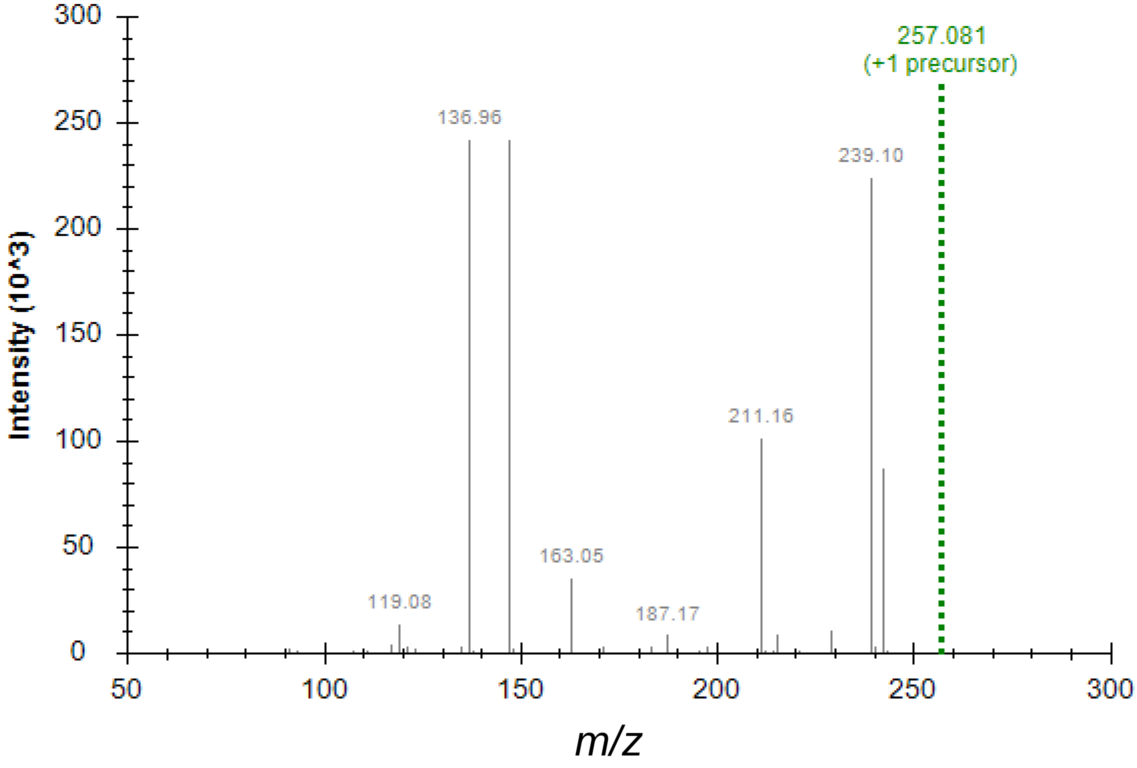

Isoliquiritigenin [M-H]-

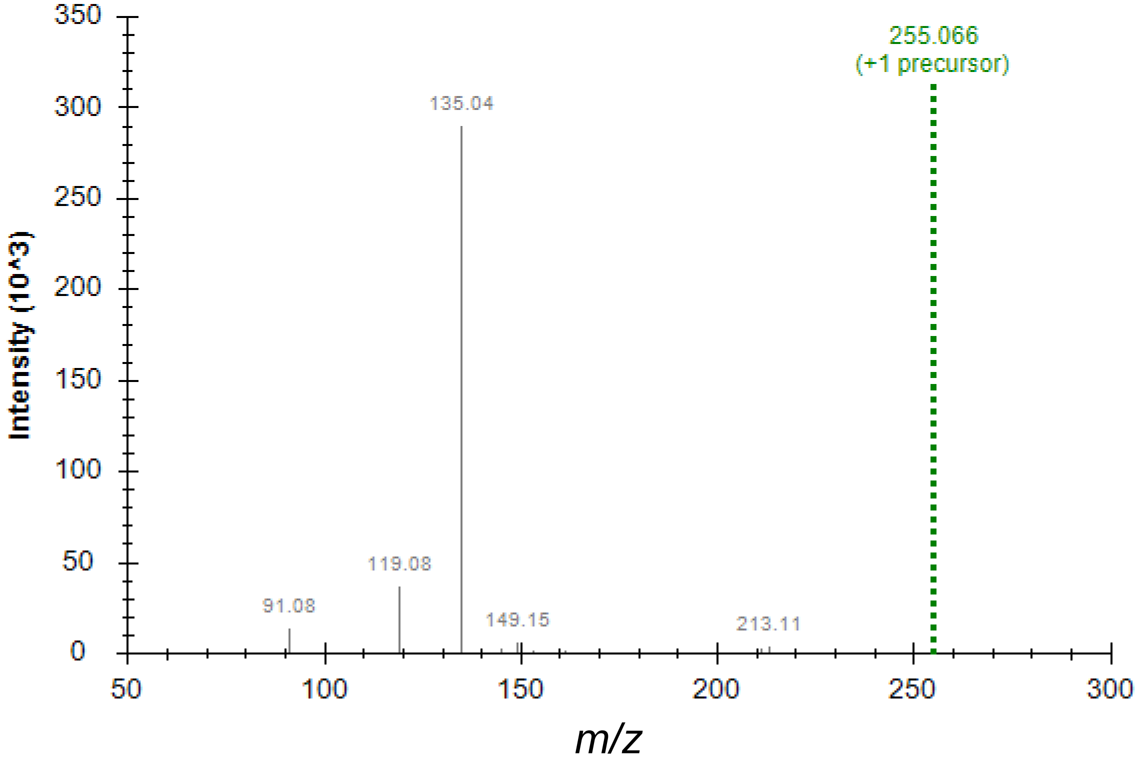

MS spectrum of the standard compounds

Liquiritigenin-4’-glucuronide [M+H]$^+$

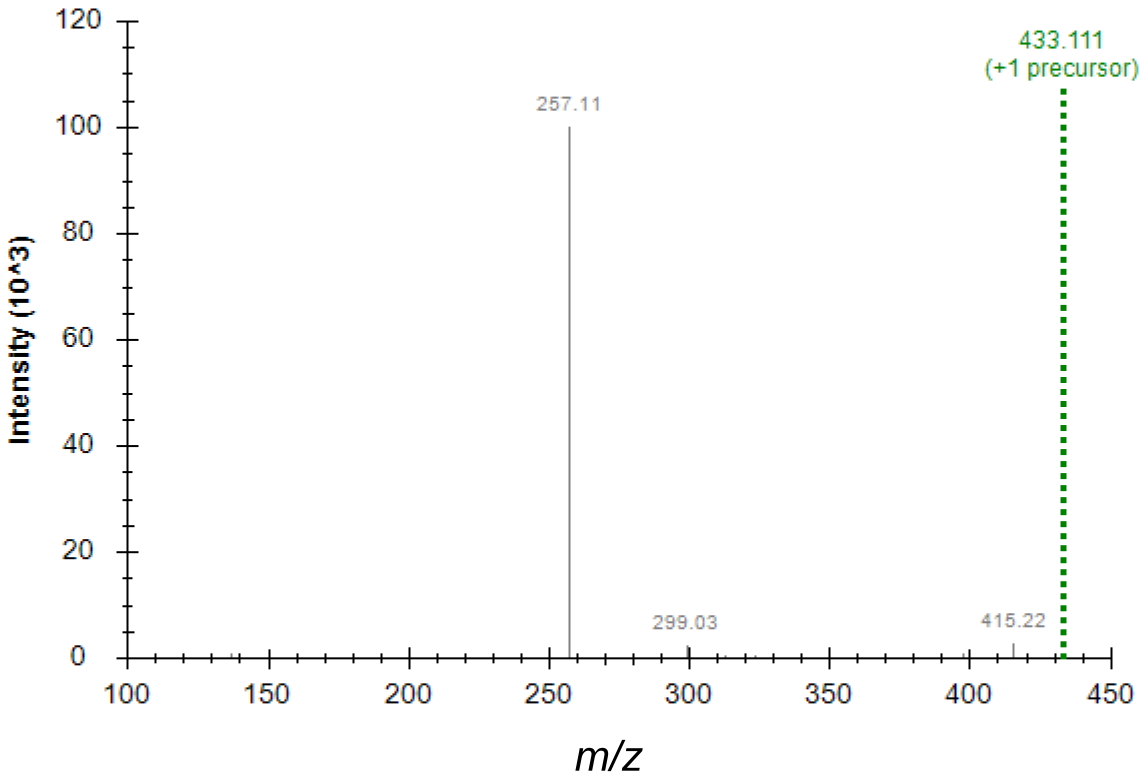

Liquiritigenin-4’-glucuronide [M-H]$^-$

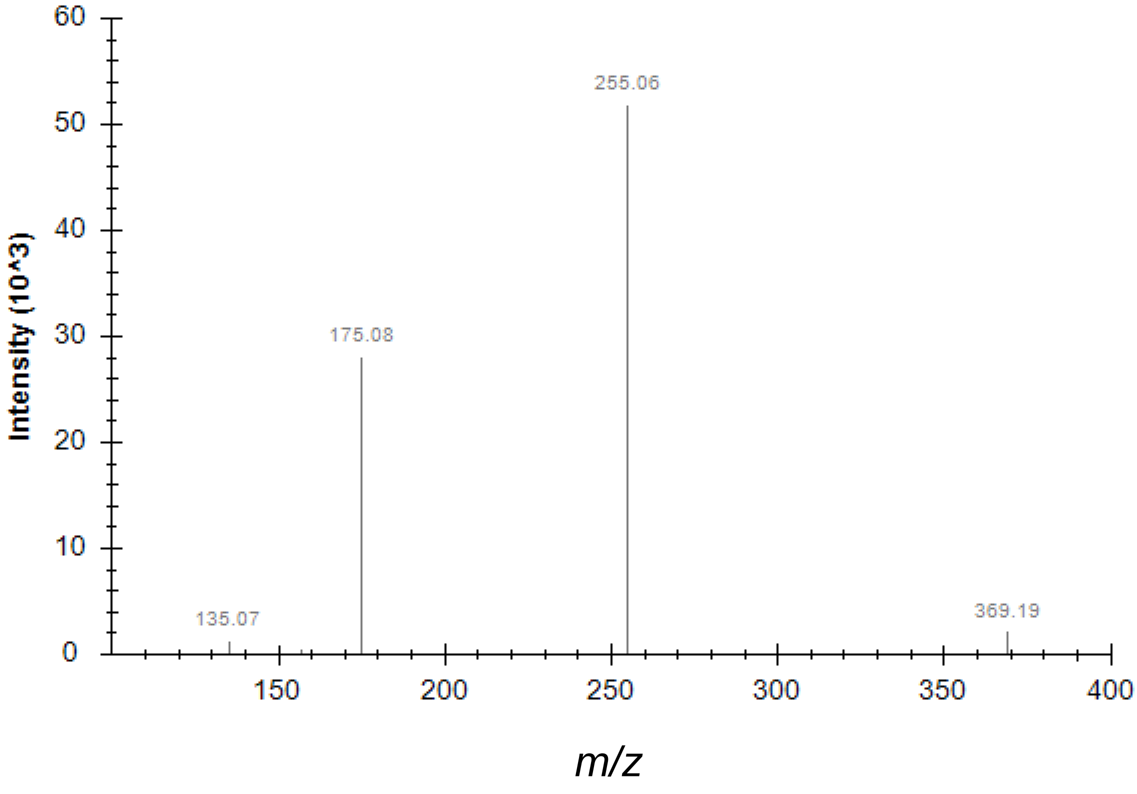

a) MS spectrum of the standard compounds

Liquiritigenin-7-glucuronide [M+H]$^+$

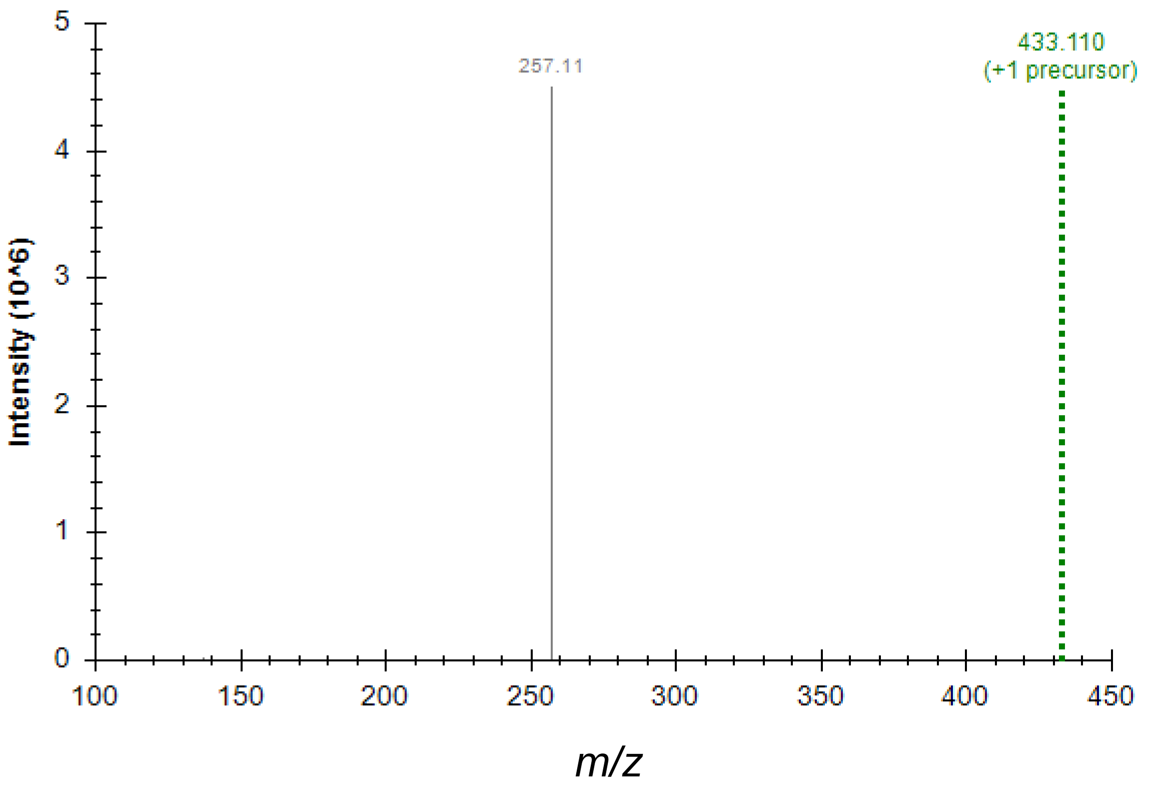

Liquiritigenin-7-glucuronide [M-H]$^-$

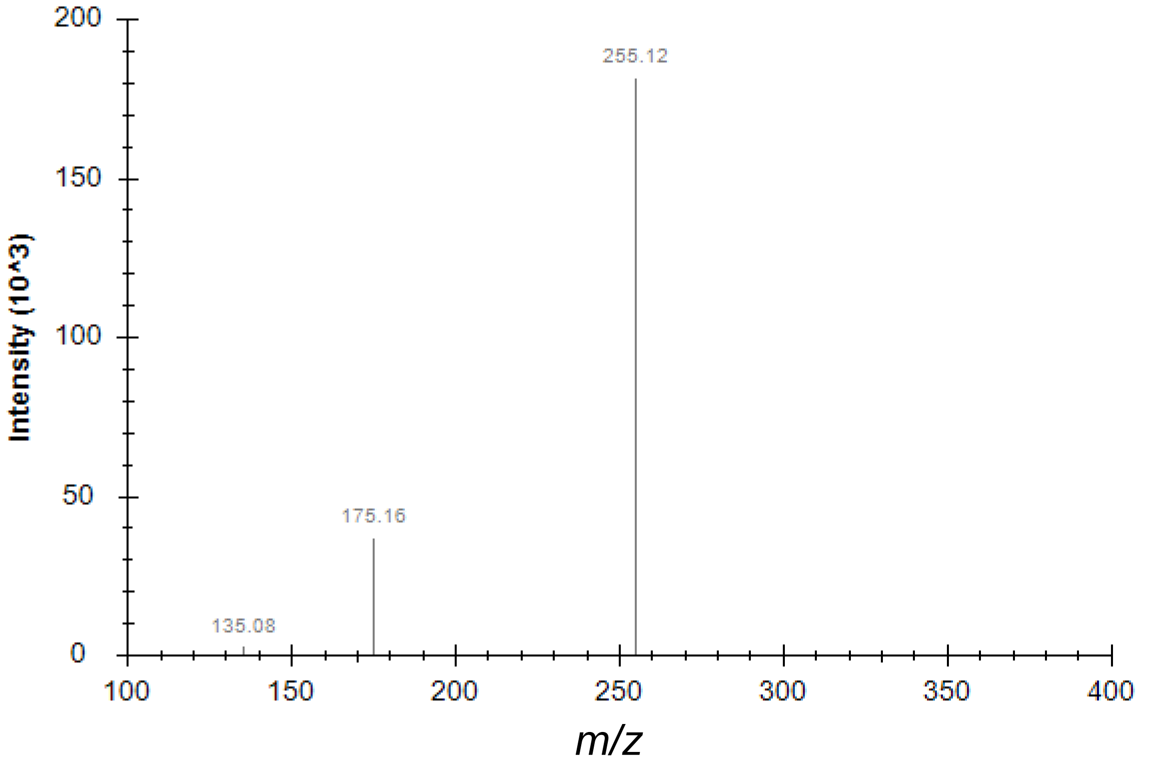

a) MS spectrum of the standard compounds

Liquiritigenin-2-glucuronide [M+H]+

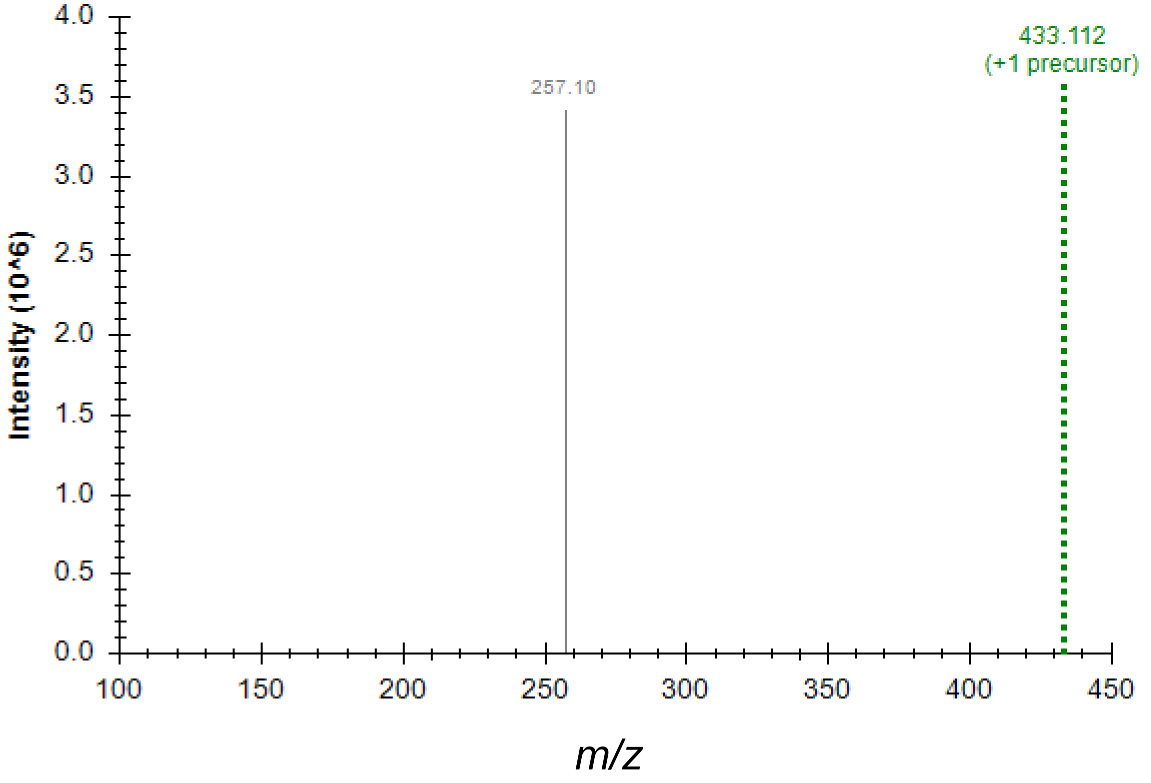

Liquiritigenin-2-glucuronide [M-H]-

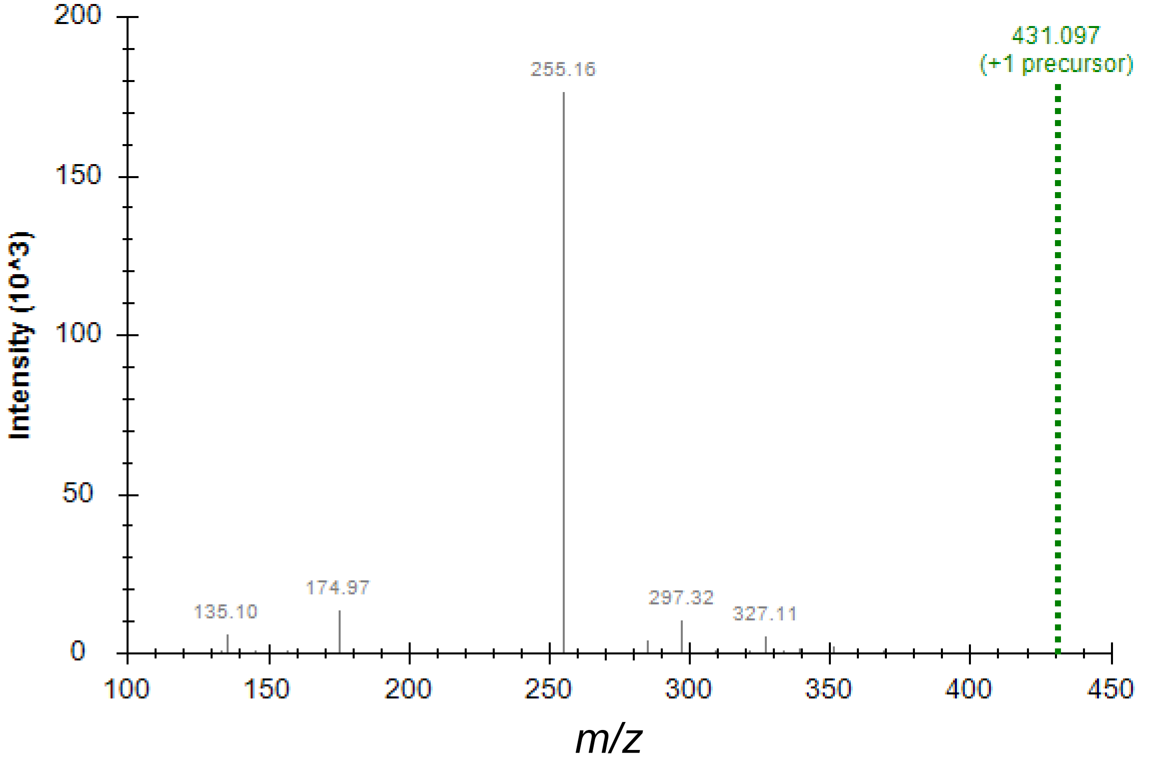

a) MS spectrum of the standard compounds

Isoliquiritigenin-4-glucuronide [M+H]$^{+}$

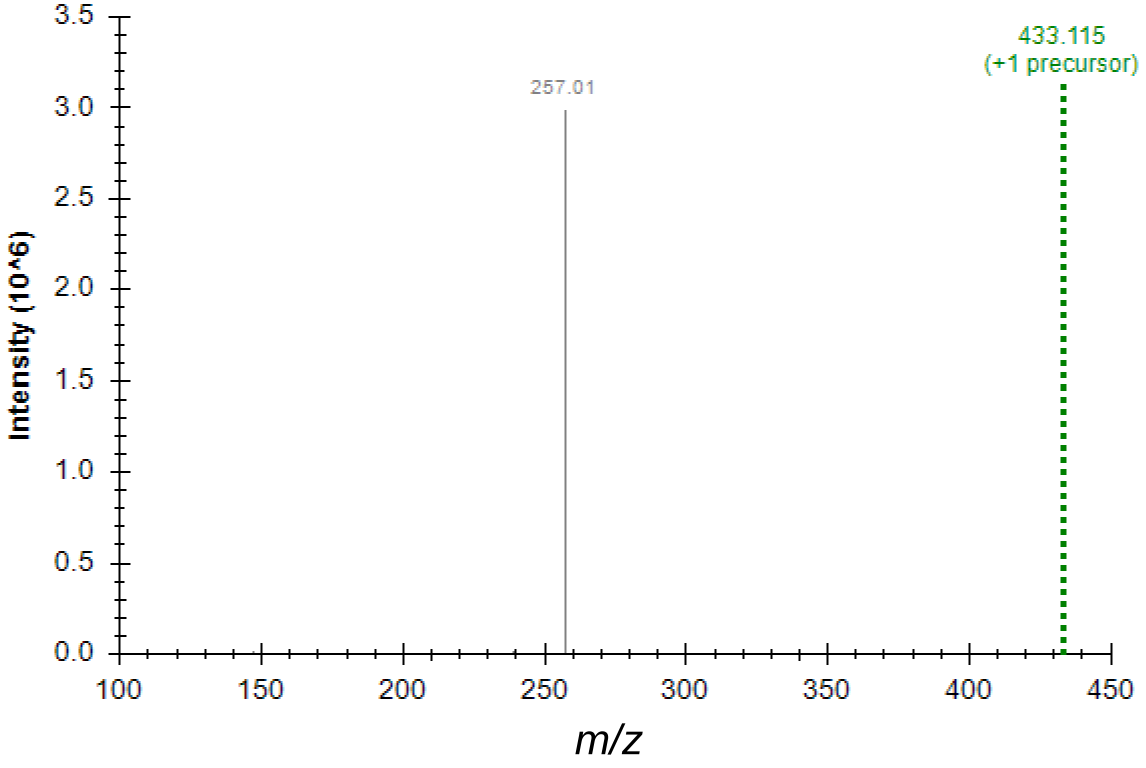

Isoliquiritigenin-4-glucuronide [M-H]$^{-}$

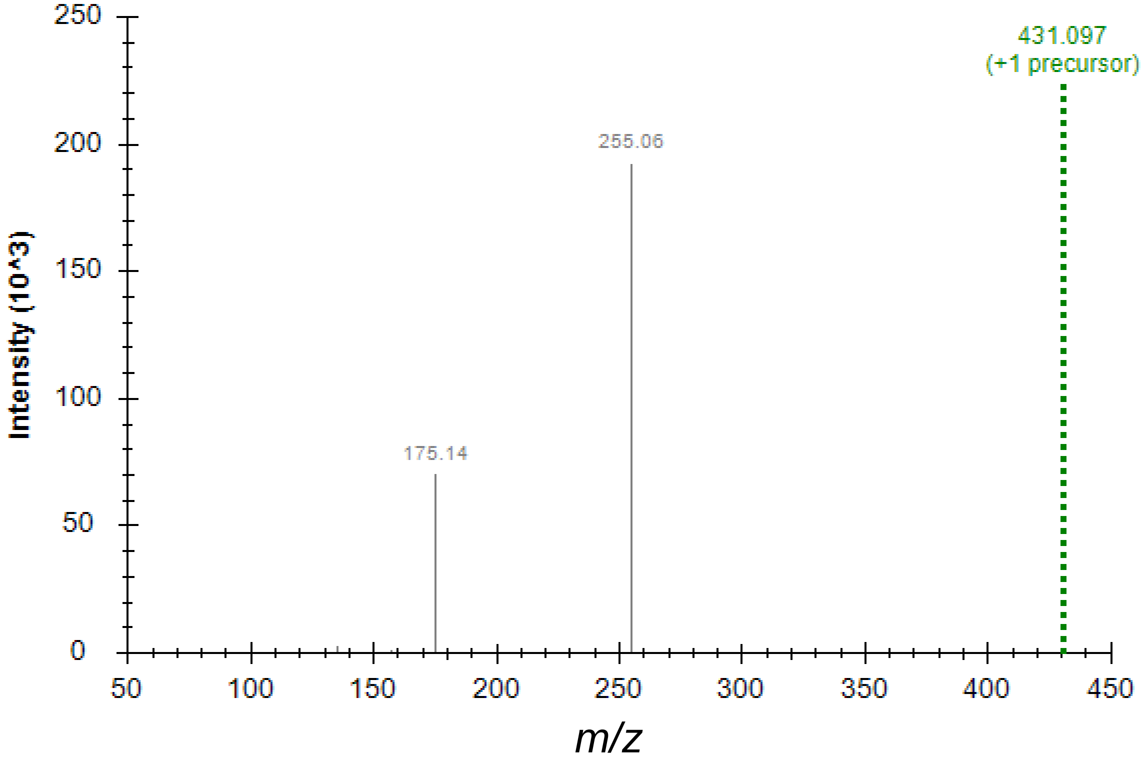

a) MS spectrum of the standard compounds

Isoliquiritigenin-4’-glucuronide [M+H]+

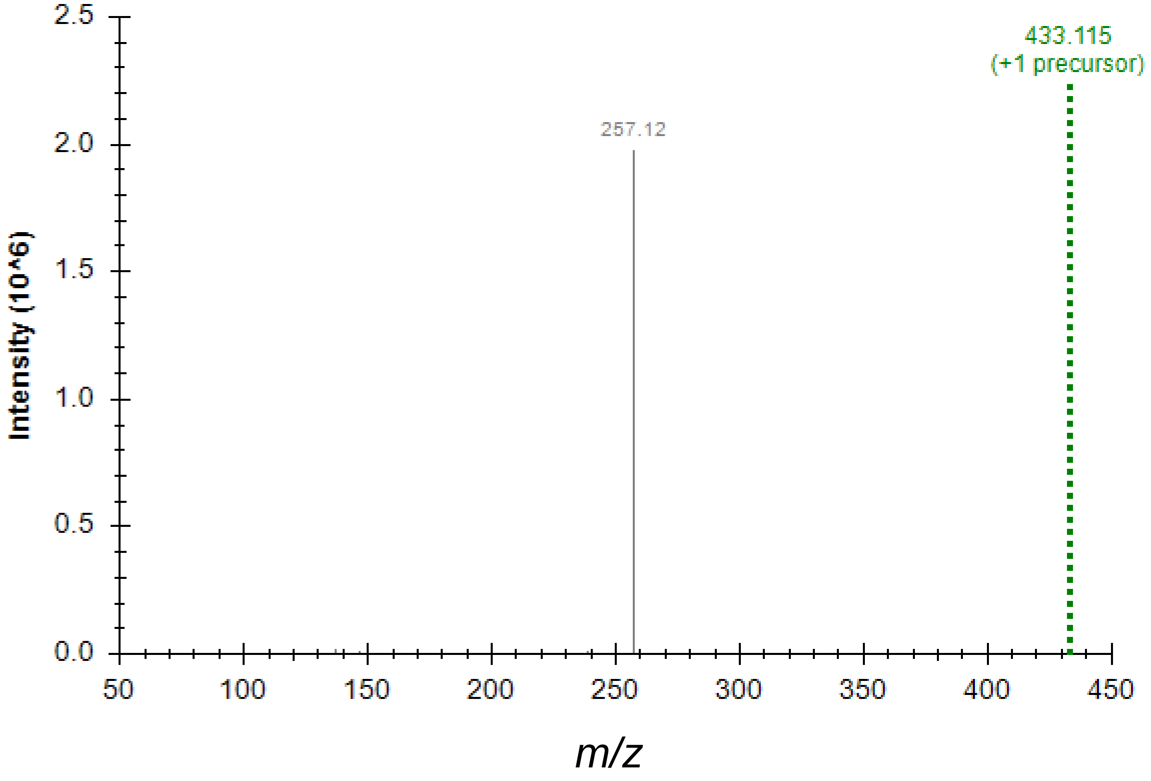

Isoliquiritigenin-4’-glucuronide [M-H]-

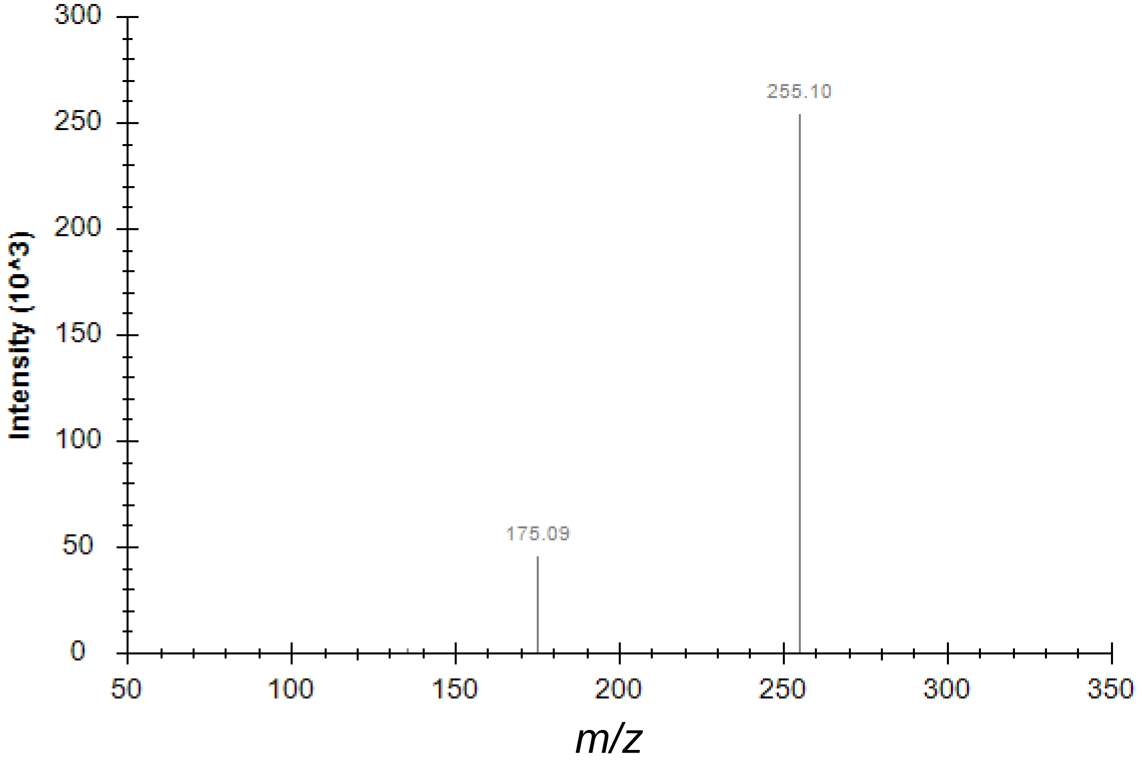

a) MS spectrum of the standard compounds

Glycyrrhizinic acid [M-2Hexose+H]$^+$

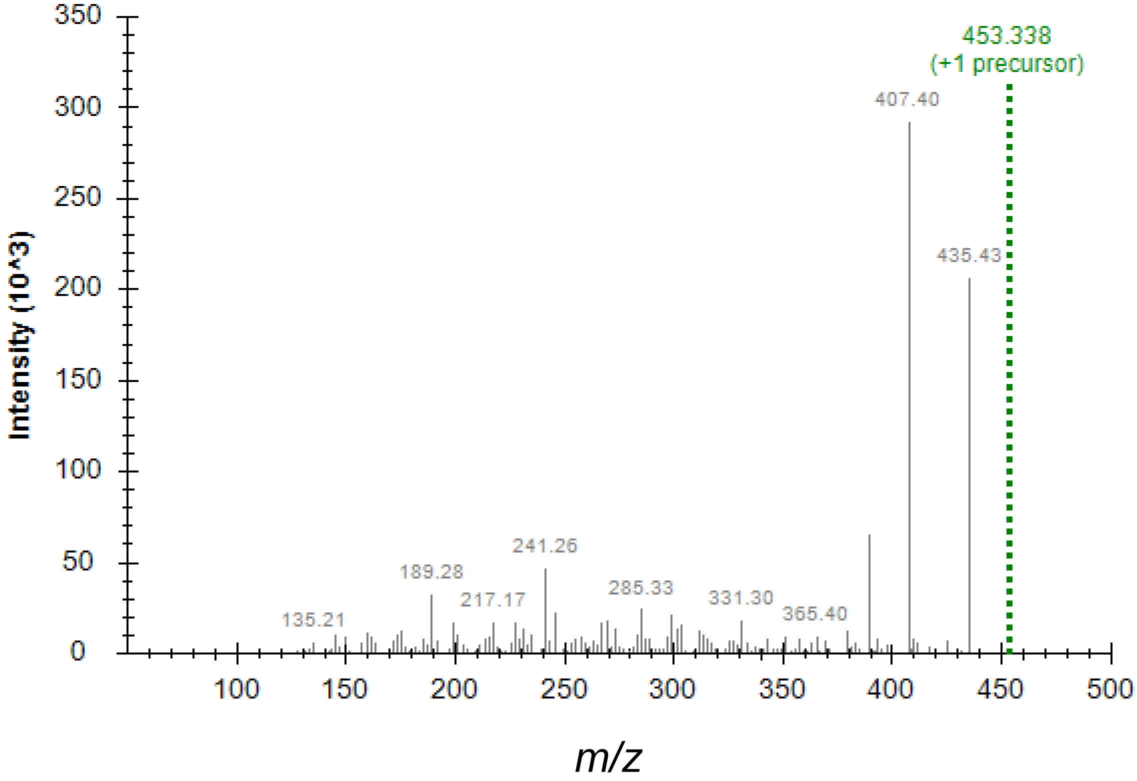

Glycyrrhizinic acid [M-H]$^-$

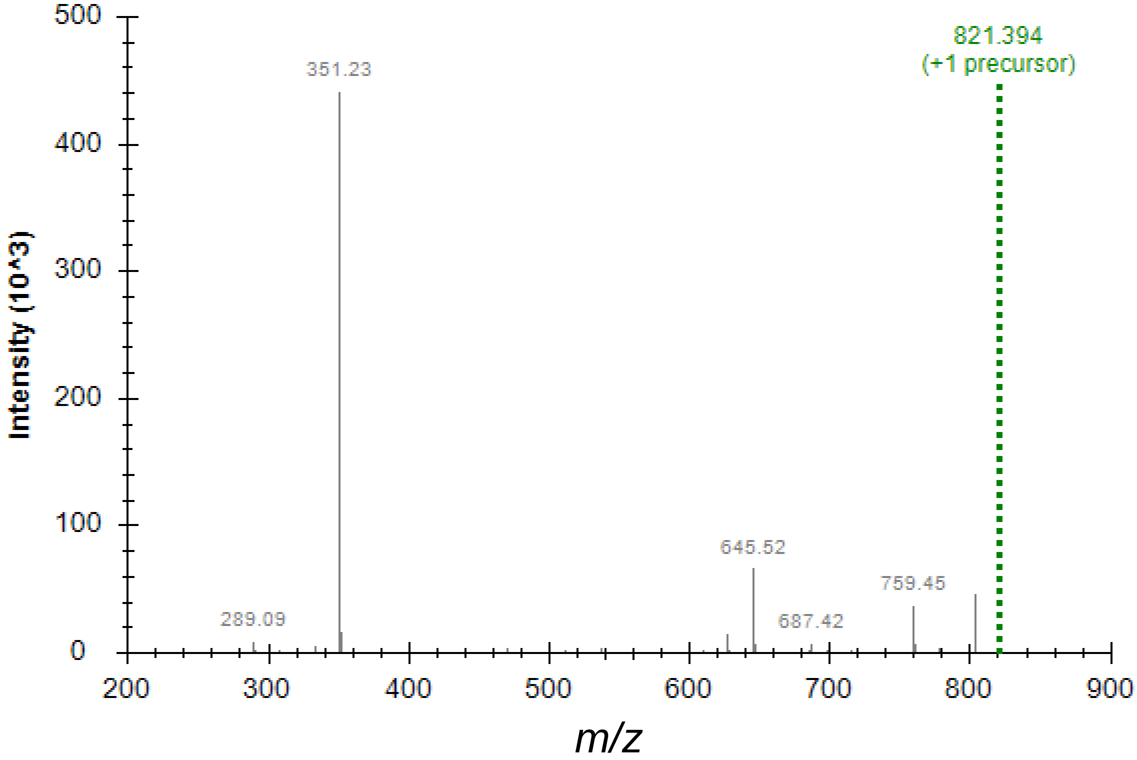

a) MS spectrum of the standard compounds

Glycyrrhetinic acid [M+H]$^+$

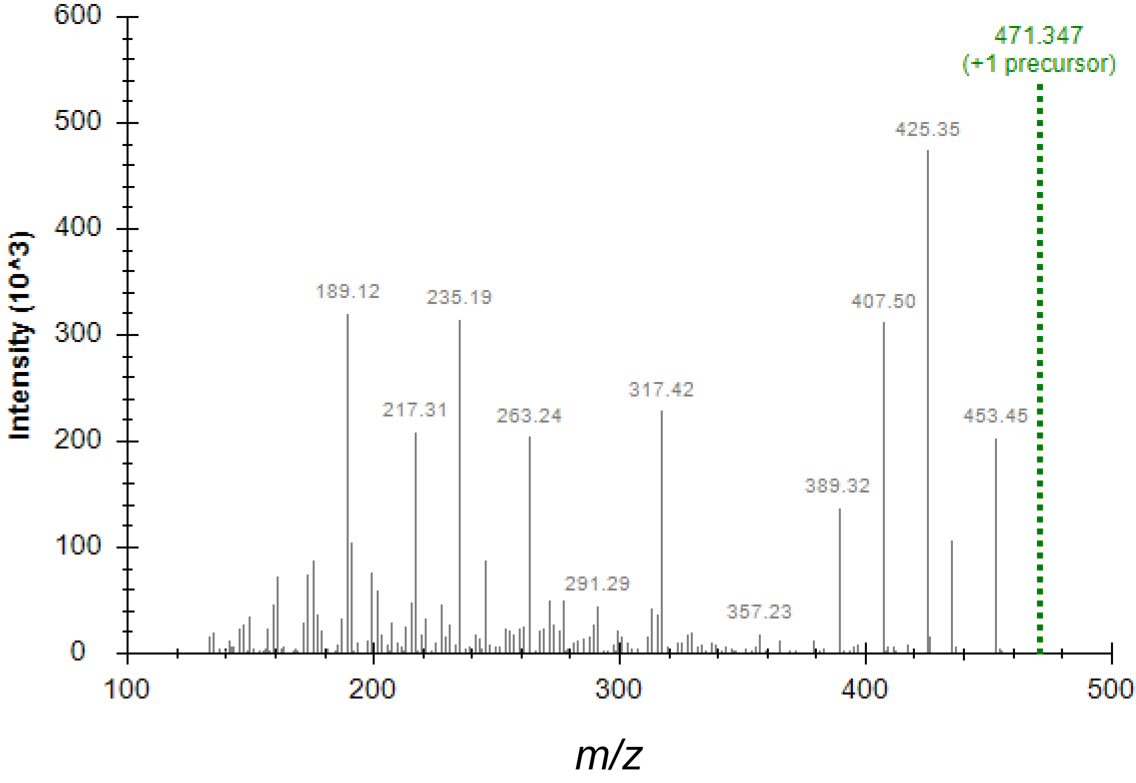

Glycyrrhetinic acid [M-H]$^-$

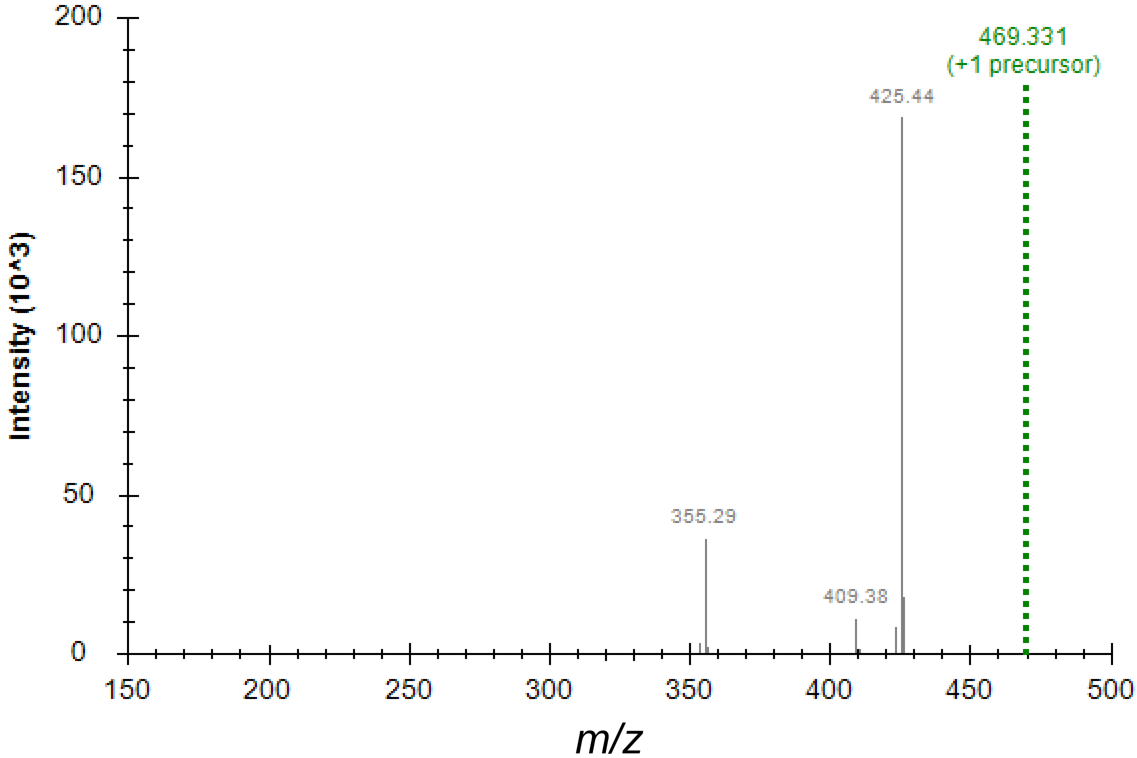

a) MS spectrum of the standard compounds

4-Hydroxycoumarin [M+H]$^{+}$

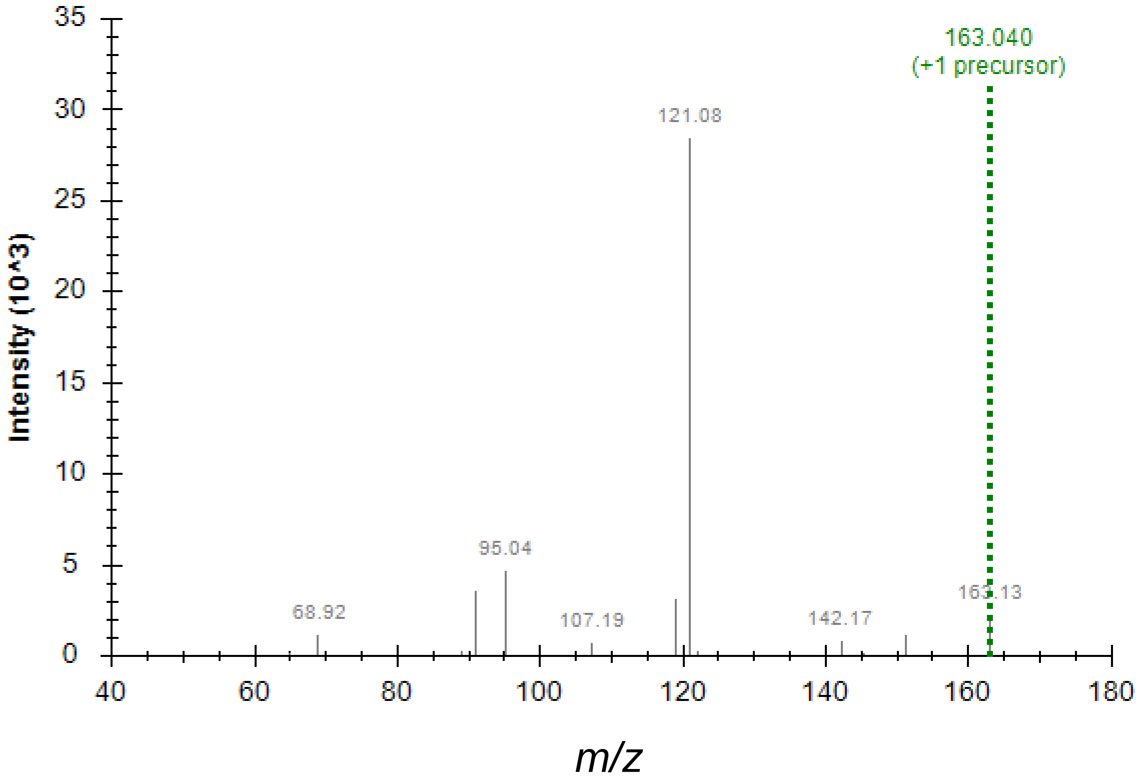

4-Hydroxycoumarin [M-H]$^{-}$

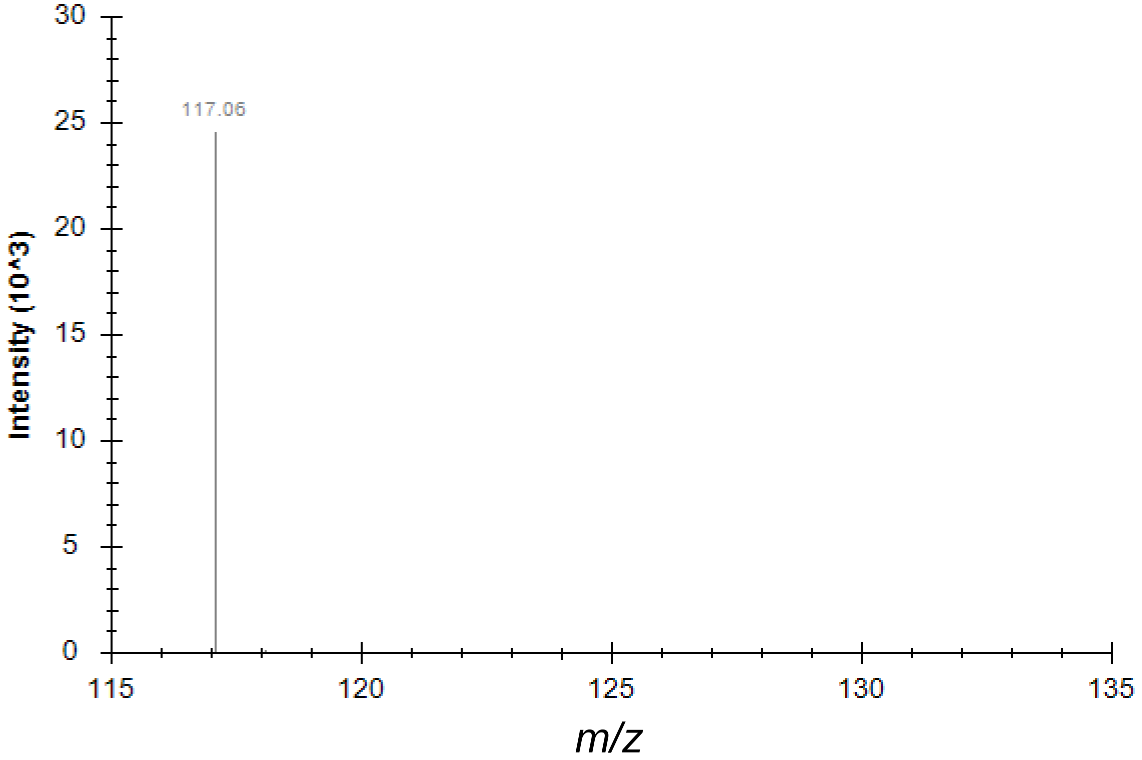

a) MS spectrum of the standard compounds

6-Hydroxycoumarin [M+H]+

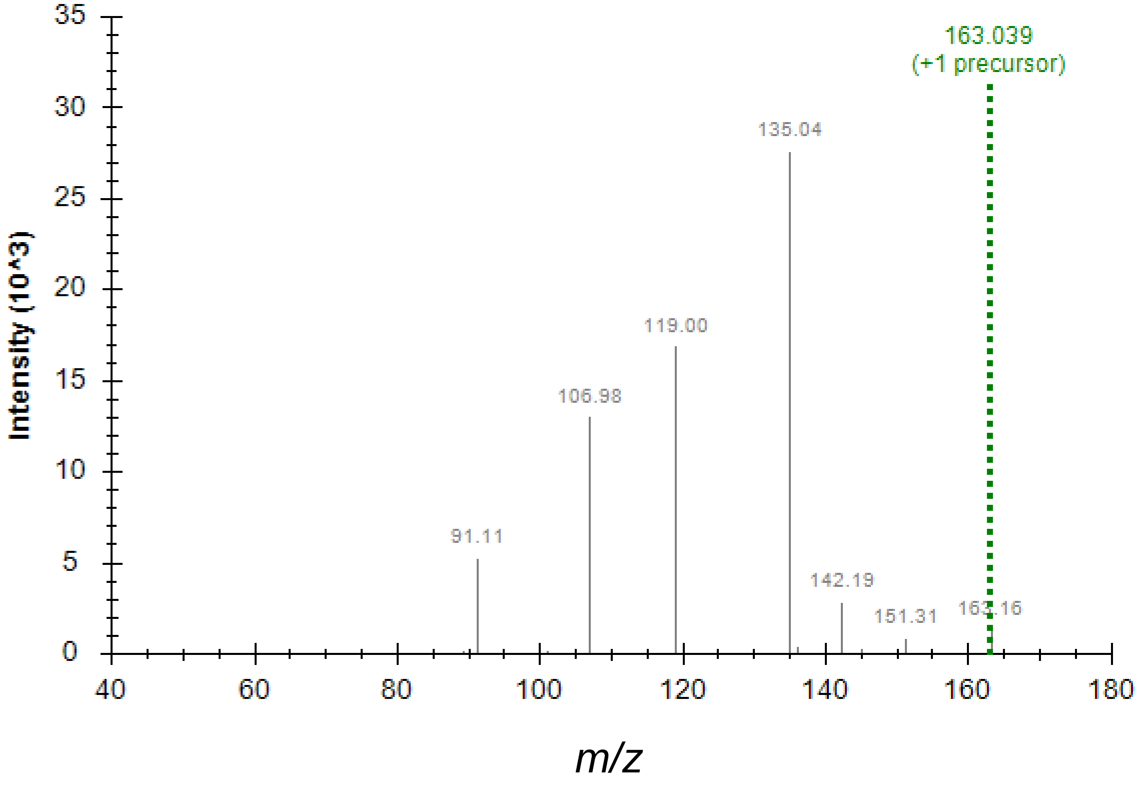

6-Hydroxycoumarin [M-H]-

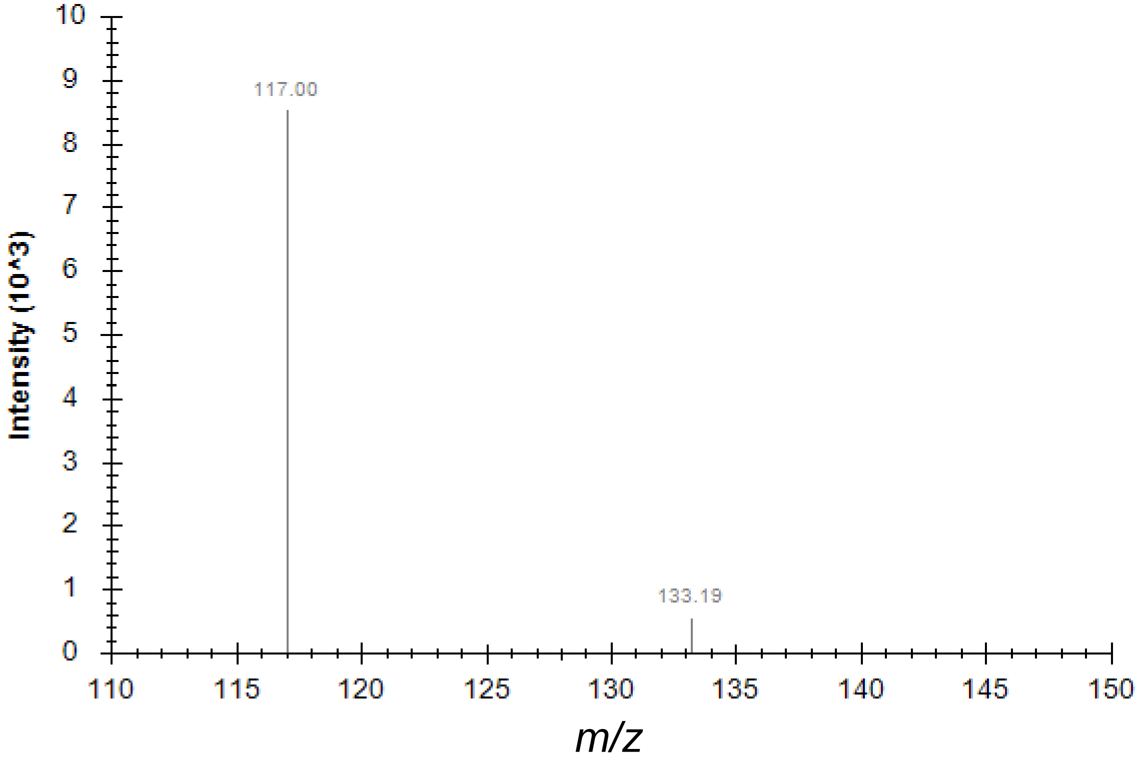

a) MS spectrum of the standard compounds

7-Hydroxycoumarin $[M+H]^+$

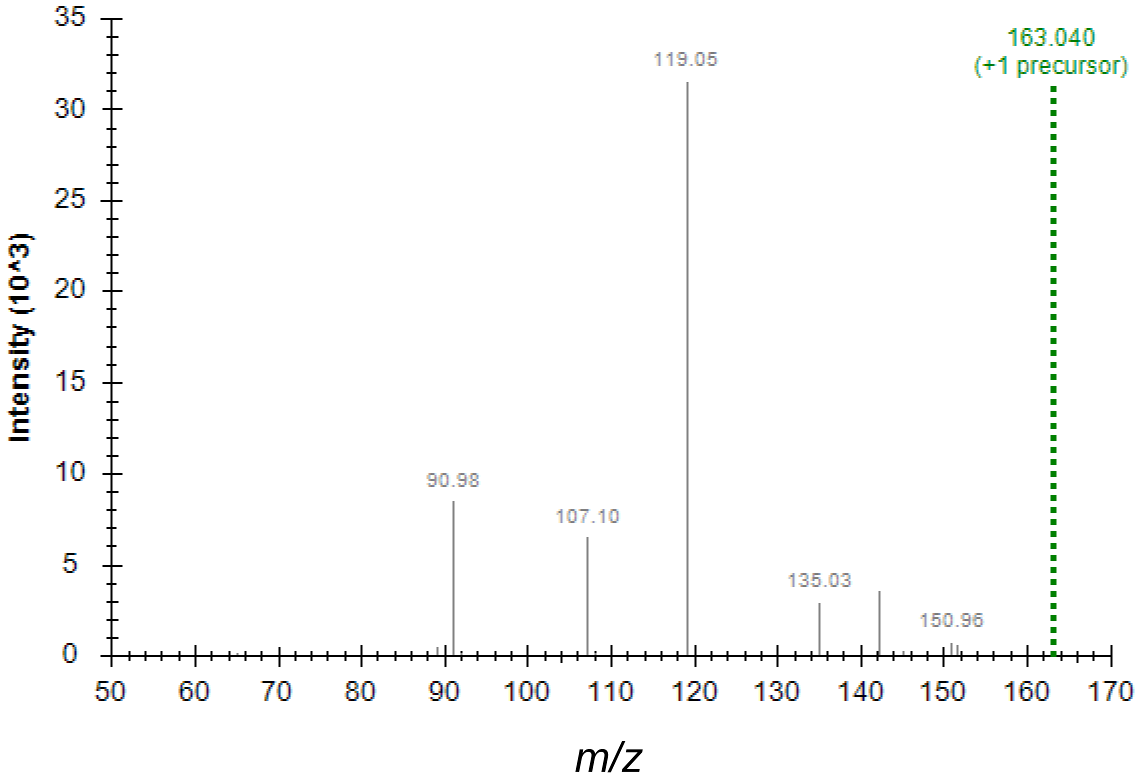

7-Hydroxycoumarin $[M-H]^-$

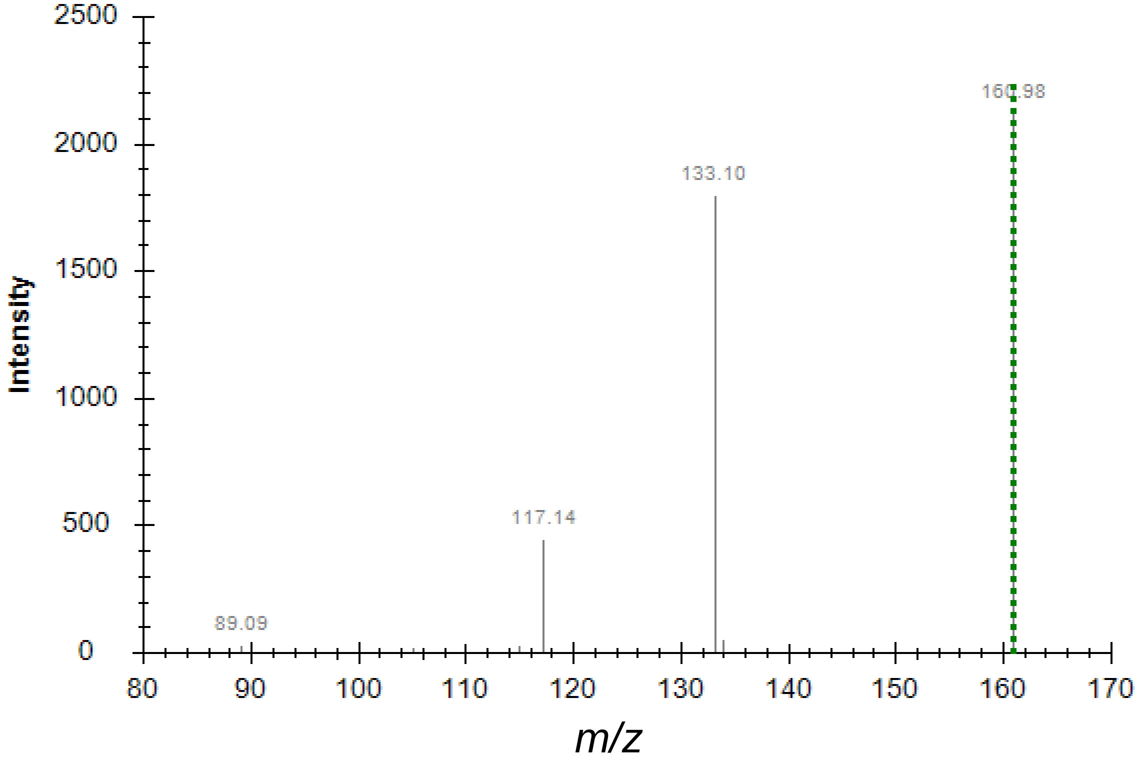

a) MS spectrum of the standard compounds

7-Hydroxycoumarin-glucuronide [M+H]⁺

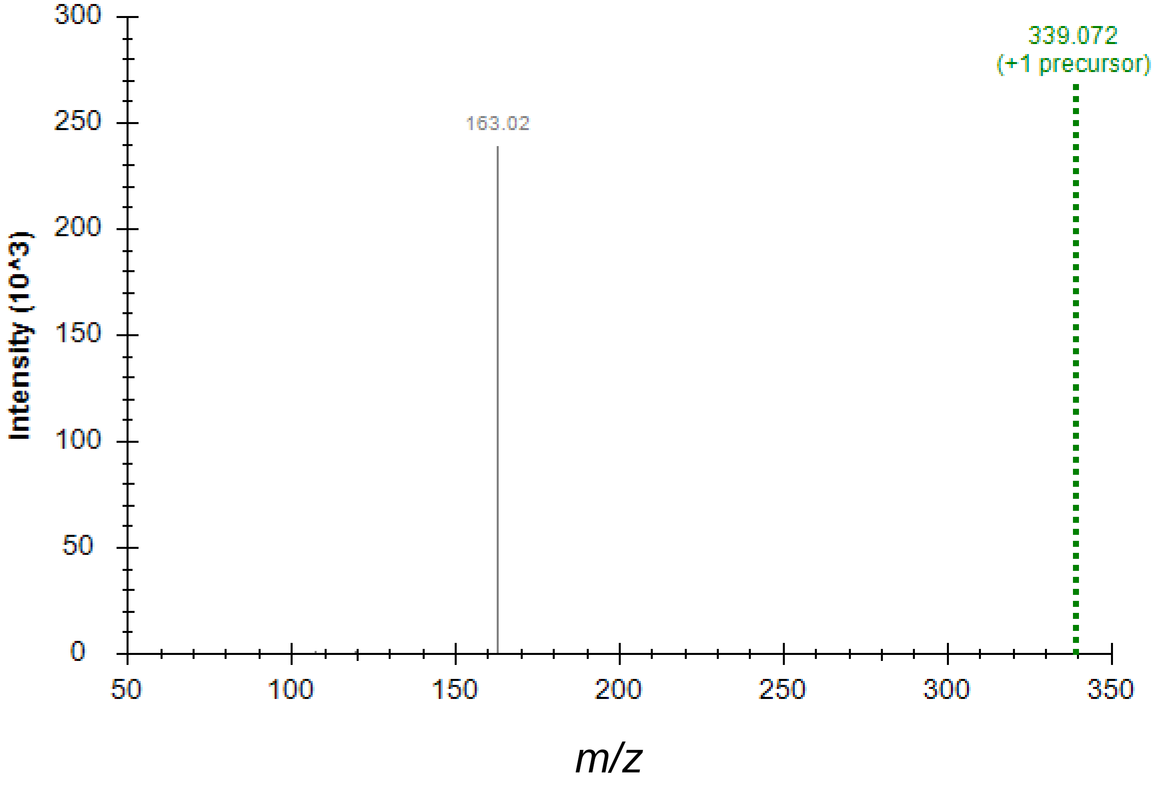

7-Hydroxycoumarin-glucuronide [M-H]⁻

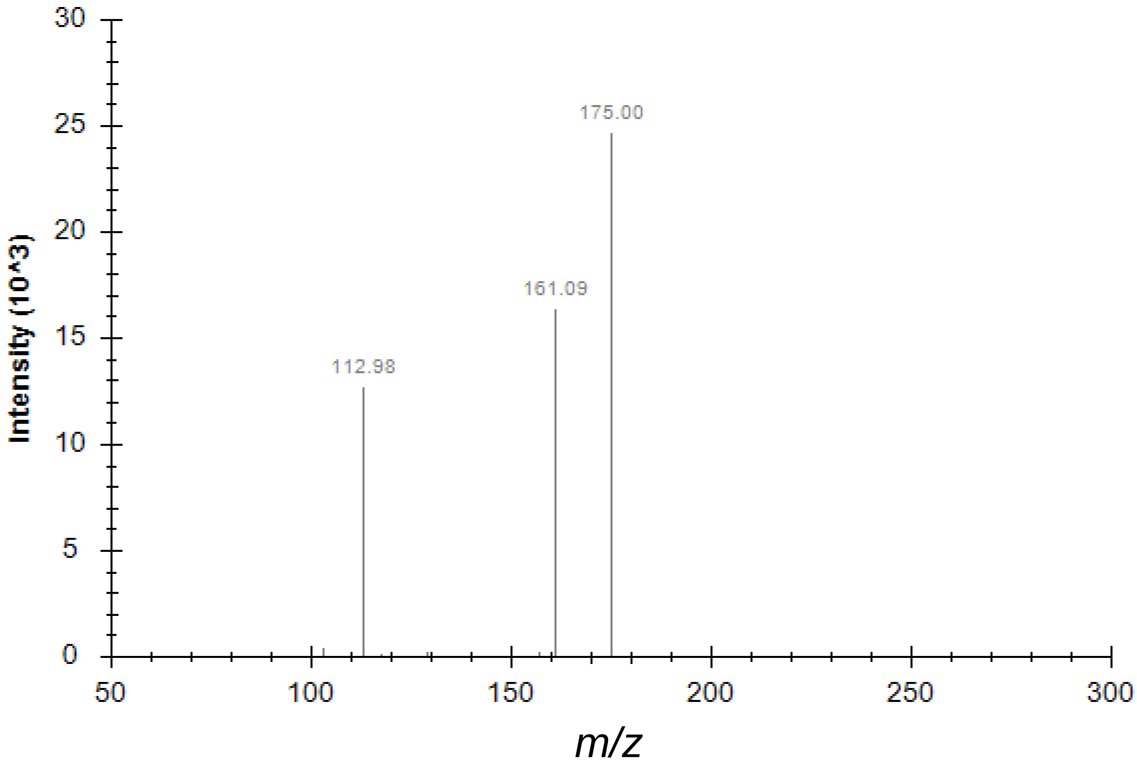

a) MS spectrum of the standard compounds

7-Hydroxycoumarin-sulfate $[M-HSO_3]^-$

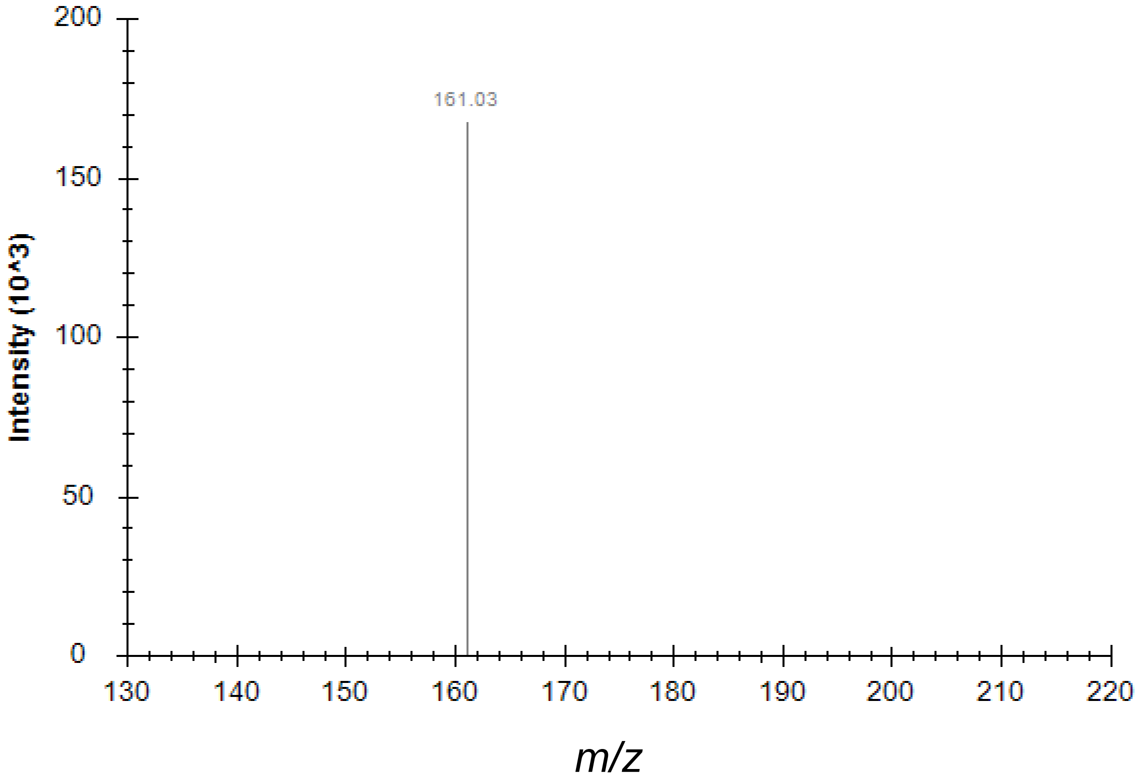

Indoxyl sulfate $[M-H]^-$

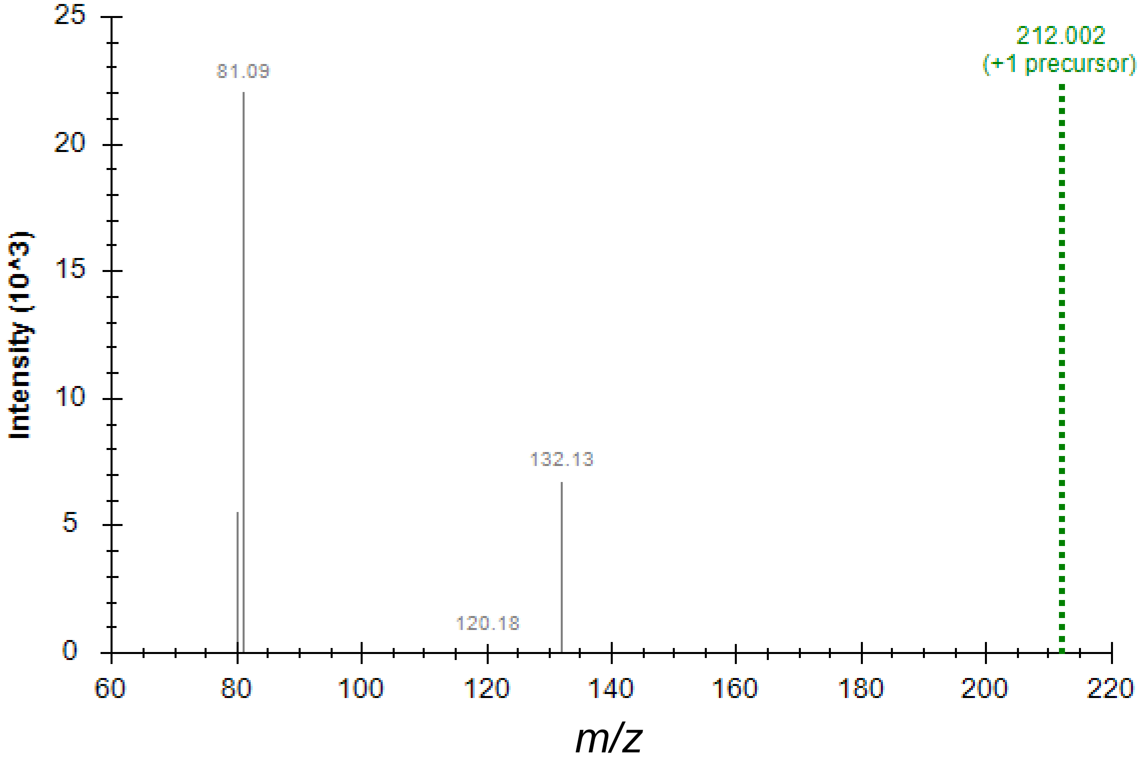

a) MS spectrum of the standard compounds

Ephedrine [M+H]$^+$

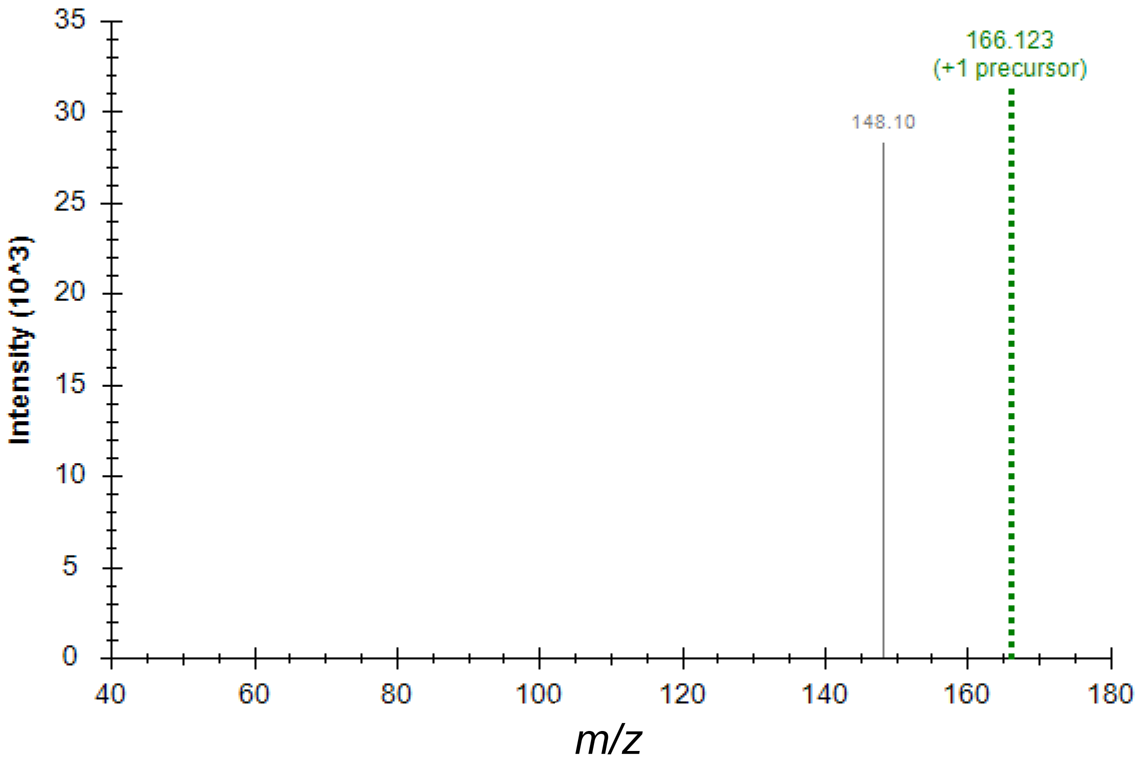

P5938 Scan#:762 [M+H]$^+$

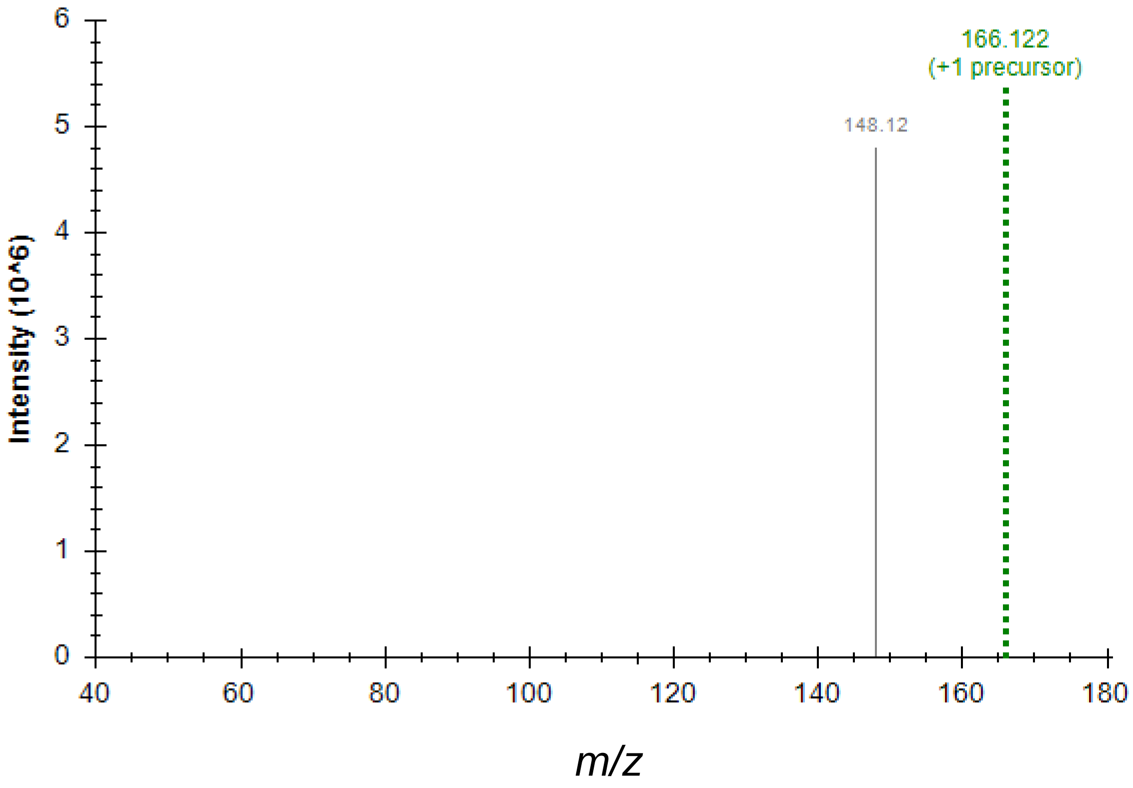

b) Compound identification

Norephedrine [M+H]$^+$

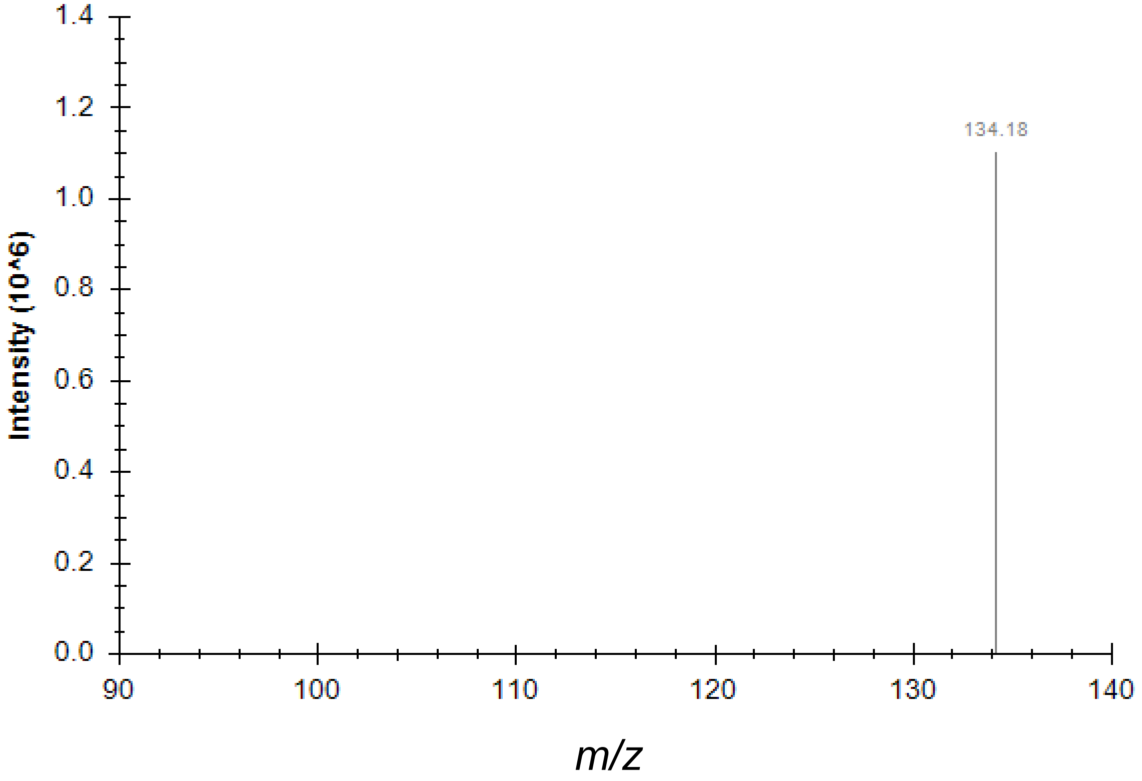

P5302 Scan#:552 [M+H]$^+$

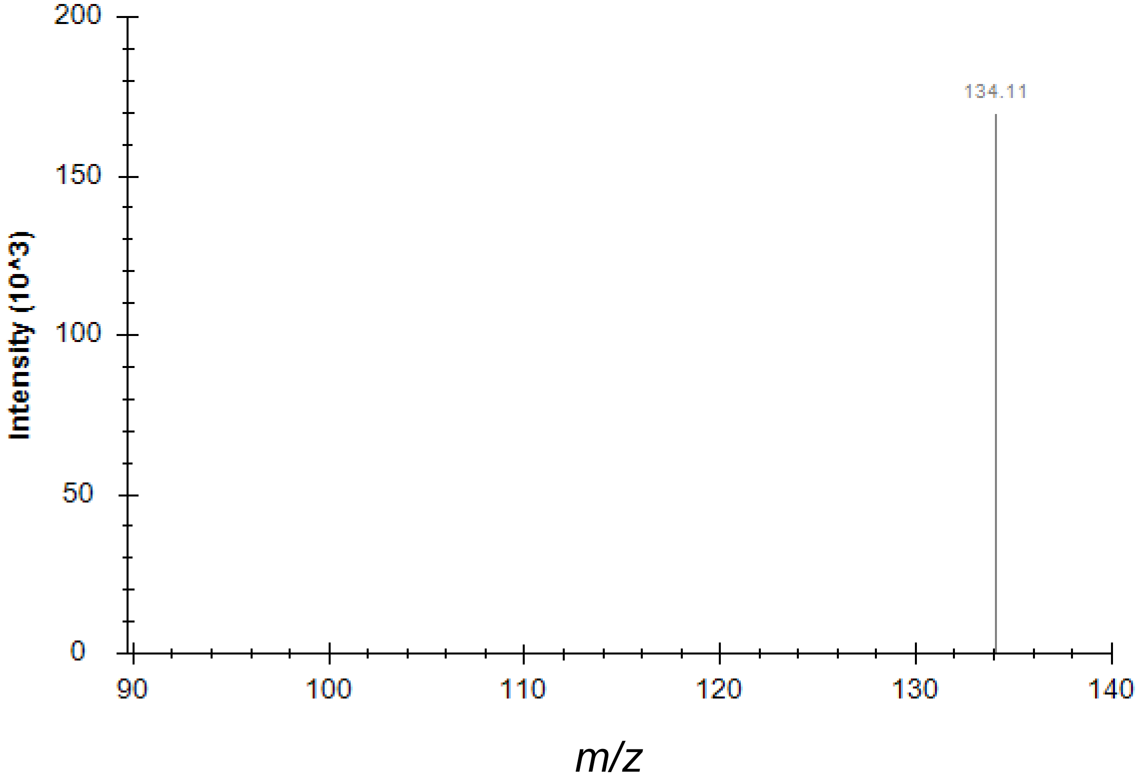

b) Compound identification

Pseudoephedrine $[M+H]^+$

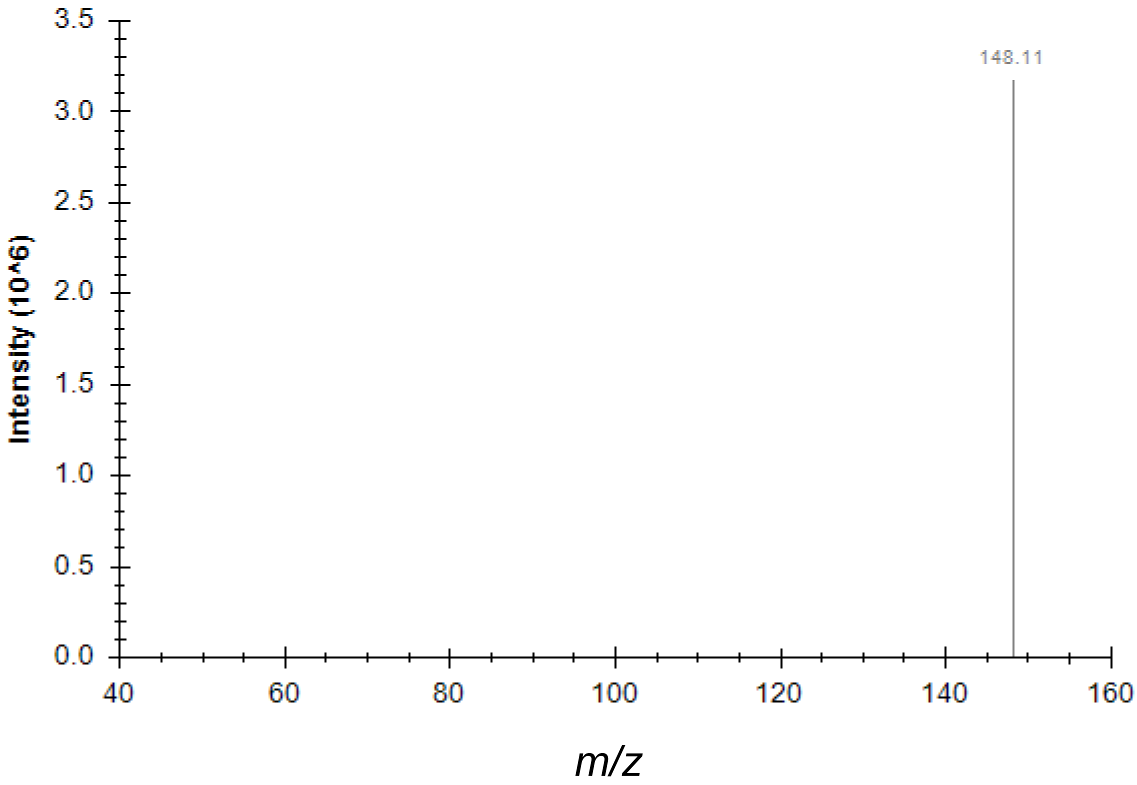

P5302 Scan#:765 $[M-H_2O+H]^+$

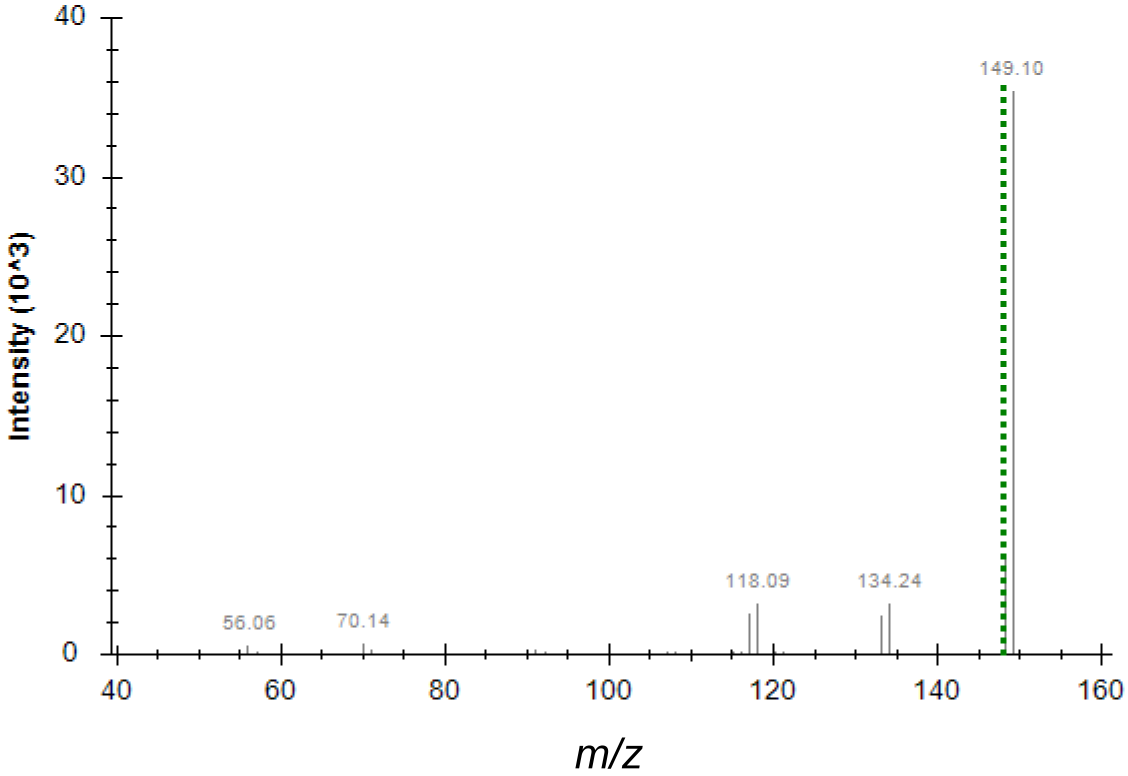

b) Compound identification

Methylephedrine [M+H]$^+$

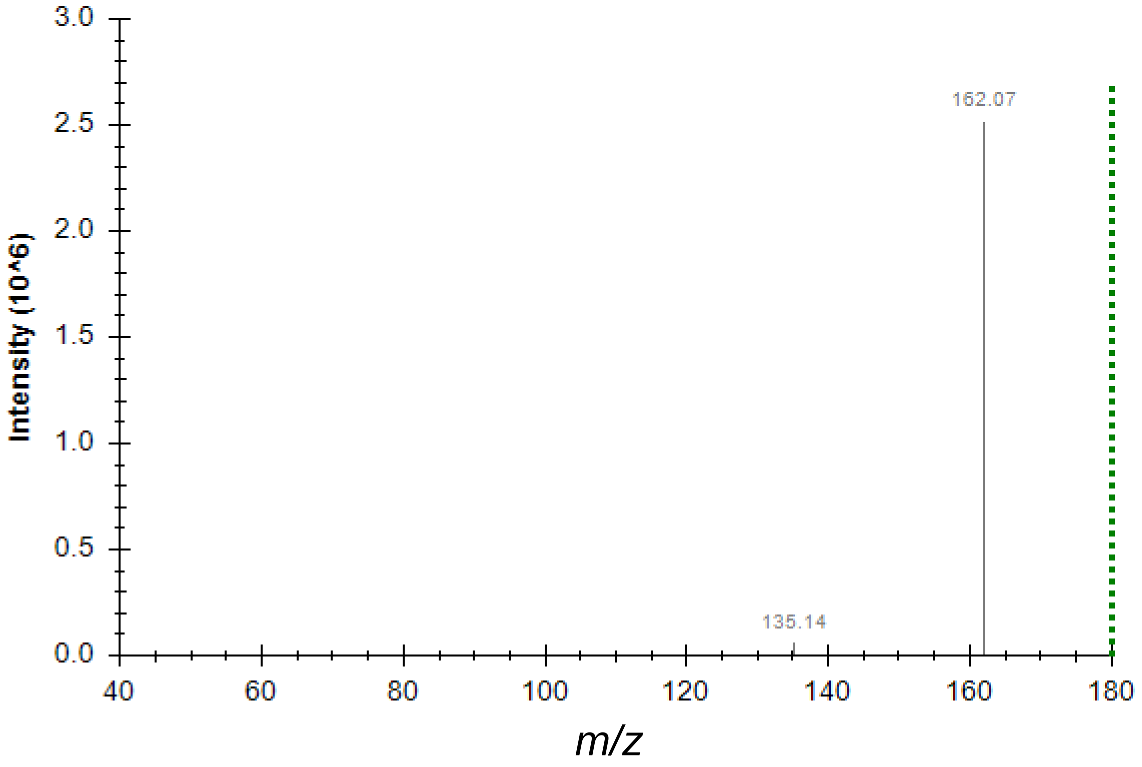

P6310 Scan#:850 [M+H]$^+$

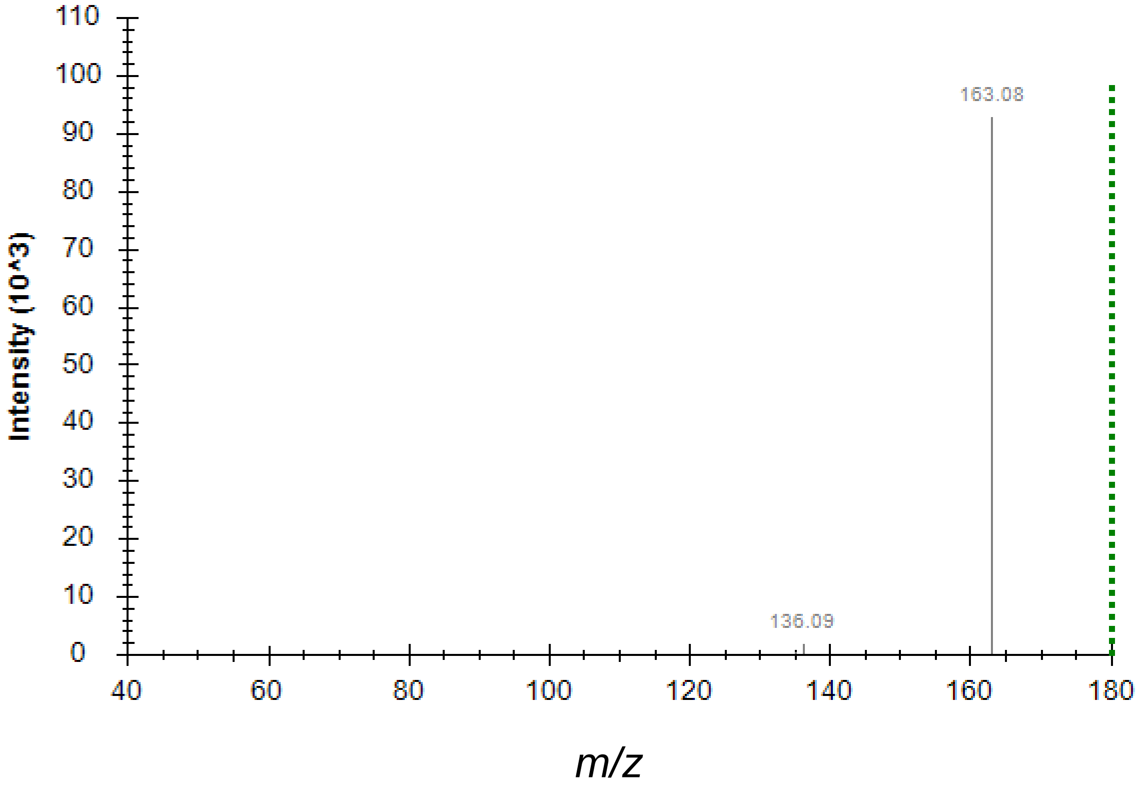

b) Compound identification

Prunasin [M+HCOO]$^-$

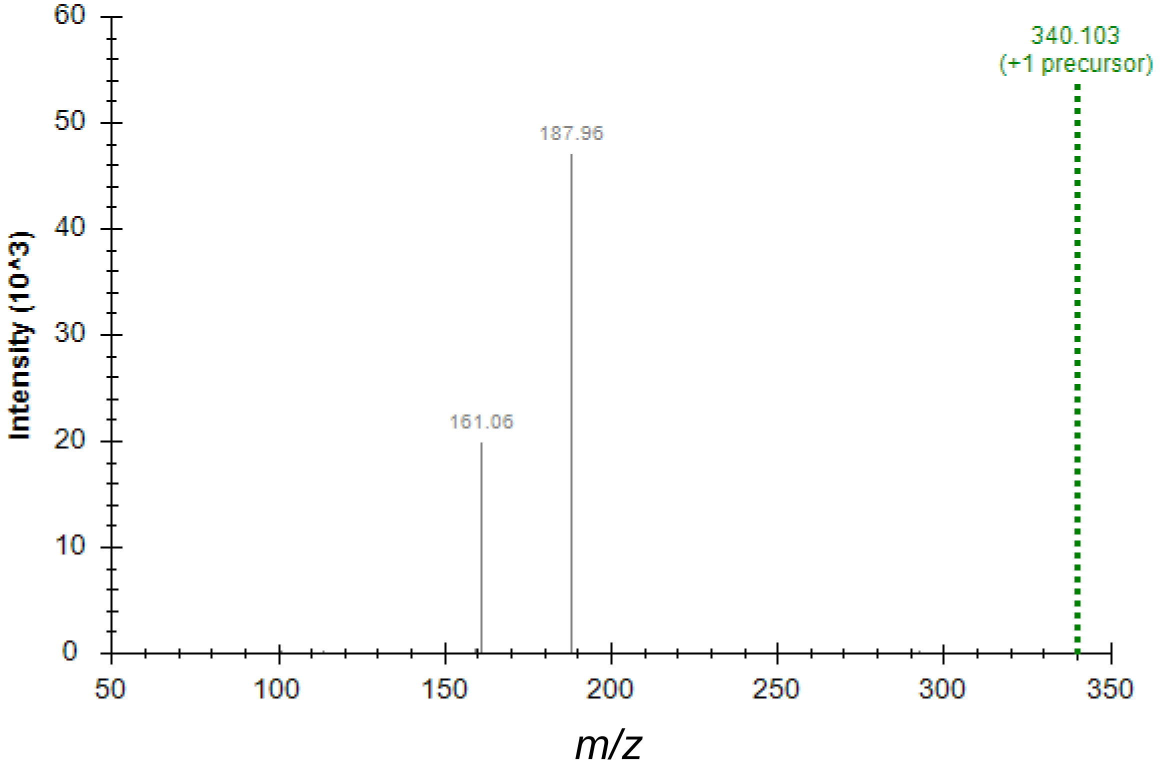

N5336 Scan#:950 [M+HCOO]$^-$

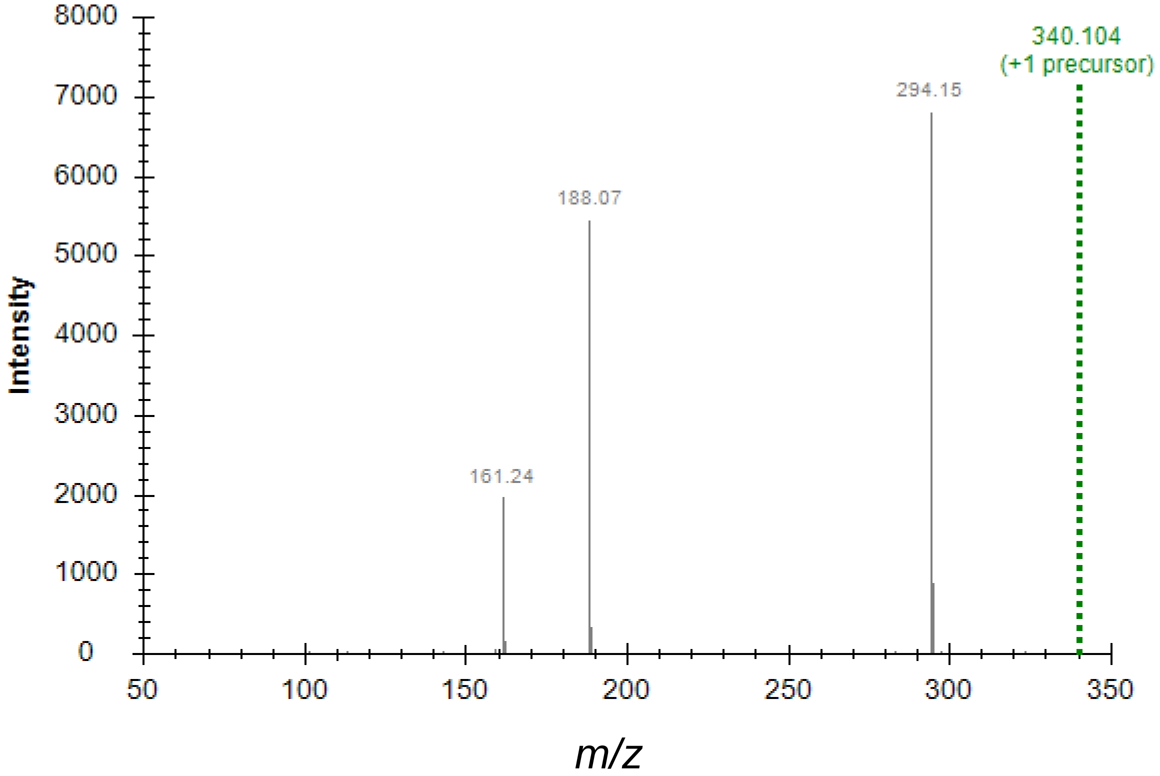

b) Compound identification

Liquiritigenin [M-H]-

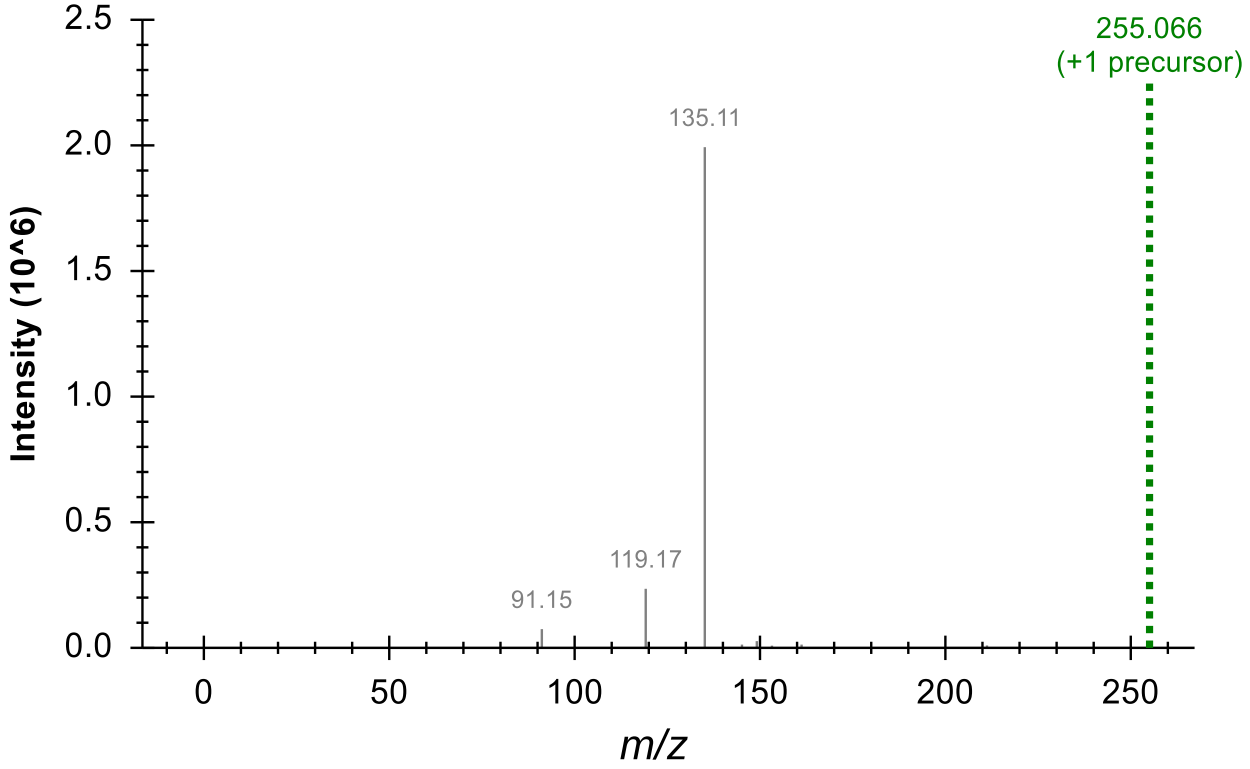

N7137 Scan#:1368 [M-H]-

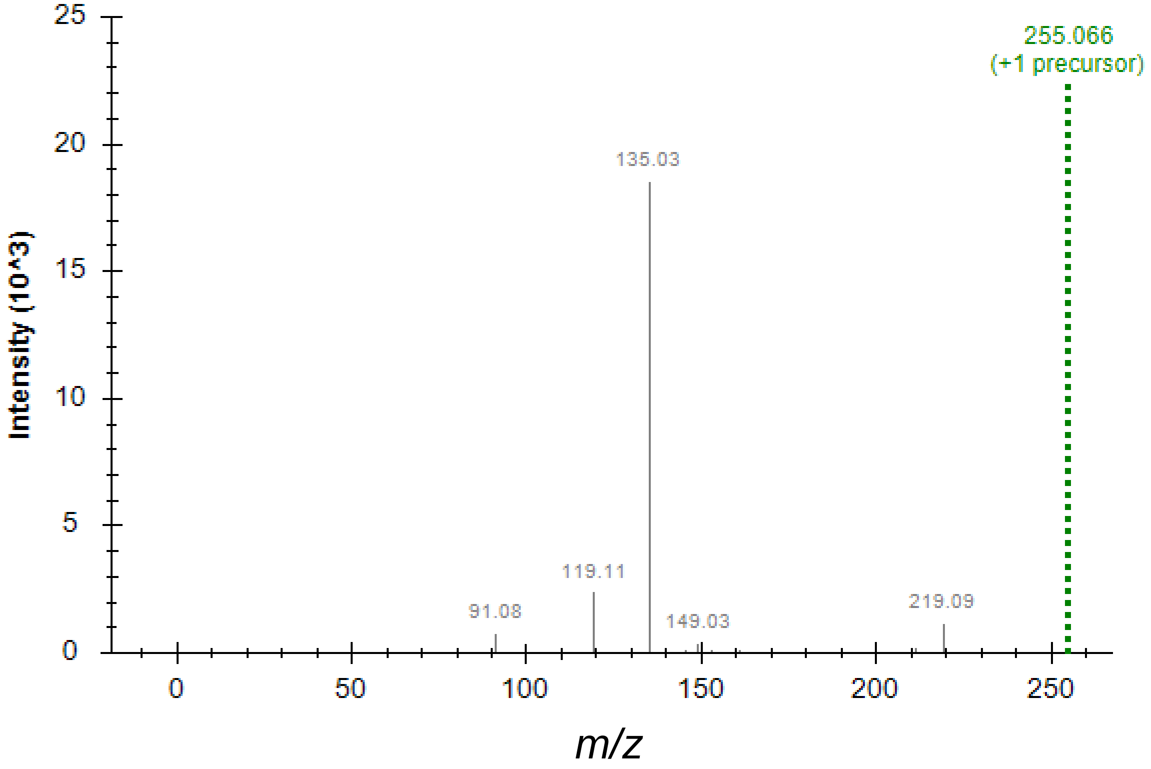

b) Compound identification

Liquiritigenin-4'-glucuronide [M+H]+

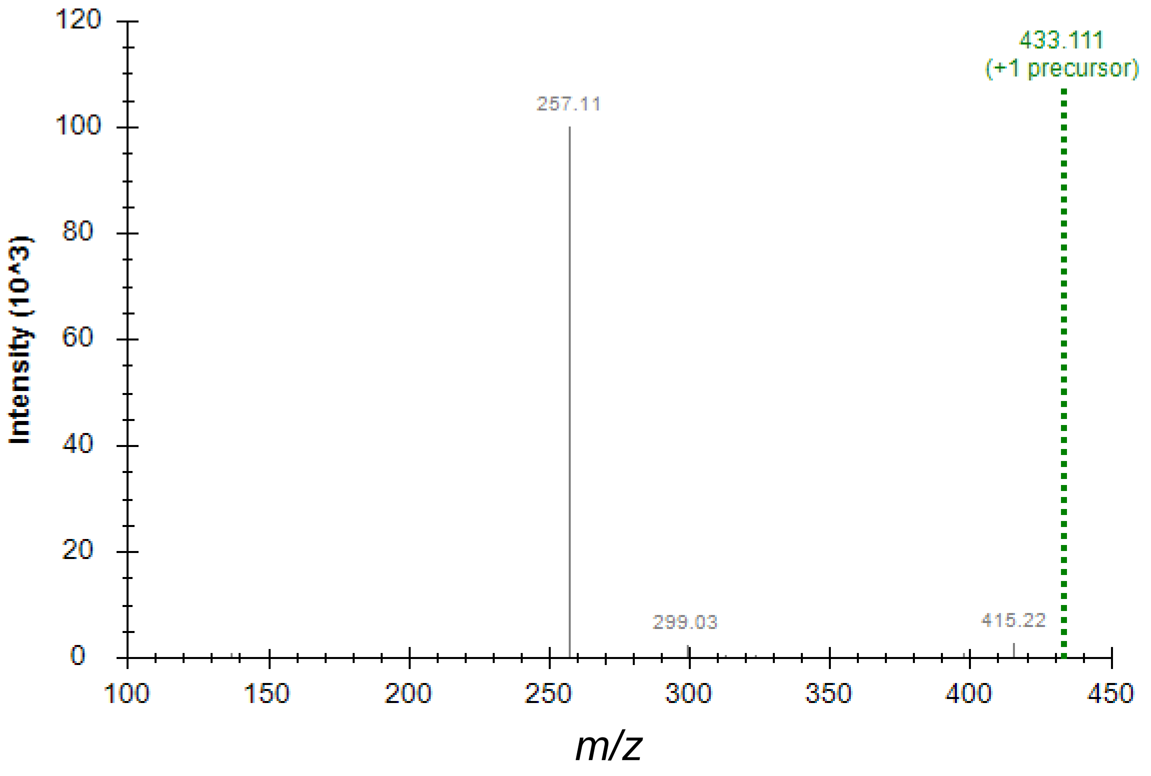

P8722 Scan#:1202 [M+H]+

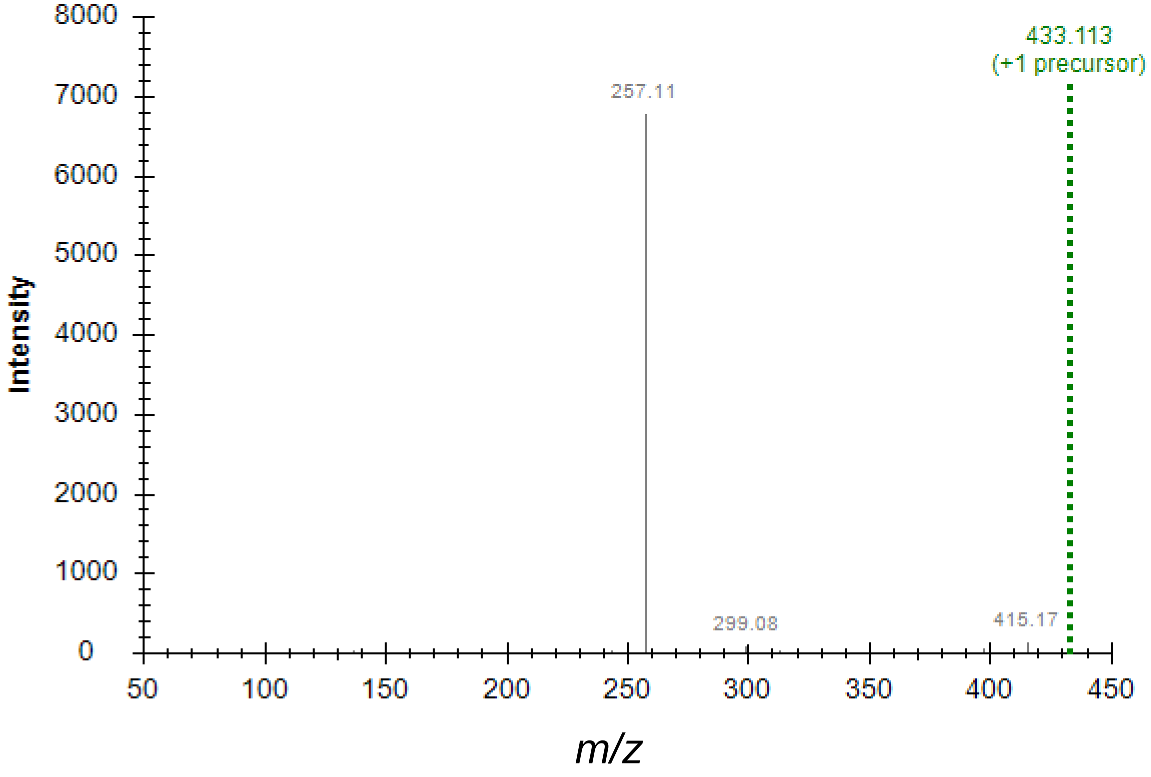

b) Compound identification

Liquiritigenin-4’-glucuronide [M-H]-

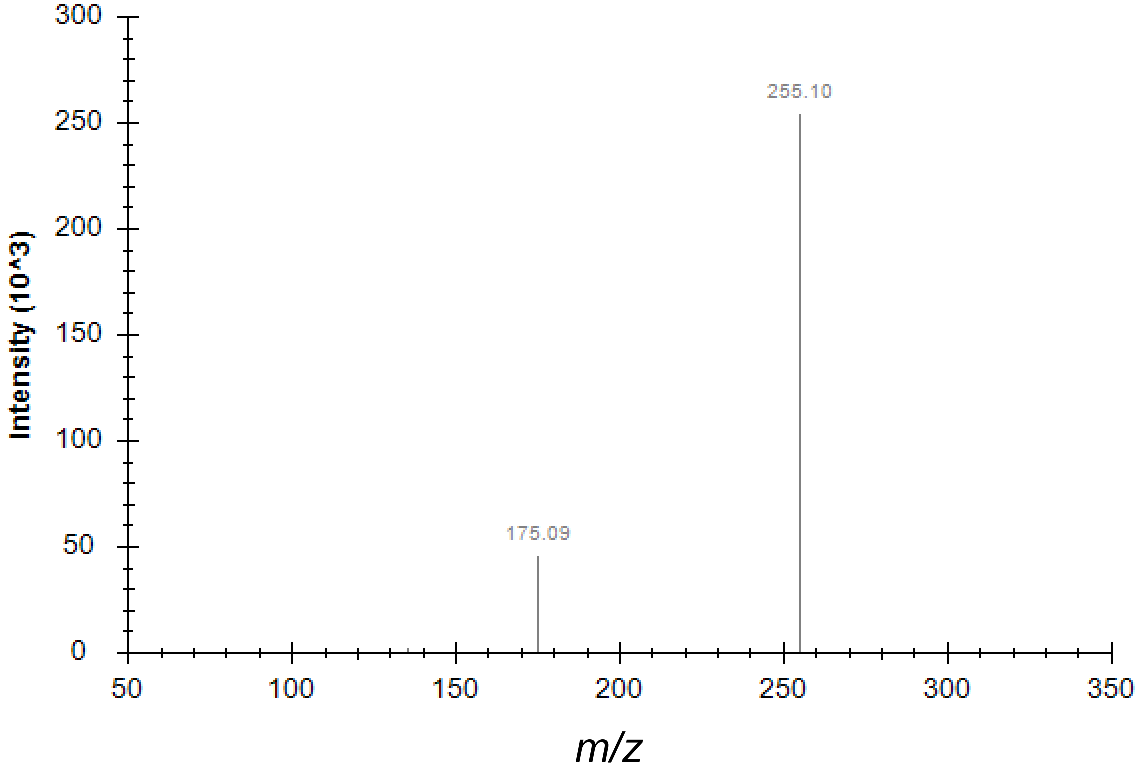

N6061 Scan#:1073 [M+H]+

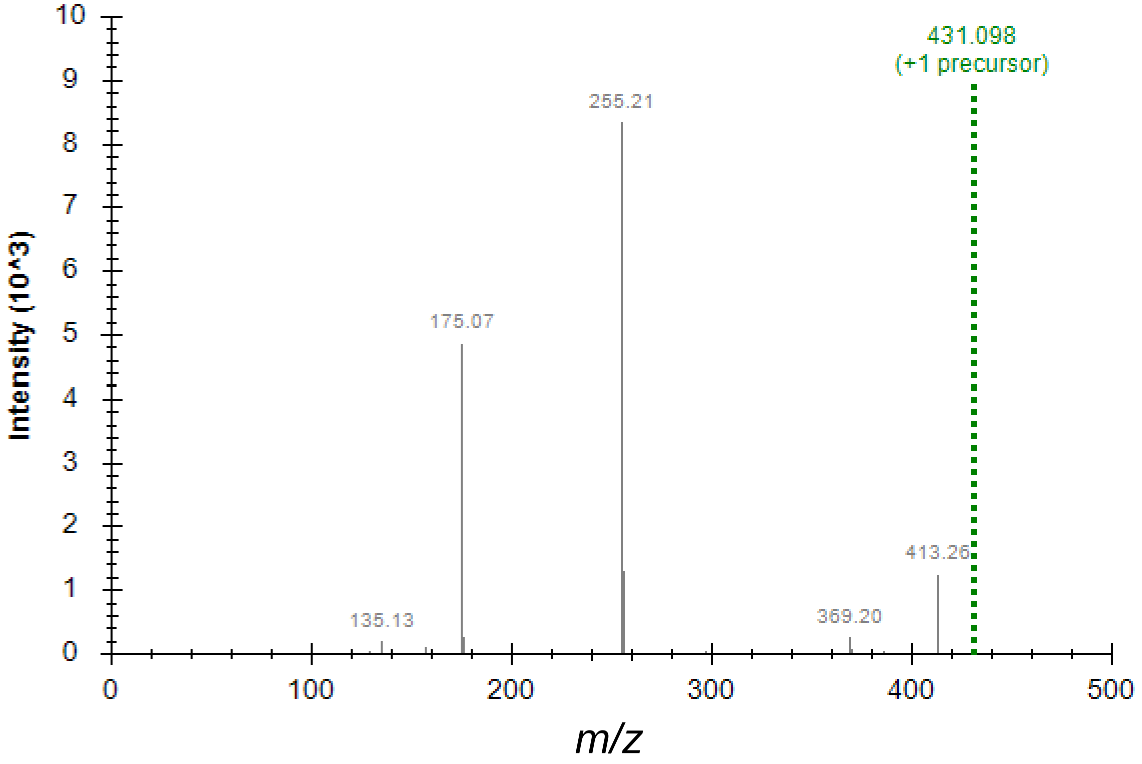

b) Compound identification

N5429 Scan#: 968 $[M-H]^-$

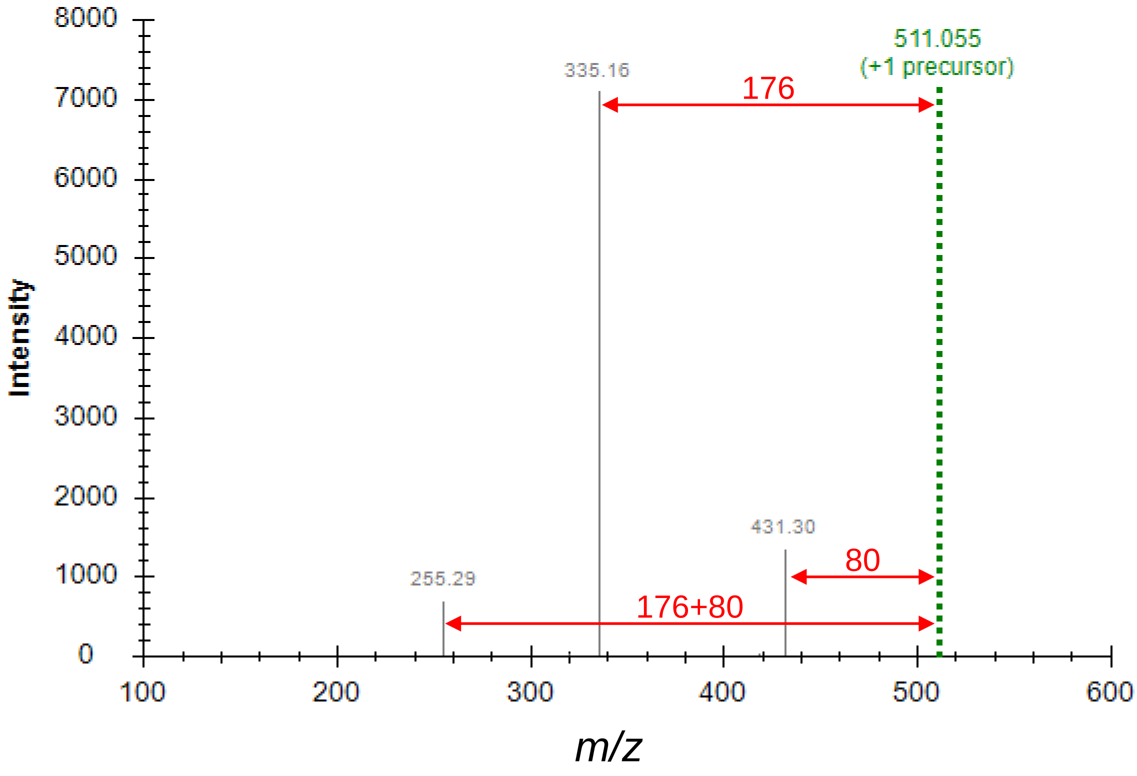

c) MS spectrum of N5429
