# Supplementary material for: Differential annotation of converted metabolites (DAC-Met): Exploration of Maoto (Ma-huang-tang)-derived metabolites in plasma using high-resolution mass spectrometry
Source: Metabolomics. 2020 Apr 25;16(5):63. doi: 10.1007/s11306-020-01681-3 (PMC7183508; doi:10.1007/s11306-020-01681-3)
Supplement: Supplementary file 4 — Mass difference network and subnetwork B-F. Supplementary file4 (PDF 581 kb) [file 11306_2020_1681_MOESM4_ESM.pdf]

Article title:

Differential Annotation of Converted Metabolites (DAC-Met): Exploration of Maoto (Ma-huang-tang)-derived Metabolites in Plasma Using High-resolution Mass Spectrometry.

Journal name: Metabolomics

Author name:

Katsuya Ohbuchi[1]*, Nozomu Sakurai[2,3], Hiroyuki Kitagawa[4], Masaru Sato[3], Hideyuki Suzuki[3], Hirotaka Kushida[1], Akinori Nishi[1], Masahiro Yamamoto[1], Kazuhiro Hanazaki[4], Masanori Arita[2,5]

Affiliation:

[1]*Tsumura Kampo Research Laboratories, Tsumura & CO., Ibaraki 300-1192, Japan*

[2] *National Institute of Genetics, Mishima, Shizuoka 411-8540, Japan*

[3]*Kazusa DNA Research Institute, Kisarazu, Chiba 292-0818, Japan*

[4]*Department of Surgery, Kochi Medical School, Kochi University, Kochi 783-8505, Japan*

[5]*RIKEN Center for Sustainable Resource Science, Yokohama 230-0045, Japan*

[*] Corresponding author.

E-mail: oobuchi_katsuya@mail.tsumura.co.jp (KO)

ORCID: 0000-0001-6756-3260

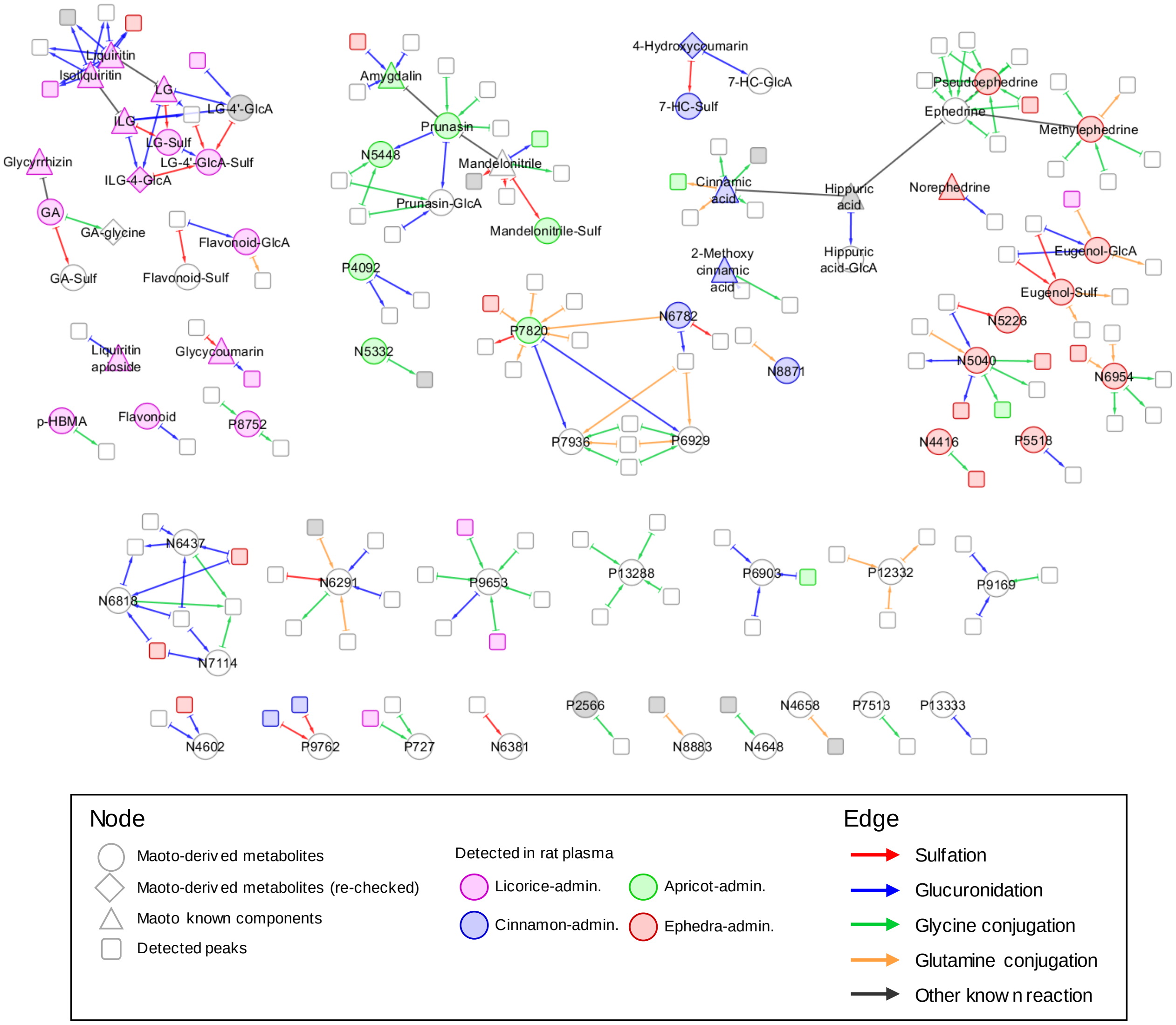

The conjugation network (higher resolution)

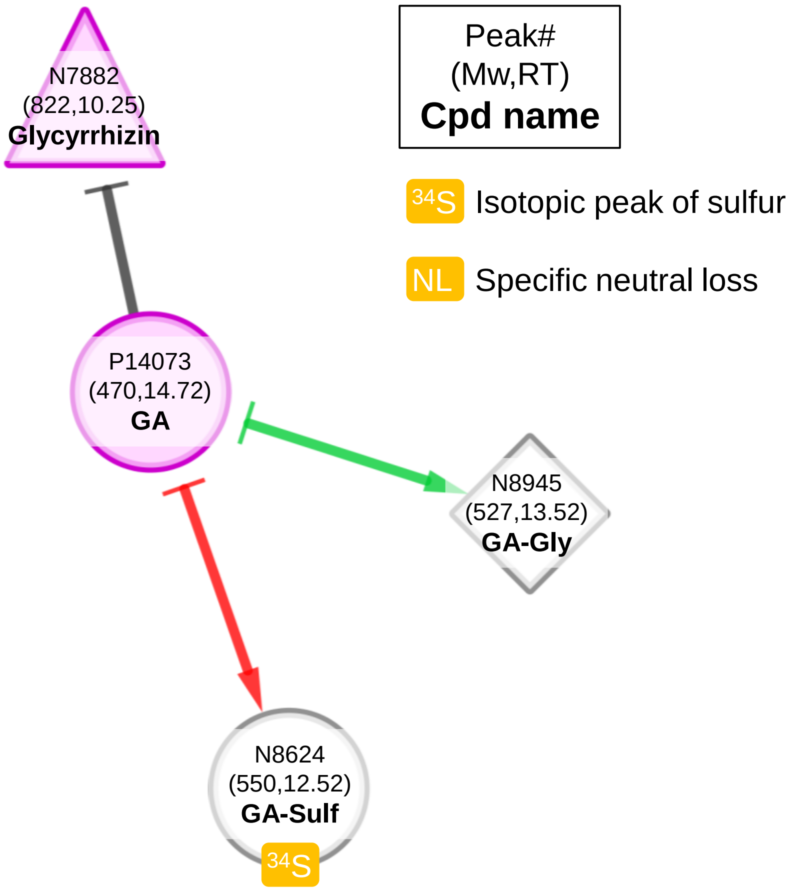

Support information for annotation in subnetwork B

Subnetwork B consists of the peaks derived from glycyrrhizin. The microbiome produces GA from glycyrrhizin. Mass difference analysis demonstrated that glycyrrhetinic acid (GA) is transformed into GA sulfate (N8624) and GA glycine (N8945). Both metabolites were maoto-derived metabolites and N8624 had a clear stable isotopic signal of sulfur.

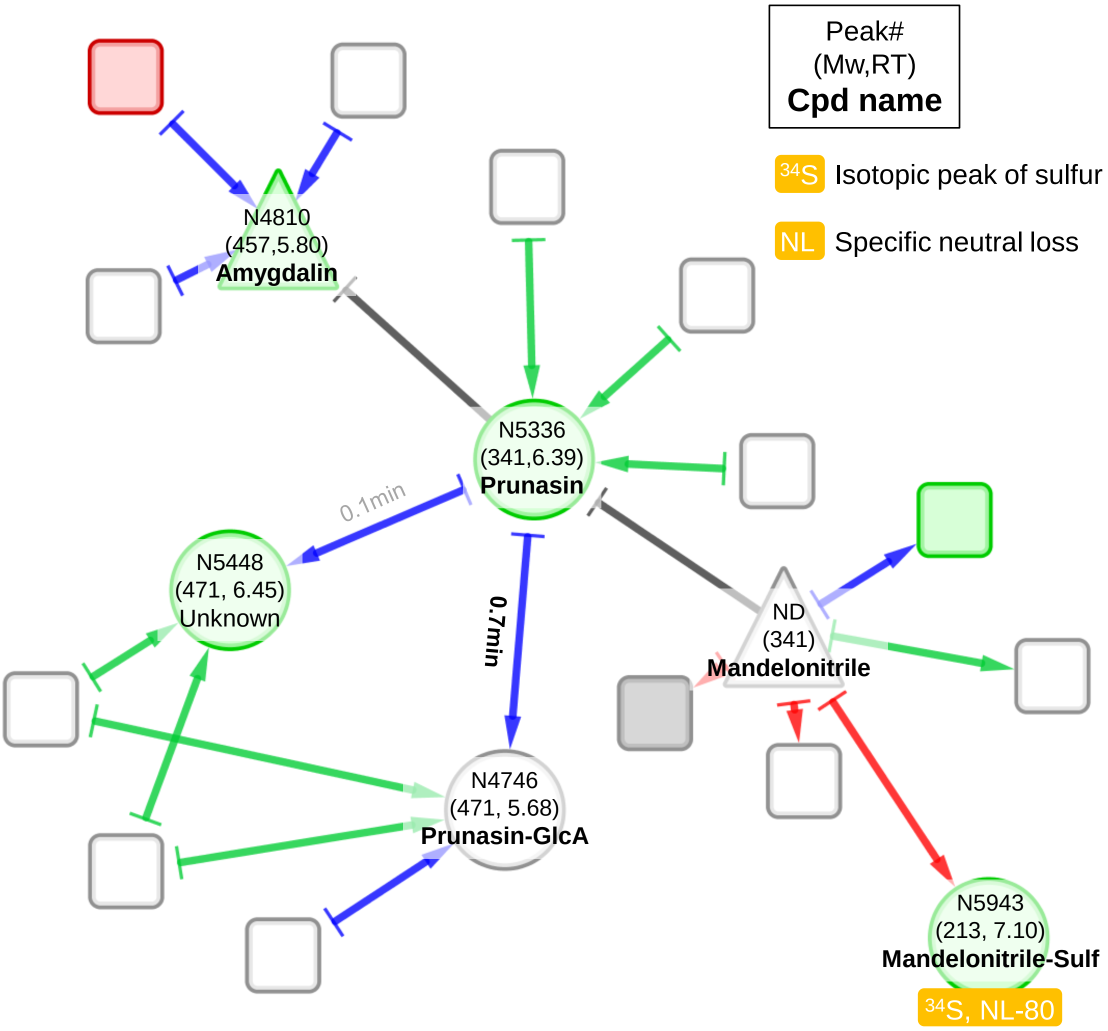

Support information for annotation in subnetwork C

Subnetwork C consisted of the peaks derived from an apricot kernel. Prunasin (N5336) is produced from amygdalin (N4810), which is a well-known ingredient of apricot kernels (Strugala et al., 1995), and converted to mandelonitrile. Two maoto-derived compounds, N4746 and N5448, qualified as prunacin-glucuronide from the mass difference, and N4746 was chosen based on the RT shift. Prunasin is converted into mandelonitrile, which was not detected in this study. N5943 might be a sulfate of mandelonitrile according to the mass difference and the ^{34}S peak (labelled with ^{34}S). Indoxyl sulfate was another candidate for this peak, but this was ruled out by the chemical standard.

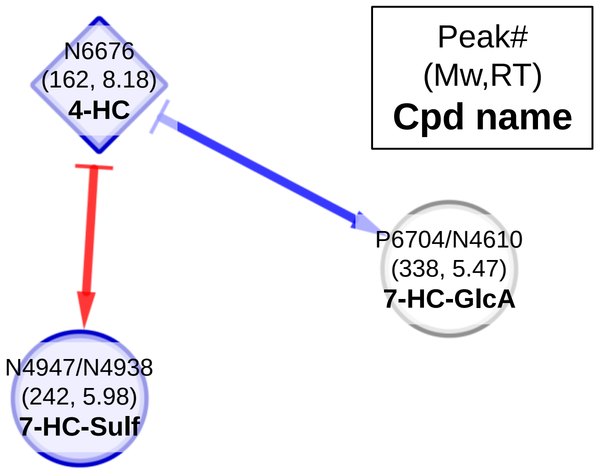

Support information for annotation in subnetwork D

Subnetwork D contained 3 maoto-derived compounds that were estimated to be hydroxycoumarin (HC) analogues or their derivatives from the database search (N4947, *m/z* 241; N4938, *m/z* 161; and N4610, *m/z* 337). The RT of N4938 was identical to that of N4947 and was considered as its in-source fragment losing a sulfate. When N4938 was estimated as an HC sulfate, P6704/N4610 was an HC glucuronide according to its mass difference. By comparison with commercially available standard compounds of HCs (4-HC, 6-HC, 7-HC, 7-HC-glucuronide, and 7-HC-sulfate), N4938/N4947 and P6704/N4610 were identified as 7-HC-sulfate and 7-HC-glucuronide (MSI level-1). Although 7-HC was not detected in the plasma samples, it showed a small signal in the maoto extract.

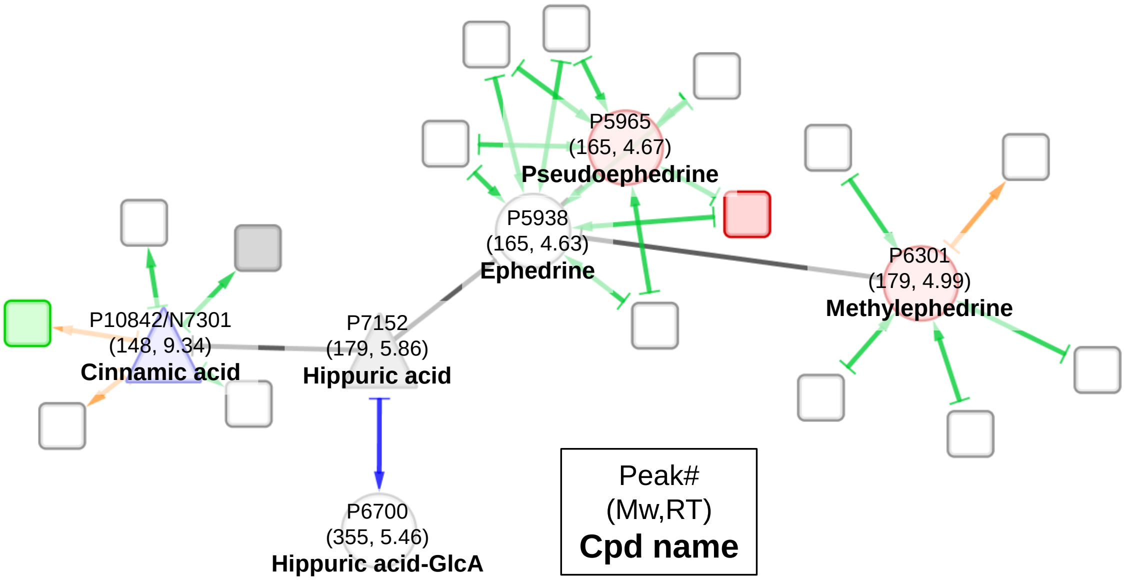

Support information for annotation in subnetwork E

Cinnamic acid and ephedrine are metabolized into hippuric acid. Hippuric acid would be transformed into its glucuronide form.

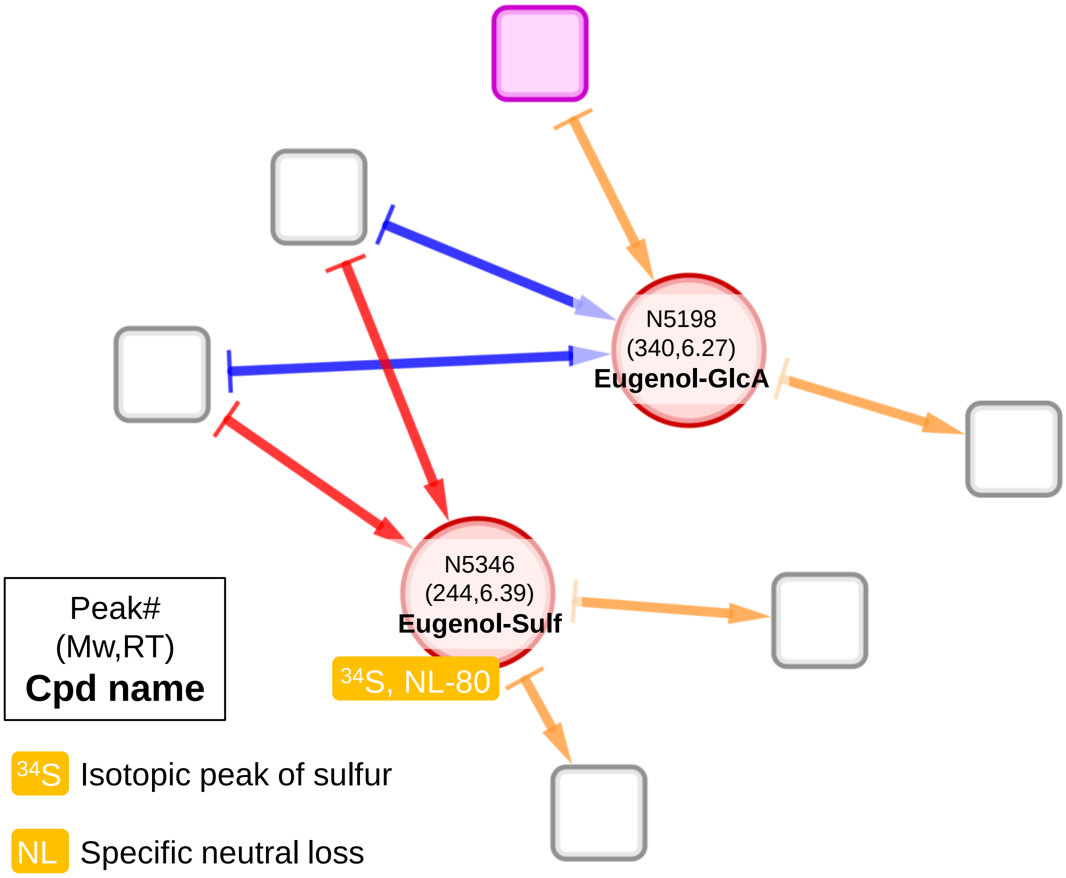

Support information for annotation in subnetwork F

Subnetwork F also contained maoto-derived compounds that were estimated to be sulfate (N5346) and glucuronide (N5198). The mass of the unconjugated form corresponded to eugenol, the main component of oil of ephedra (Maggi et al., 2011). Eugenol is volatile and difficult to measure using LC-MS but its metabolites appear to be detectable. The result of annotation following DAC-Met is summarized in Fig. 2b. Finally, together with the literature knowledge, we reconstructed the metabolic pathway of maoto components (Fig. 5).

Reference

Maggi, F., Nicoletti, M., Petitto, V., Sagratini, G., Papa, F., Vittori, S., 2011. Solid-phase microextraction (SPME) analysis of six Italian populations of Ephedra nebrodensis Tineo ex Guss. subsp. nebrodensis. Chem. Biodivers. 8, 95–114.

Strugala, G.J., Stahl, R., Elsenhans, B., Rauws, A.G., Forth, W., 1995. Small-intestinal transfer mechanism of prunasin, the primary metabolite of the cyanogenic glycoside amygdalin. Hum. Exp. Toxicol. 14, 895–901.
