# Supplementary material for: Differential annotation of converted metabolites (DAC-Met): Exploration of Maoto (Ma-huang-tang)-derived metabolites in plasma using high-resolution mass spectrometry
Source: Metabolomics. 2020 Apr 25;16(5):63. doi: 10.1007/s11306-020-01681-3 (PMC7183508; doi:10.1007/s11306-020-01681-3)
Supplement: Supplementary file 5 — Kinetic time-course of maoto components and metabolites represented as mean values of 4 human individuals. Supplementary file5 (PDF 89 kb) [file 11306_2020_1681_MOESM5_ESM.pdf]

**Article title:**

# Differential Annotation of Converted Metabolites (DAC-Met): Exploration of Maoto (Ma-huang-tang)-derived Metabolites in Plasma Using High-resolution Mass Spectrometry.

Journal name: Metabolomics

Author name:

Katsuya Ohbuchi[1]*, Nozomu Sakurai[2,3], Hiroyuki Kitagawa[4], Masaru Sato[3], Hideyuki Suzuki[3], Hirotaka Kushida[1], Akinori Nishi[1], Masahiro Yamamoto[1], Kazuhiro Hanazaki[4], Masanori Arita[2,5]

Affiliation:

[1]*Tsumura Kampo Research Laboratories, Tsumura & CO., Ibaraki 300-1192, Japan*

[2] *National Institute of Genetics, Mishima, Shizuoka 411-8540, Japan*

[3]*Kazusa DNA Research Institute, Kisarazu, Chiba 292-0818, Japan*

[4]*Department of Surgery, Kochi Medical School, Kochi University, Kochi 783-8505, Japan*

[5]*RIKEN Center for Sustainable Resource Science, Yokohama 230-0045, Japan*

[*] Corresponding author.

E-mail: oobuchi_katsuya@mail.tsumura.co.jp (KO)

ORCID: 0000-0001-6756-3260

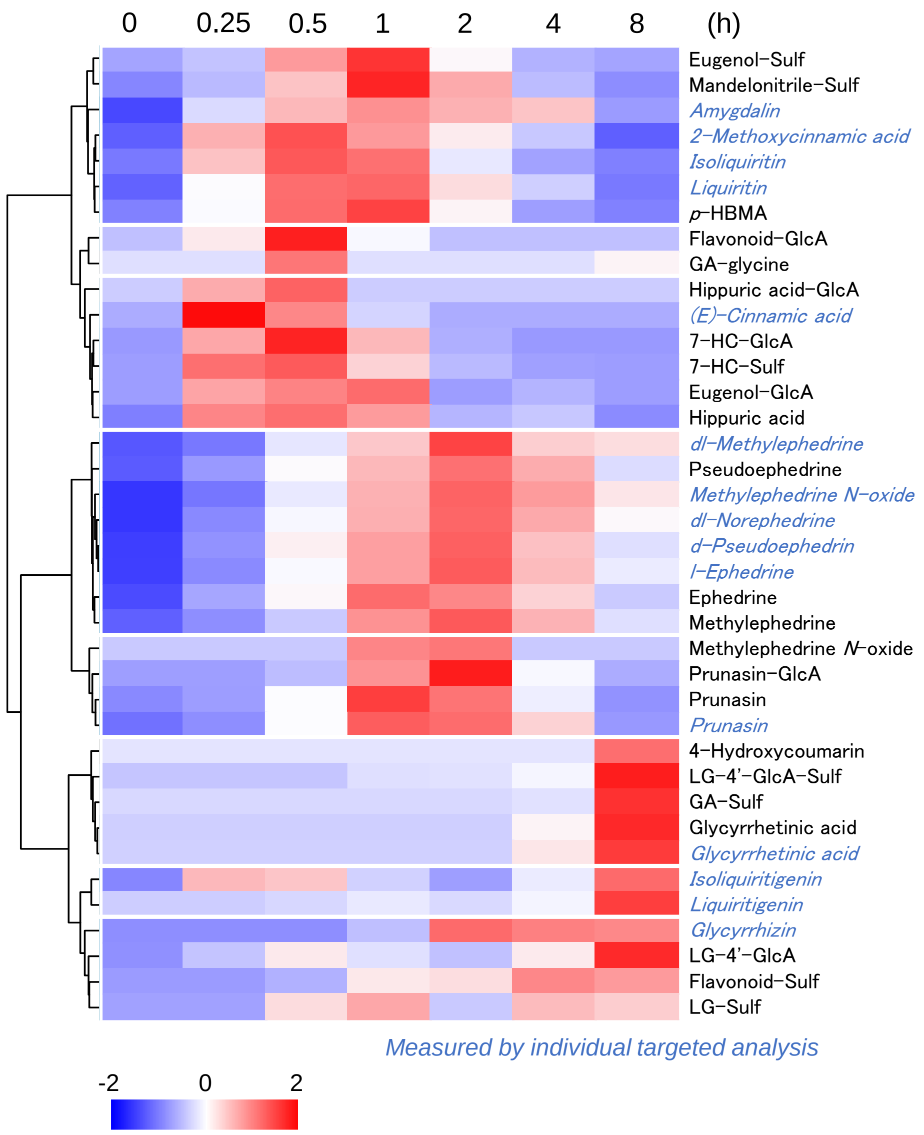


**Supplementary Material 5 Kinetic time-course of maoto components and metabolites represented as mean values of 4 human individuals**

A heat map consisting of the mean values of the four test subjects was developed for intuitive understanding of the trends of each component. The blue components represented plasma concentration was used from a previous report (Kitagawa et al., 2019). The mean values were calculated based on the clustering results in Figure 6.

## Reference

Kitagawa, H., Ohbuchi, K., Munekage, M., Fujisawa, K., Kawanishi, Y., Namikawa, T., Kushida, H., Matsumoto, T., Shimobori, C., Nishi, A., Sadakane, C., Watanabe, J., Yamamoto, M., Hanazaki, K., 2019. Data on metabolic profiling of healthy human subjects' plasma before and after administration of the Japanese Kampo medicine maoto. Data Br. 22, 359–364.
